# *O*-glycosylation pattern of the SARS-CoV-2 spike protein reveals an “O-Follow-N” rule

**DOI:** 10.1038/s41422-021-00545-2

**Published:** 2021-08-02

**Authors:** Wenmin Tian, Delin Li, Nan Zhang, Guijie Bai, Kai Yuan, Haixia Xiao, Feng Gao, Yang Chen, Catherine C. L. Wong, George Fu Gao

**Affiliations:** 1 Center for Precision Medicine Multi-Omics Research, Peking University Health Science Center, Peking University, Beijing, China; 2 School of Basic Medical Sciences, Peking University Health Science Center, Beijing, China; 3 Laboratory of Protein Engineering and Vaccines, Tianjin Institute of Industrial Biotechnology, Chinese Academy of Sciences (CAS), Tianjin, China; 4 Shanxi Academy of Advanced Research and Innovation, Taiyuan, Shanxi China; 5 Peking-Tsinghua Center for Life Sciences, Beijing, China; 6 Peking University First Hospital, Beijing, China; 7 Advanced Innovation Center for Human Brain Protection, Capital Medical University, Beijing, China; 8 CAS Key Laboratory of Pathogen Microbiology and Immunology, Institute of Microbiology, Chinese Academy of Sciences, Beijing, China; 9 National Institute for Viral Disease Control and Prevention, Chinese Center for Disease Control and Prevention (China CDC), Beijing, China

**Keywords:** Proteomic analysis, Glycosylation

Dear Editor,

Severe acute respiratory syndrome coronavirus-2 (SARS-CoV-2), the causative agent of coronavirus disease 2019 (COVID-19), emerged in late 2019 and has since caused a pandemic. Although there have been extensive studies worldwide, our understanding of this newly emerged pathogen is far from sufficient. The pathogenesis of the SARS-CoV-2 infection is not fully understood, although a “two-stage” hypothesis was proposed in our previous study.^1^

As a member of enveloped virus in family *Coronaviridae*, SARS-CoV-2 makes use of a densely glycosylated spike (S) protein to gain entry into host cells. The S protein is a trimeric class I transmembrane protein composed of two functional subunits. The S1 subunit binds to cellular angiotensin-converting enzyme 2 (ACE2) for host cell recognition, and the S2 subunit functions in viral–host cell membrane fusion. The S protein is the most attractive immunogen for eliciting antibody responses and is therefore the primary focus for neutralizing antibody and vaccine development.

The glycosylation of viral envelope proteins has a wide range of functions, including regulating viral tropism, protein stability and shielding the underlying epitopes from immune surveillance. Thus, a full understanding of the glycosylation of SARS-CoV-2 S protein is critical to reveal the pathogenesis of the virus and to guide the design of therapeutic and prophylactic strategies. A total of 22 *N*-glycosites were mapped in the in vitro-expressed S protein ectodomain and the S protein extracted from virions.^2^ Due to the technical limitations, only a few *O*-glycosylation modifications were confirmed on purified S protein^2–5^ and none has been reported on the S protein extracted from SARS-CoV-2 virions, which is the most representative antigen on virions.

To obtain a comprehensive *N*- and *O*-linked glycosylation landscape of the S protein at its native status including glycosites, glycoforms, and the relative intensity, we extracted S protein from the SARS-CoV-2 virions and purified recombinant full-length wild-type (WT) S protein expressed in human embryonic kidney 293T cells (Supplementary information, Fig. S1a, b). To generate glycopeptides and ensure maximum coverage of the protein sequence, the S protein was digested separately with chymotrypsin, α-lytic endopeptidase or LysC-trypsin. The glycopeptides were analyzed using nano liquid chromatography (nLC) coupled with an ultra-high resolution Orbitrap Eclipse Tribrid mass spectrometer, and stepped collisional energy (SCE) HCD and HCDpdEThcD were applied for fragmentation. The data were processed by software Byonic (v3.8.13, Protein Metrics Inc., Cupertino) and Byologic glyco-analysis software (v3.8-11 ×64, Protein Metrics Inc., Cupertino). We specifically applied multiple approaches for the *O*-glycosylation analysis and site confirmation. First, an additional treatment with PNGase F in O^18^ water after protease digestion was carried out for *N*-glycan removing, in which deamidation of asparagine (Asn) yielded an aspartic acid residue with a mass shift of +2.98 Da. It discriminated modified *N*-glycosites from unoccupied Asn or glutamine (Gln) thus excluded the interference of *N*-glycosylation on the identification of *O*-glycosylation. Second, we conducted simultaneous search on *N*- and *O*-linked glycans together in the same samples. In order to conclusively rule out artifactual assignment of *N*-linked glycan to nearby Ser (S) or Thr (T), we used four criteria for the characterization of *O*-glycopeptides, including (1) the MS/MS spectra contains glycans or oxonium ions (i.e., feature B ions); (2) the MS/MS spectra contains feature Y ions; (3) isotope distribution of precursor is reasonable; and (4) the retention time of glycopeptides and non-glycopeptides is comparable. We did extensive manual validation in order to select the valid *O*-glycopeptides for the specific site analysis.^6^ Diagnostic ions were used as the requisite criterion for the *O*-glycosite determination.

In line with the previous study, we identified 22 *N*-glycosites with confirmation (Fig. 1a; Supplementary information, Fig. S2 and Dataset S1).^7^ For the first time, a total of 17 *O*-glycosites were identified on the S protein extracted from SARS-CoV-2 virions (Fig. 1a; Supplementary information, Fig. S3, Table S1 and Dataset S1), among which 14 sites were determined with diagnostic ions (Fig. 1a; Supplementary information, Fig. S3 and Table S1). The *O*-glycoforms of the S protein extracted from virions are diverse and in sharp difference with the reported glycoforms of purified S protein (Fig. 1b).^5^ We found that *O*-glycosylation occurred in clusters on the S protein. The S1 domain was more *O*-glycosylated with 11 sites, while the remaining 6 sites were detected at the N-terminal of the S2 domain (Fig. 1a). Interestingly, 11 out of 17 identified *O*-glycosites located near glycosylated Asn, including S60, T124, S151, T236, T604/S605, T618, S659, T1076, T1077, S1097 and T1100 (Fig. 1a, c; Supplementary information, Fig. S3 and Table S1). The glycopeptide containing T604 and S605 sites was well characterized, however, we were not able to determine the exact glycosylation site due to a lack of diagnostic ions. Therefore, we counted T604/S605 as one *O*-glycosite.

**Fig. 1:**
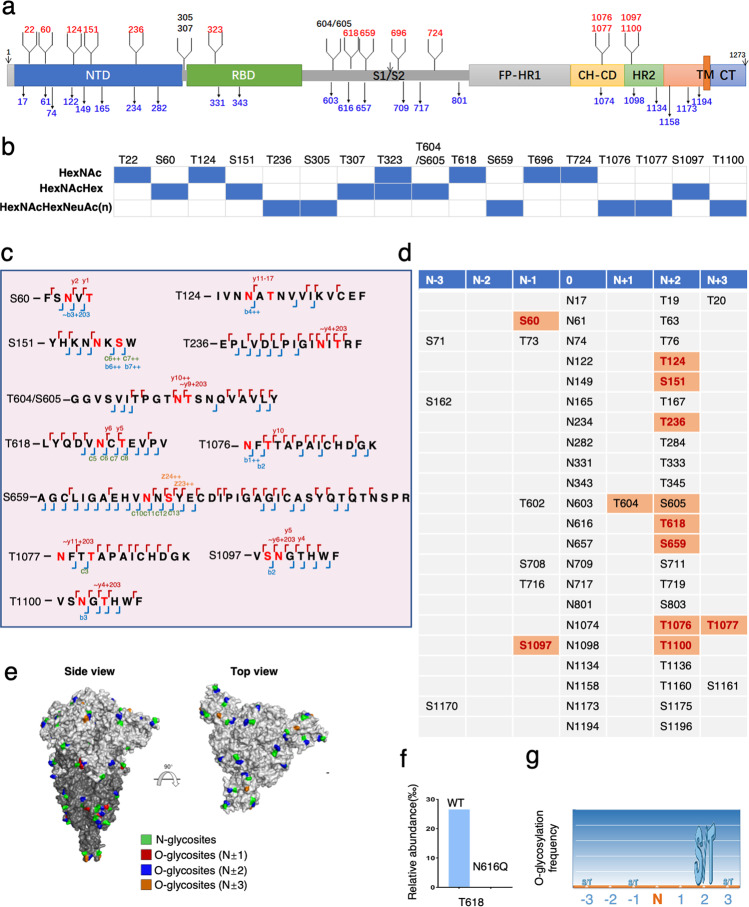
*O*-glycosylation of SARS-CoV-2 S protein complies with the “O-Follow-N” rule. **a** Schematic illustration of the S glycoprotein. The arrows underneath with blue numbers indicate the 22 identified *N*-glycosites. The branches above with numbers mark the 17 identified *O*-glycosites. T604/S605 was counted as one *O*-glycosite due to a lack of diagnostic ions. The determined *O*-glycosites are labeled in red color. NTD, N-terminal domain; RBD, receptor-binding domain; FP, fusion peptide; HR1, heptad repeat 1; HR2, heptad repeat 2; CH, central helix; CD, connector domain; TM, transmembrane domain; CT, cellular tail. **b** The composition of different *O*-glycoforms in S protein. HexNAcHexNeuAc(n) represents glycoforms with 1–3 NeuAc. **c** The identified *O*-glycopeptides of the S protein extracted from SARS-CoV-2 virions. The modified Asn and glycosylated S/T are marked in red color. b- or y- product ion fragments are marked as blue or red corners, respectively. c- or z- product ion fragments are annotated in orange or blue on the respective spectra. The diagnostic ions are labeled above and/or below the amino acid. T618 and S659 were identified in the sample with PNGase F de-*N*-glycosylation treatment, using Asn deamidation (+2.988261) and *O*-glycosylation as variable modifications for database search. S60, T124, S151, T236, T604/S605, T076, T1077, S1097 and T1100 were all identified in the sample without PNGase F treatment which were fragmented using SEC HCD. *N*- and *O*-glycosylation were searched simultaneously at the same peptide. **d** The overview of S and T sites of the S protein at N ± 1–3 positions flanking *N*-glycosites. 0 represents the position of glycosylated Asn. Identified *O*-glycosites are highlighted with orange color, and the determined sites are marked in red. **e** Structure-based display of the *N*- and *O*-glycosites in the S protein. Glycosites are marked on the top and side views of the structure of the trimeric SARS-CoV-2 S glycoprotein (PDB ID 6XR8). The *N*-glycosites are marked in green, while the *O*-glycosites at N ± 1, N ± 2, or N ± 3 are marked with red, blue, and brown, respectively. The surfaces of the S1 and S2 subunits are displayed in light and dark gray, respectively. **f** A bar graph showing the comparison of the relative *O*-glycosylation intensities on T618 in WT and N616Q S proteins. The relative intensity is calculated by normalizing the extracted chromatographic areas of *O*-glycopeptides containing T618 to the total extracted chromatographic areas of the sample. **g** Illustration of the *O*-glycosylation frequency at the proximal position associated to *N*-sequon.

In order to further investigate the dynamics between *N*- and *O*-linked glycosylation, we defined the three amino acids on each side of the glycosylated Asn within the consensus motif of NxS/T (x is not proline (P)) as the “position associated to N-sequon” (named N ± 1–3). There are 35 S/T within positions associated to N-sequon; 11 of them were *O*-glycosylated among which 10 sites were determined. It is intriguing that 7 out of the 10 sites (70%) were located at the N + 2 position, which is in the consensus motif of *N*-glycosylation (Fig. 1d). All the identified *N*-glycosites and *O*-glycosites associated to N-sequon were mapped on the surface of S protein based on the cryo-EM structure of the trimeric SARS-CoV-2 S protein (Protein Data Bank (PDB) ID 6XR8) (Fig. 1e).

To further validate the phenomenon that *N*- and the *O*-linked glycosylation occurred together in N-sequon-associated positions, we carried out site-directed mutagenesis. An N-to-Q mutation was generated on the *N*-linked glycosite N616 on purified full-length WT S protein. The *O*-glycosite T618 was analyzed together with the deamidated N616 (Supplementary information, Fig. S4). Mutations of N616 completely abolished the *O*-glycosylation on T618 (Fig. 1f), indicating that the presence of glycosylated Asn is the prerequisite of *O*-glycosylation associated to N-sequon.

Based on the observations above, we proposed an “O-Follow-N” rule, whereby *O*-glycosylation occurs near the glycosylated Asn in N-sequon. This may also apply to other proteins and promote the identification of *O*-glycosites (Fig. 1g). It has been reported that GalNAc-transferases (GalNAc-Ts), which mediate the initiation of mucin-type *O*-glycosylation, contain a lectin-like domain that binds glycans.^8^ We reasoned that GalNAc-Ts may recognize glycans on the *N*-glycosites, thus catalyzing *O*-glycosylation near the *N*-glycosites.

In summary, we conducted a site-specific glycosylation analysis, and comprehensively profiled the *N*- and *O*-linked glycosylation of the S protein either extracted from virions or in vitro expressed. To our knowledge, this is the first and largest glycosylation dataset of S protein directly extracted from the SARS-CoV-2 virions to date, which broadens our understanding of the glycosylation of the SARS-CoV-2 S protein. In this context, we observed a unique pattern that *O*-glycosites were located near glycosylated Asn in N-sequon. This phenomenon was also observed by Sanda et al. on purified S protein and on other proteins by other research groups,^4,9,10^ but no rule has been proposed due to the limited number of identified sites.

The observation reported here of *O*-glycosites in close proximity to *N*-glycosylation suggests the possible “O-Follow-N” rule. It has long been known that *N*-glycosylation occurs in the NxS/T (x is not proline (P)) consensus motif, however, it is not clear whether the S/T after glycosylated Asn is prone to be *O*-glycosylated. If this is the case, it would be interesting to explore whether the glycosylation of S/T depends on the nearby *N*-glycosylation. The “O-Follow-N” rule discovered in this study would shed light on the potential new mechanisms of *O*-glycosylation, especially the synergies between *N*- and *O*-glycosylation, and would greatly benefit fundamental glycobiology studies.

## Supplementary information

Supplementary information

Supplementary Dataset 1

## Data Availability

The mass spectrometry raw files have been deposited in the MassIVE proteomics database under the accession number PXD023346.
