# Supplementary material for: O-glycosylation pattern of the SARS-CoV-2 spike protein reveals an “O-Follow-N” rule
Source: Cell Res. 2021 Aug 2;31(10):1123–5. doi: 10.1038/s41422-021-00545-2 (PMC8326647; doi:10.1038/s41422-021-00545-2)
Supplement: Supplementary file 1 — Supplementary information [file 41422_2021_545_MOESM1_ESM.pdf]

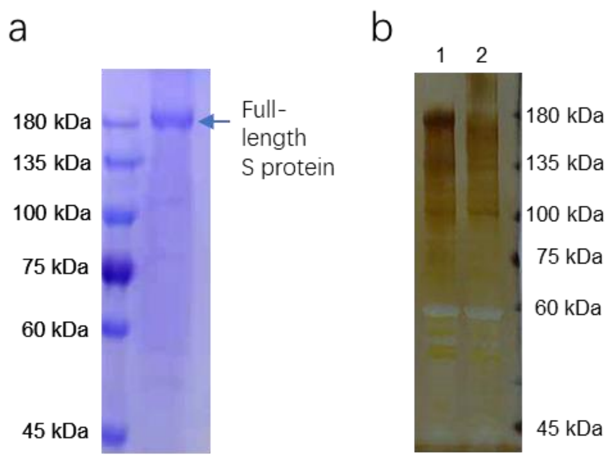

Fig. S1. Gel images of the S protein of SARS-CoV-2

a. SDS-PAGE gel of the S protein expressed in human embryonic kidney 293T cells. The band of the full-length of S protein is indicated by blue arrow.

b. Silver staining gel of the S protein extracted from virions. Lane 1, purified SARS-CoV-2 virus; Lane 2, purified SARS-CoV-2 virus treated with PNGas F. The band close to the 180 kDa in lane 1 is the S protein.

N17

Sample = SARS-COV-2 Virus S _ Lytic_ full_309N 2021-4-11

Sequence = QCVNLT; Mods = NGlycan/1548.5448/HexNAc(3)Hex(4)NeuAc(1)

z=2

Error (ppm) = -1.53

Retention Time (min) = 81.02

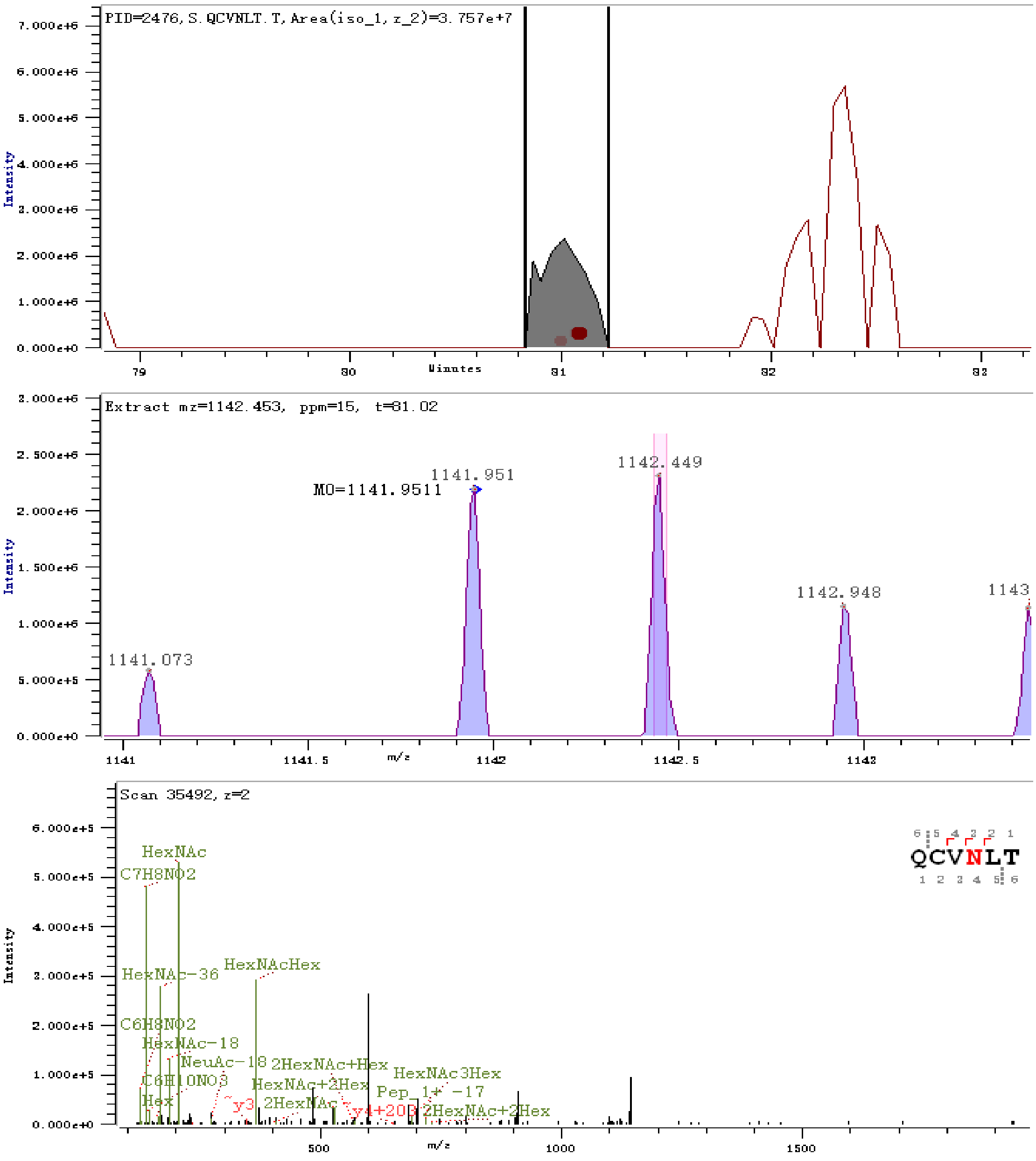

N61

Sample = SARS-COV-2 Virus S _ chymotrypsin_ full_309N 2021-4-11

Sequence = FSNVTW; Mods = NGlycan/1378.4757/HexNAc(2)Hex(6)

z=2

Error (ppm) = 0.51

Retention Time (min) = 60.15

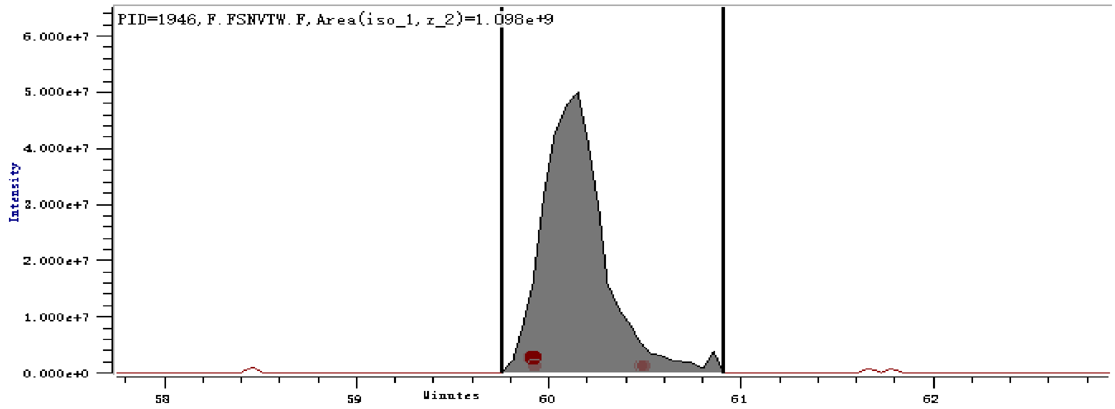

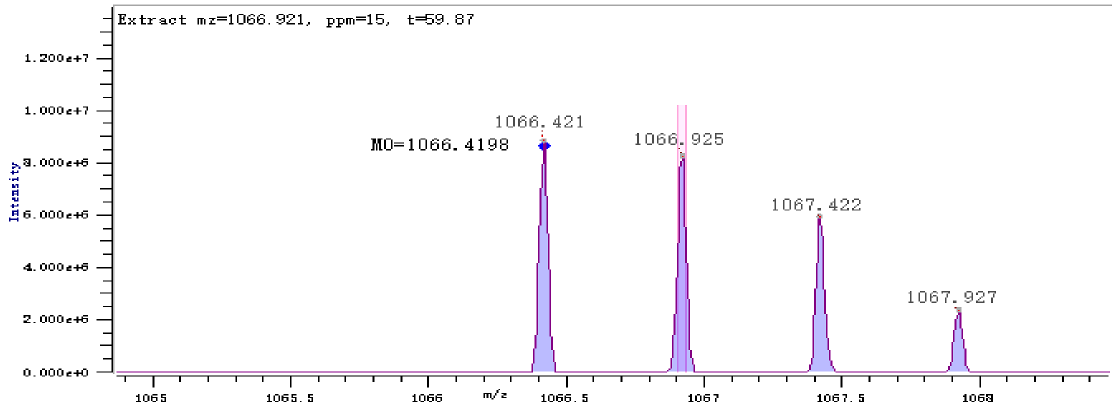

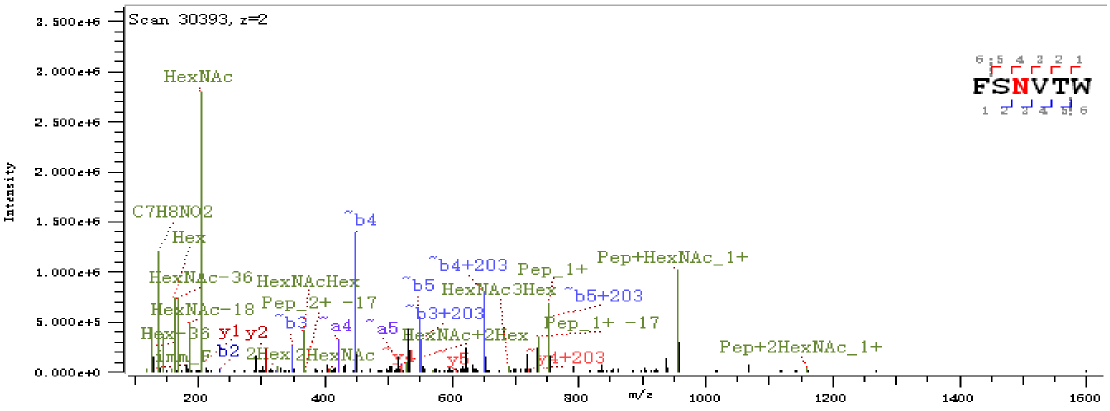

N74

Sample = SARS-COV-2 Virus S _deglycan_ O18_ chymotrypsin_ semi_78O 2021-4-11

Sequence = HAIHVSGTNGTKRFDNPVLPF; Mods = Deamidated/2.9883/

z=4

Error (ppm) = -0.76

Retention Time (min) = 85.64

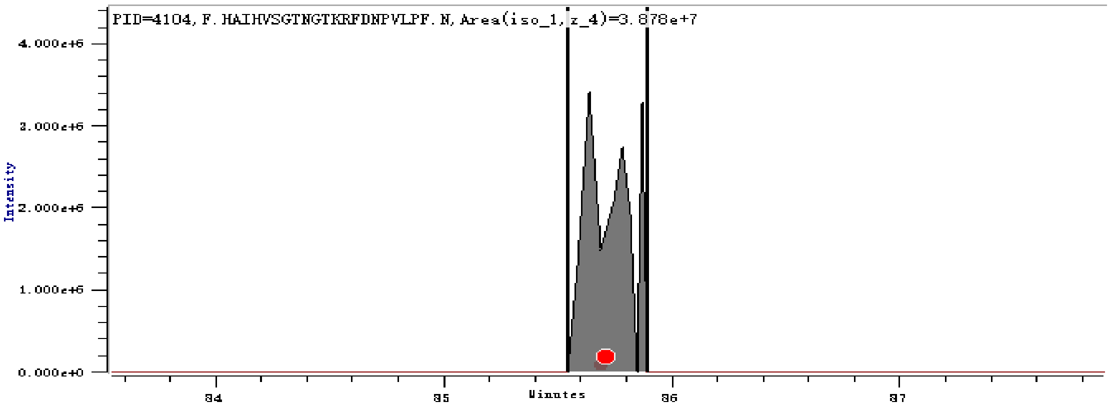

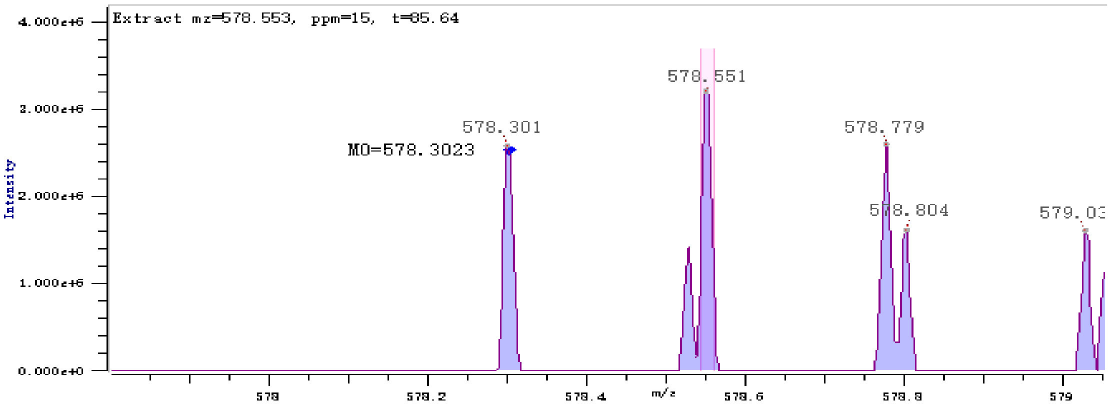

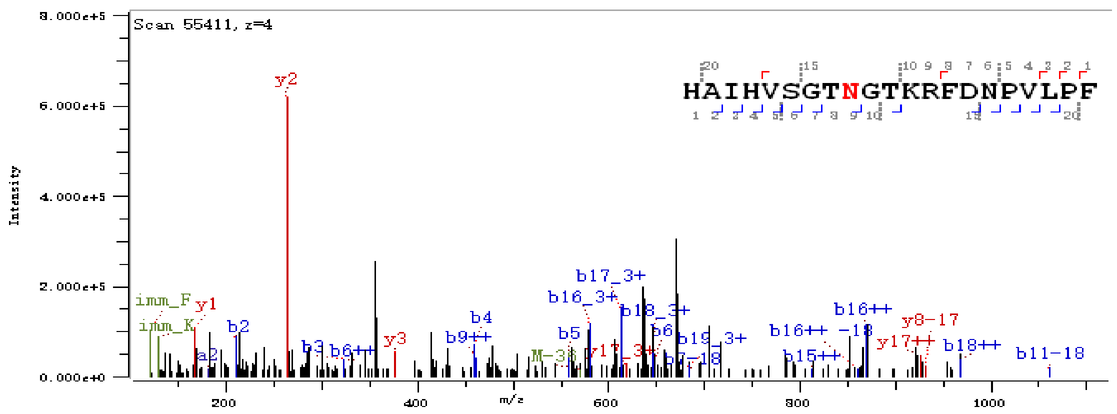

N122

Sample = SARS-COV-2 Virus S _trypsin_ full_309N 2021-4-11

Sequence = TQSLLIVNNATNVVIK; Mods = NGlycan/1540.5285/HexNAc(2)Hex(7)

z=2

Error (ppm) = 2.73

Retention Time (min) = 100.26

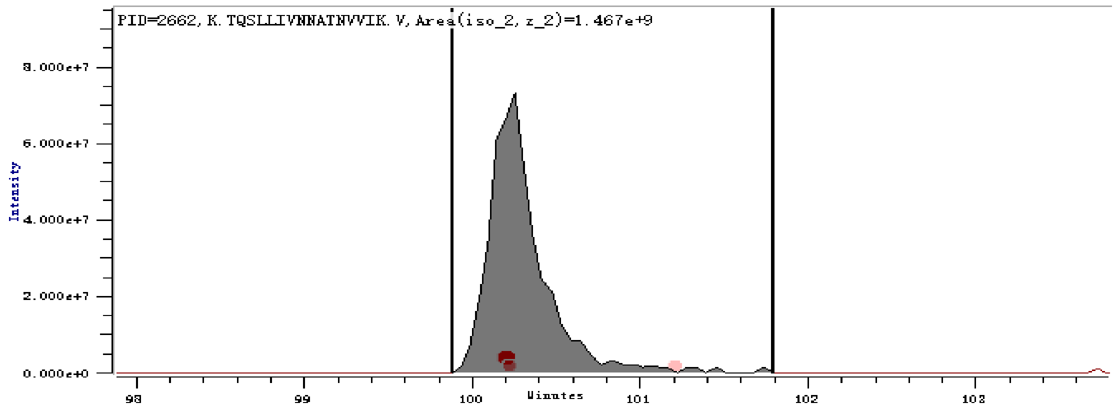

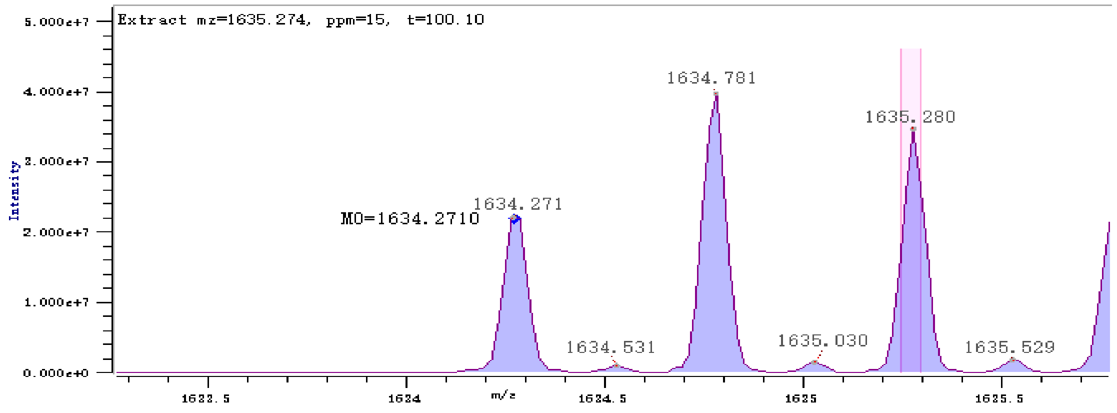

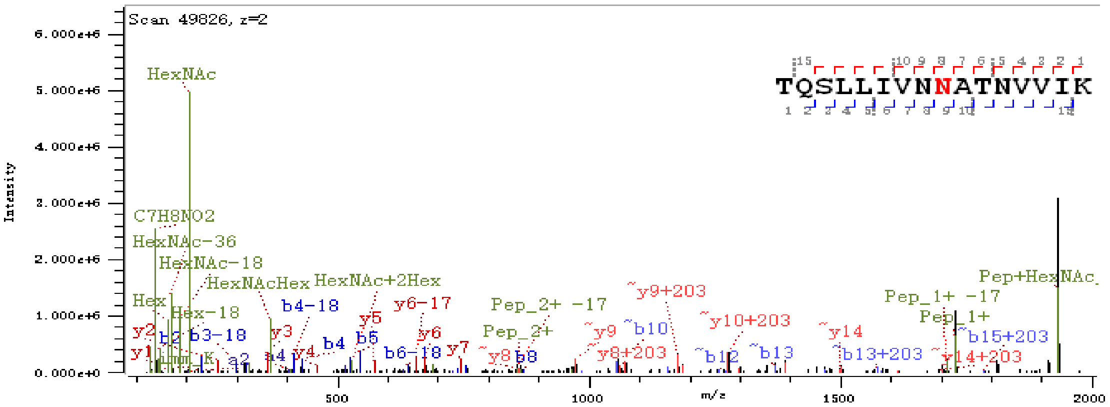

N149

Sample = SARS-COV-2 Virus S _ chymotrypsin_ full_309N 2021-4-11

Sequence = HKNNKSWMESEF; Mods = NGlycan/1971.7189/HexNAc(5)Hex(5)Fuc(1)

z=4

Error (ppm) = -0.59

Retention Time (min) = 20.14

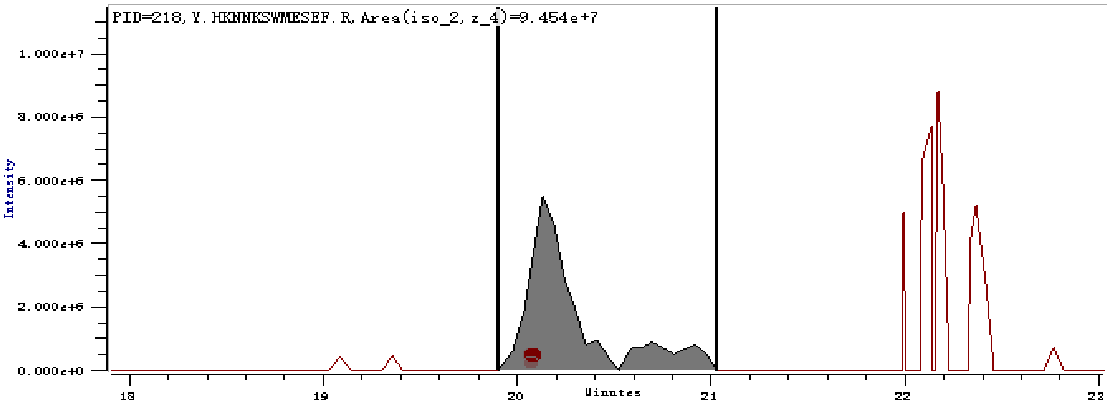

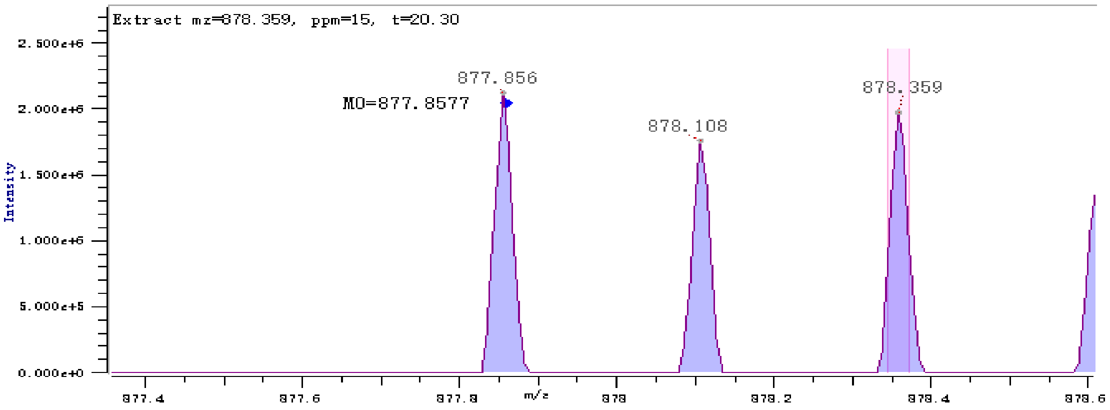

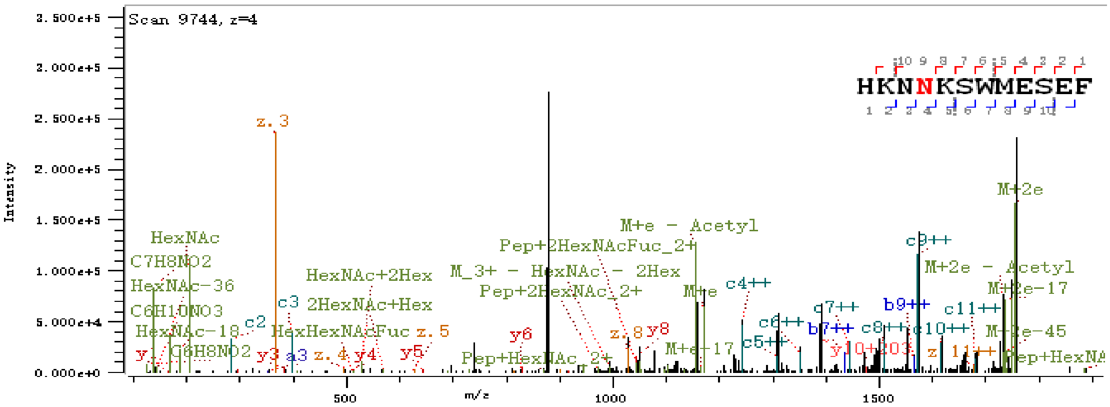

N165

Sample = SARS-COV-2 Virus S _ chymotrypsin_ full_309N 2021-4-11

Sequence = SSANNCTF; Mods = NGlycan/1971.7189/HexNAc(5)Hex(5)Fuc(1)

z=3

Error (ppm) = -0.70

Retention Time (min) = 18.48

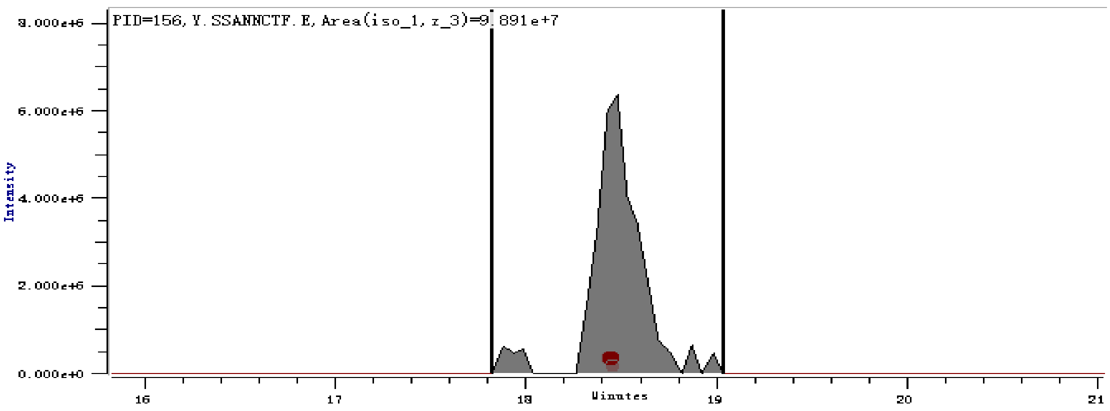

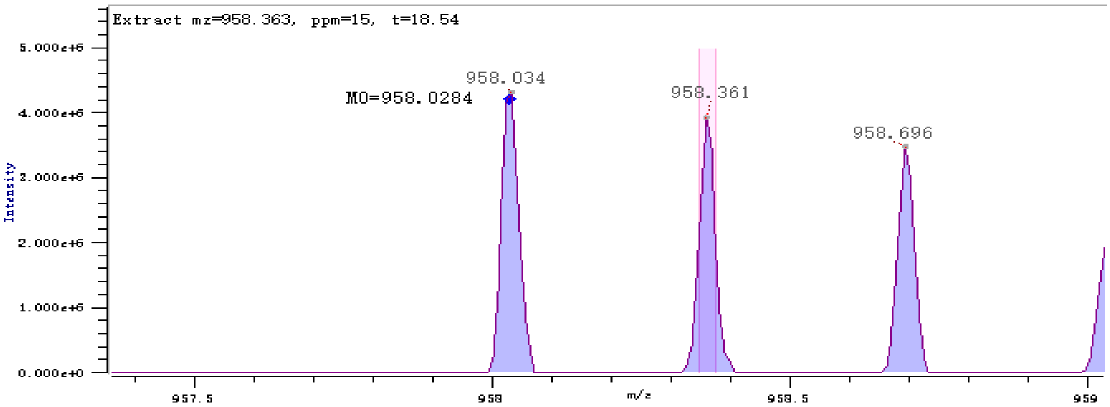

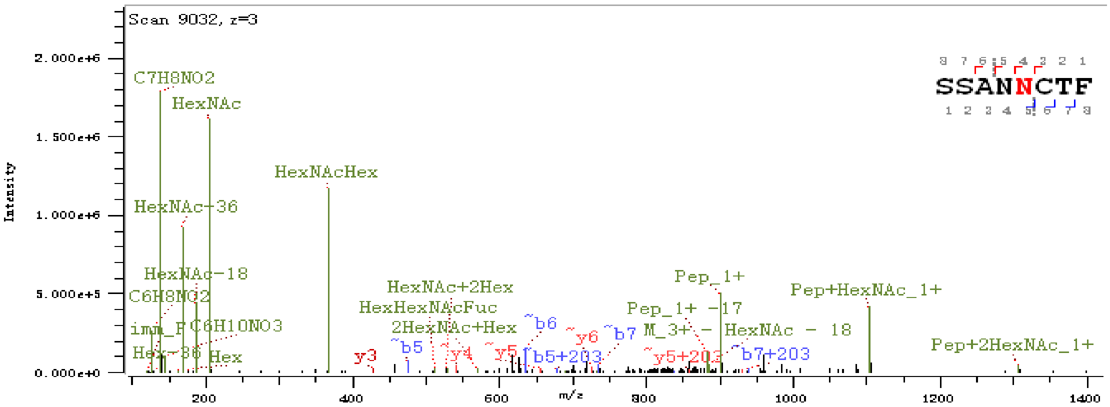

N234

Sample = SARS-COV-2 Virus S _ chymotrypsin_ full_309N 2021-4-11

Sequence = SALEPLVDLPIGINITRF; Mods = NGlycan/1864.6342/HexNAc(2)Hex(9)

z=3

Error (ppm) = 0.11

Retention Time (min) = 142.03

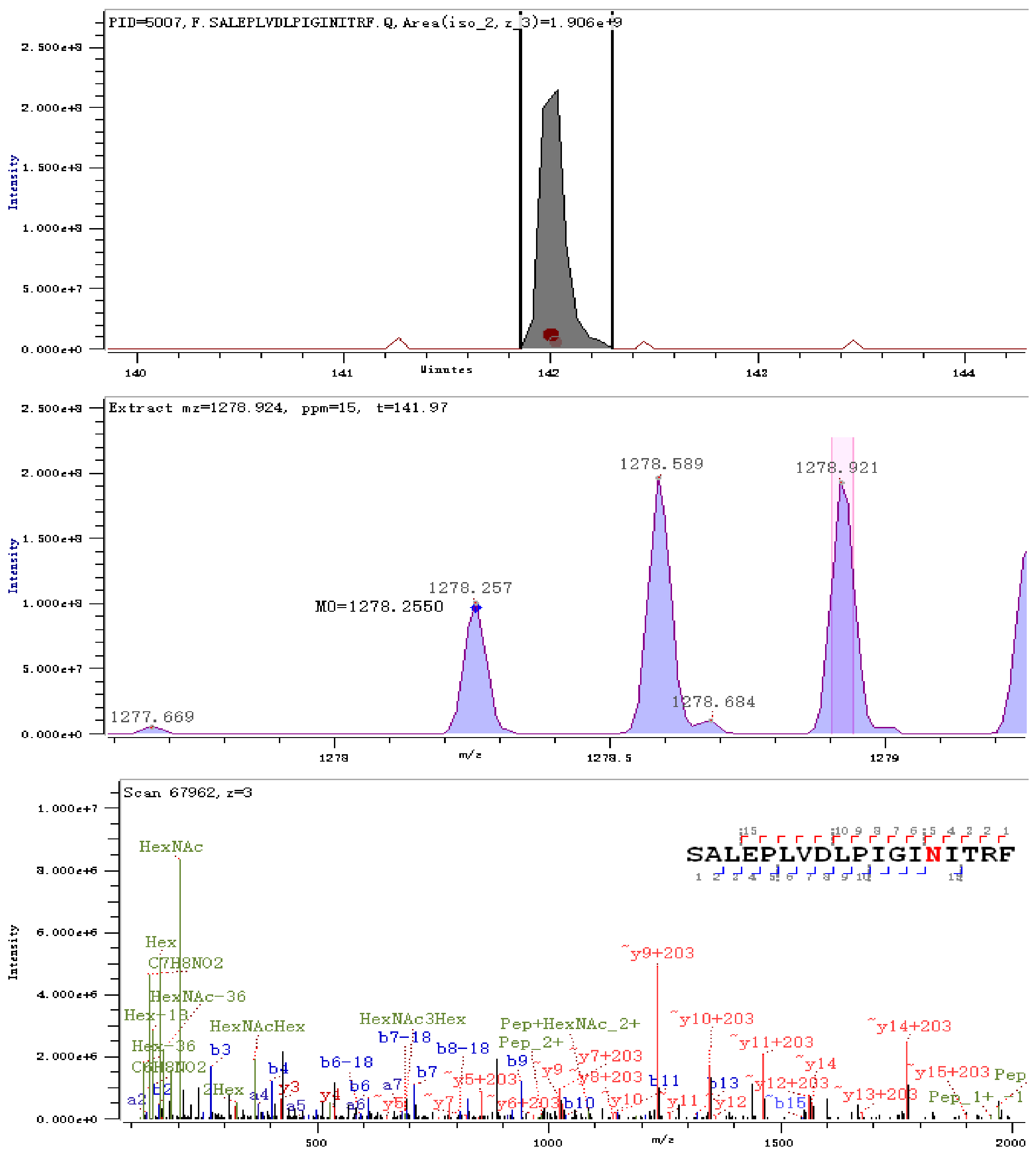

N282

Sample = SARS-COV-2 Virus S _trypsin_ full_309N 2021-4-11

Sequence = YNENGTITDAVDCALDPLSETK; Mods = NGlycan/1622.5816/HexNAc(4)Hex(5)

z=3

Error (ppm) = -0.01

Retention Time (min) = 126.59

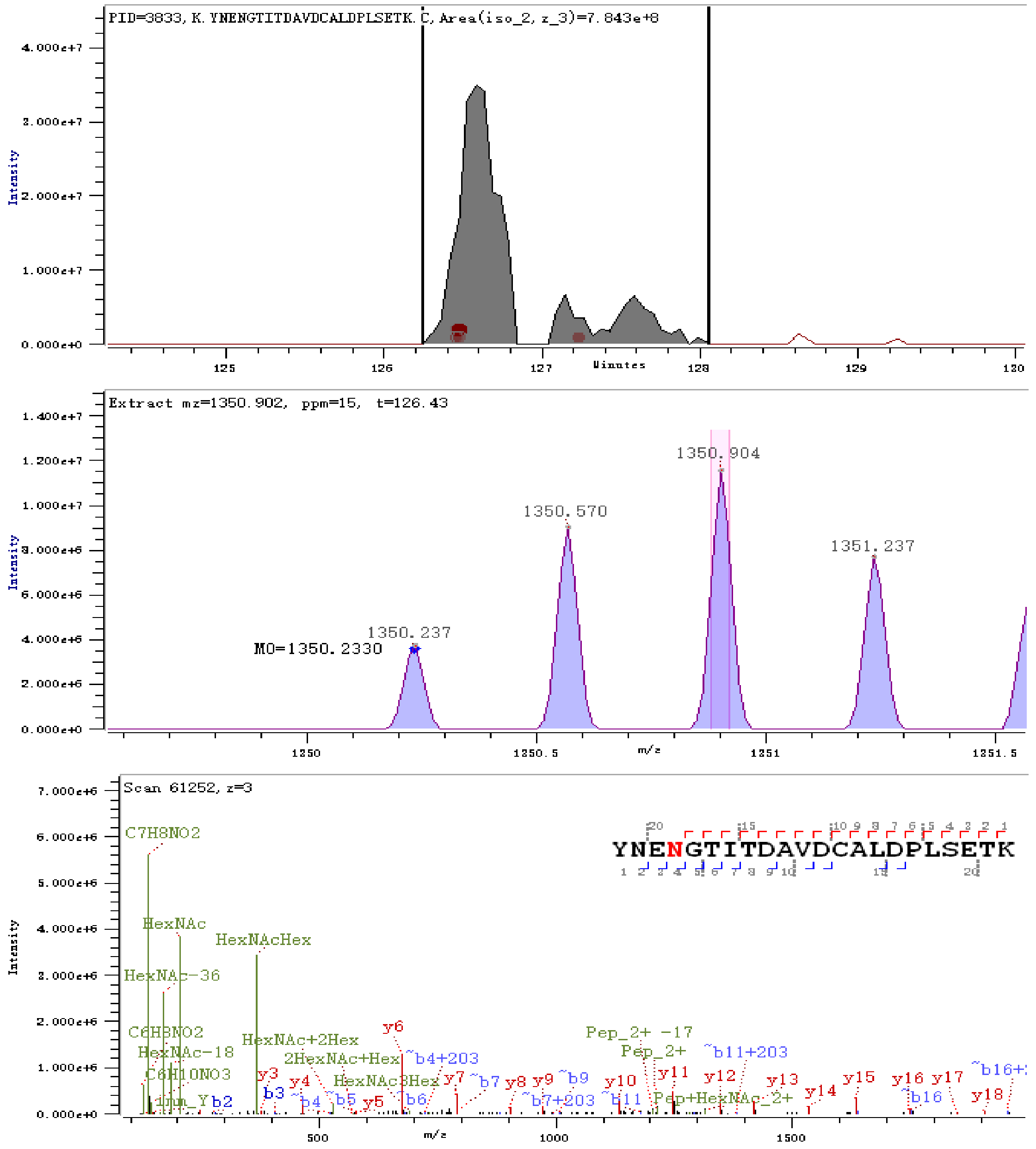

N331

Sample = SARS-COV-2 Virus S _ chymotrypsin_ full_309N 2021-4-11

Sequence = RVQPTESIVRFPNITNLCPF; Mods = NGlycan/1825.6610/HexNAc(5)Hex(5)

z=4

Error (ppm) = 1.10

Retention Time (min) = 128.48

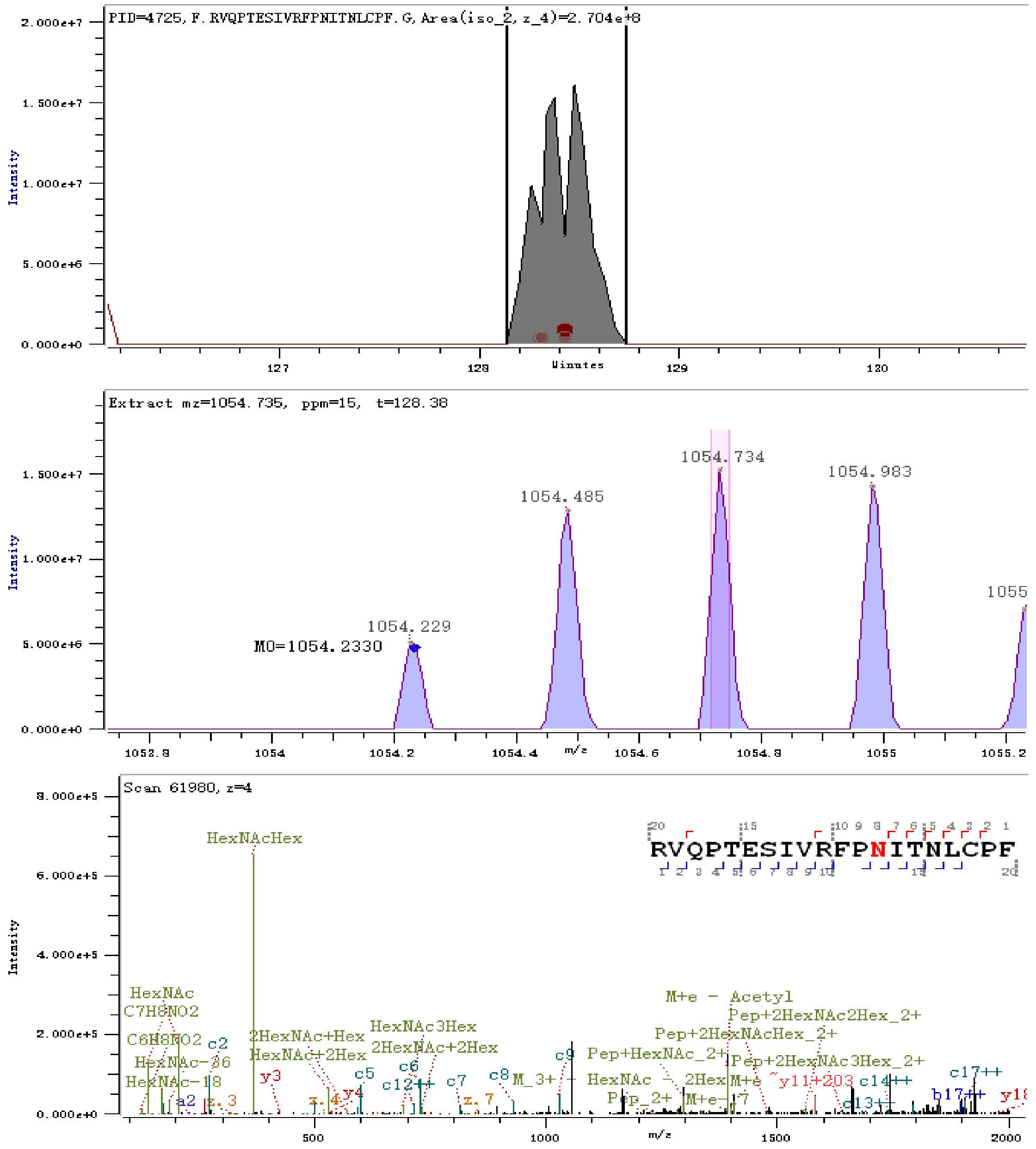

N343

Sample = SARS-COV-2 Virus S _ Lytic_ full_309N 2021-4-11

Sequence = NLCPFGEVFNAT; Mods = NGlycan/1216.4229/HexNAc(2)Hex(5)

z=2

Error (ppm) = 0.61

Retention Time (min) = 129.08

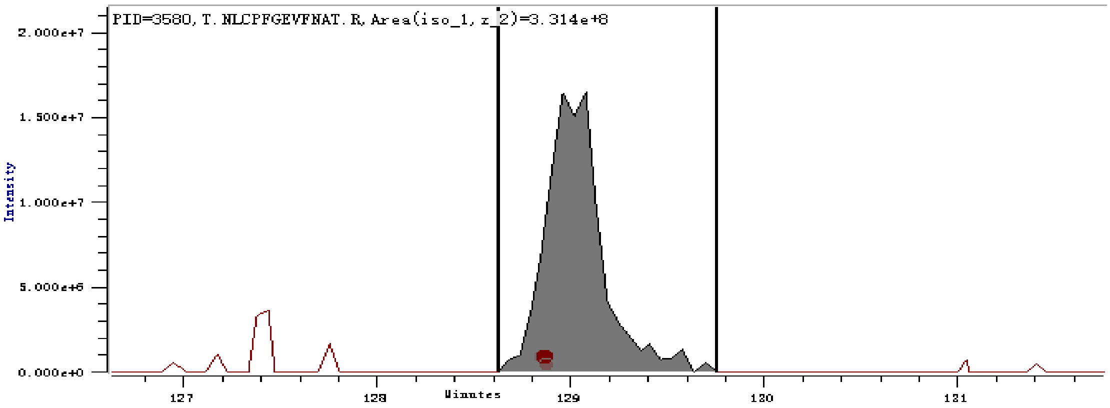

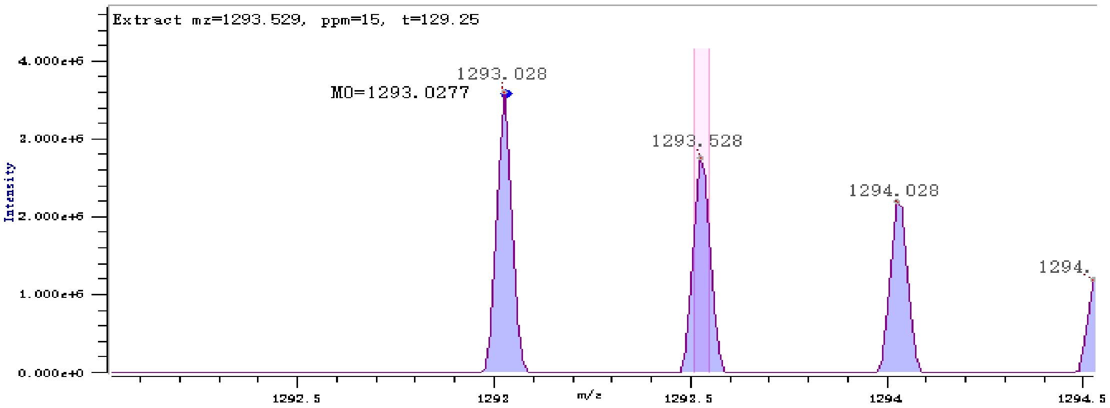

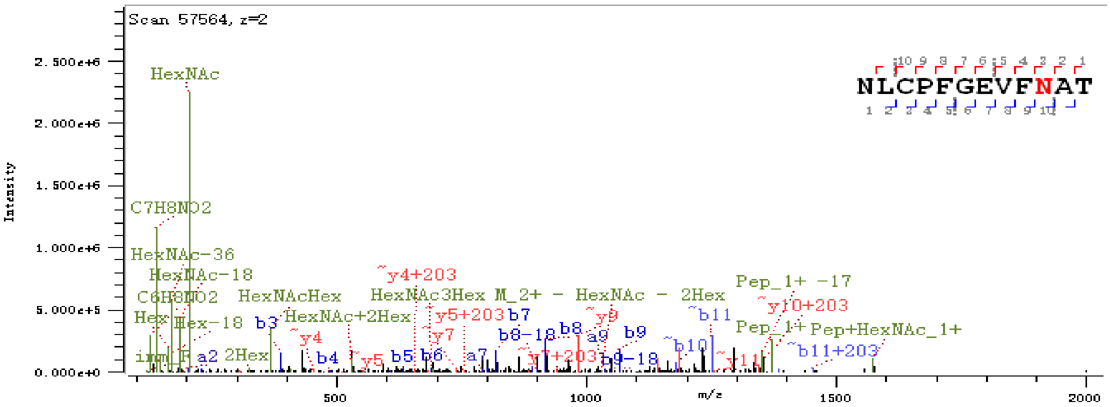

N603

Sample = SARS-COV-2 Virus S _ chymotrypsin_ full_309N 2021-4-11

Sequence = GGVSVITPGTNTSNQVAVLY; Mods = NGlycan/1581.5551/HexNAc(3)Hex(6)

z=3

Error (ppm) = -0.18

Retention Time (min) = 120.69

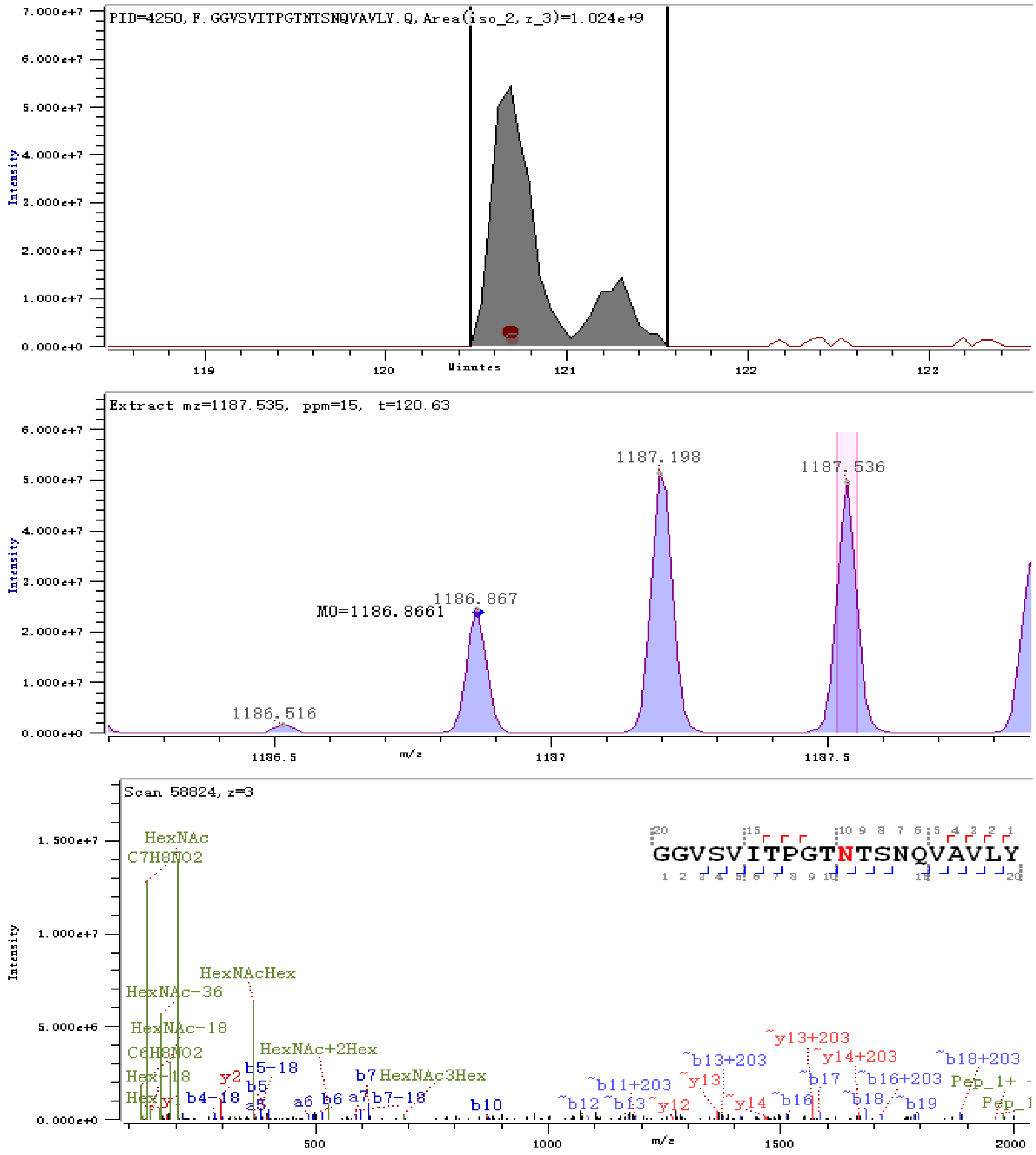

N616

Sample = SARS-COV-2 Virus S _ Lytic_ full_309N 2021-4-11

Sequence = LYQDVNCT; Mods = NGlycan/1419.5022/HexNAc(3)Hex(5)

z=2

Error (ppm) = -0.46

Retention Time (min) = 28.97

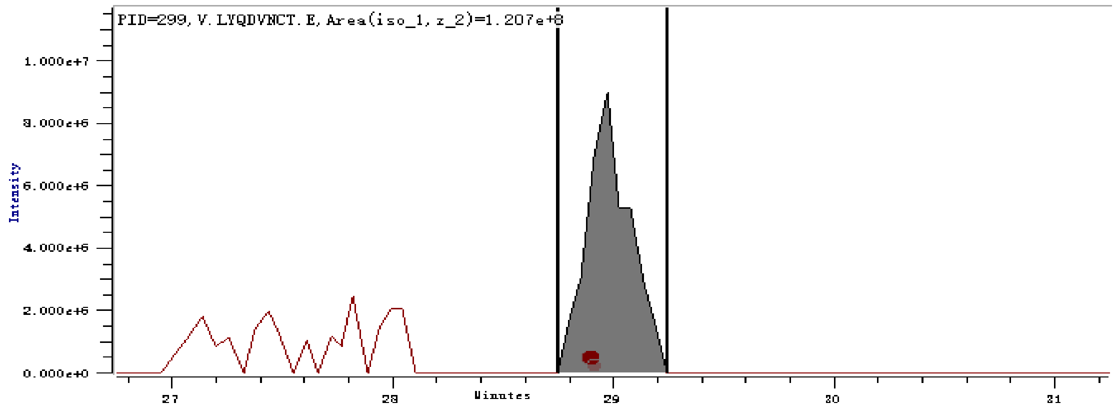

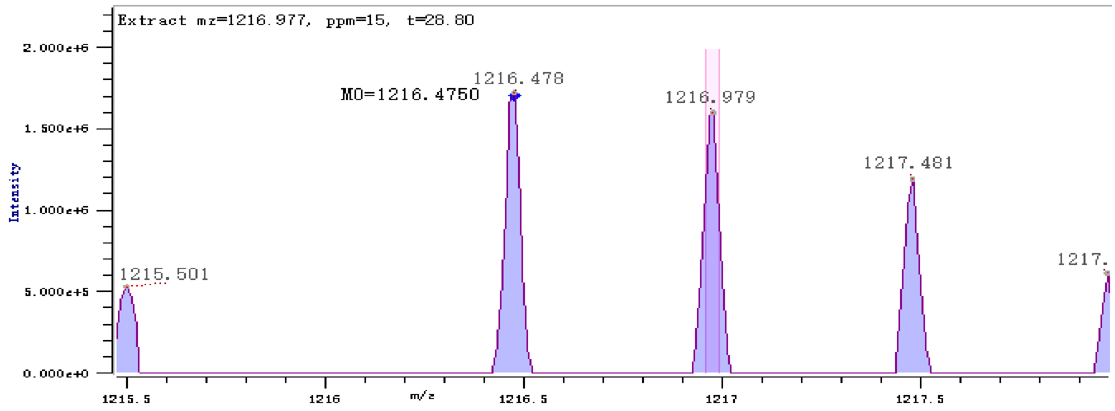

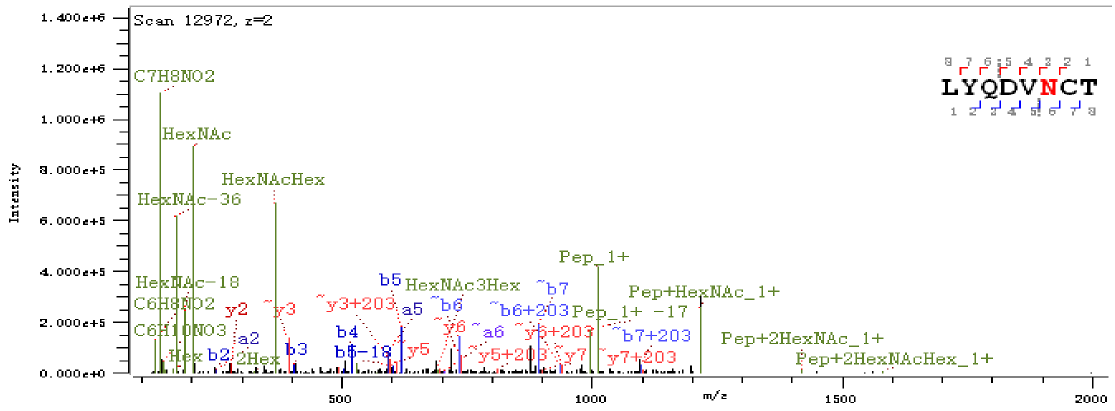

N657

Sample = SARS-COV-2 Virus S _ chymotrypsin_ full_309N 2021-4-11

Sequence = QTRAGCLIGAEHVNNSYECDIPIGAGICASY;

Mods = NGlycan/2174.7983/HexNAc(6)Hex(5)Fuc(1)

z=4

Error (ppm) = -1.19

Retention Time (min) = 110.62

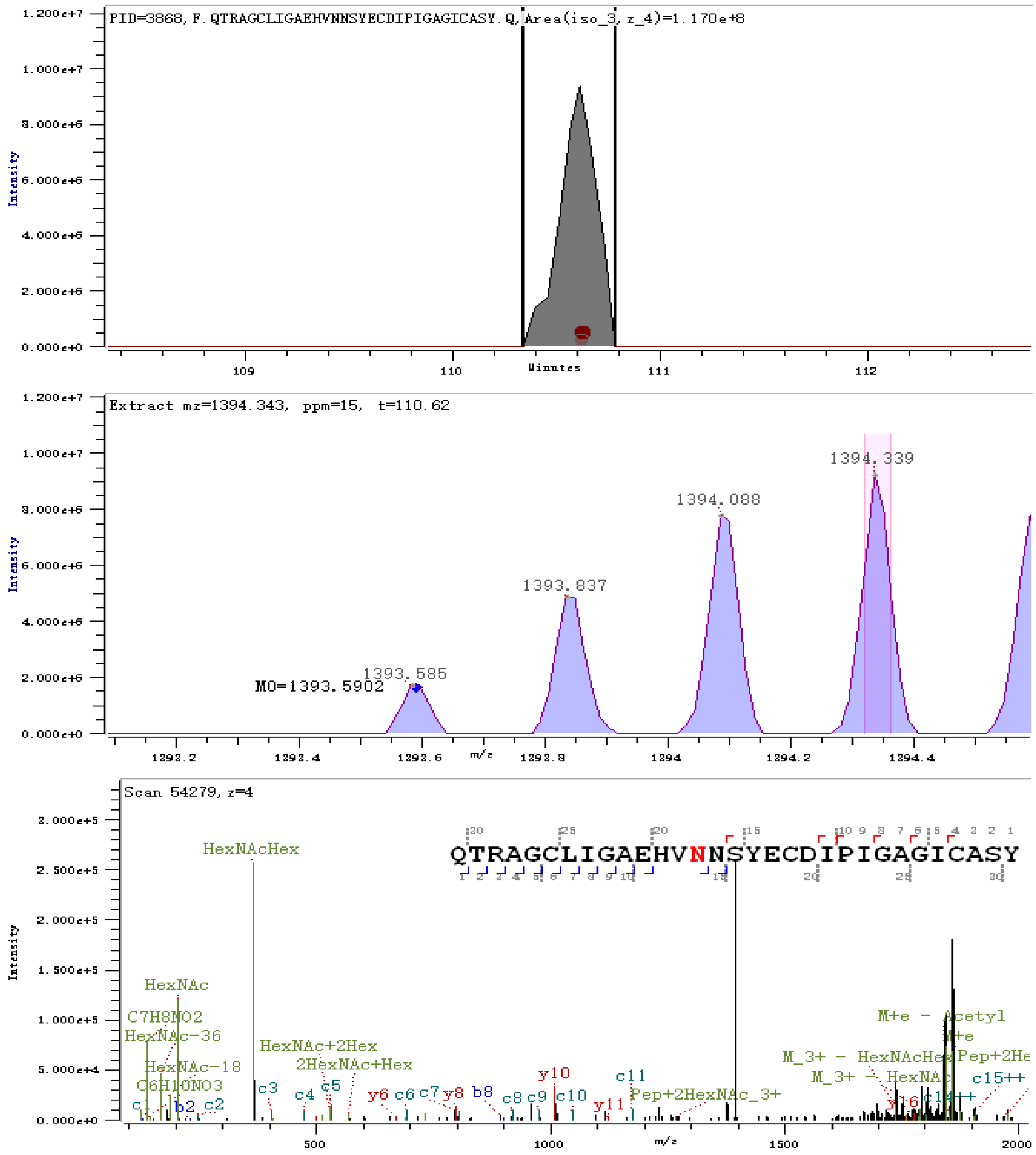

N709

Sample = SARS-COV-2 Virus S _ chymotrypsin_ full_309N 2021-4-11

Sequence = SNNSIAIPTNF; Mods = NGlycan/3147.1152/HexNAc(6)Hex(11)Fuc(1)

z=3

Error (ppm) = 1.89

Retention Time (min) = 90.54

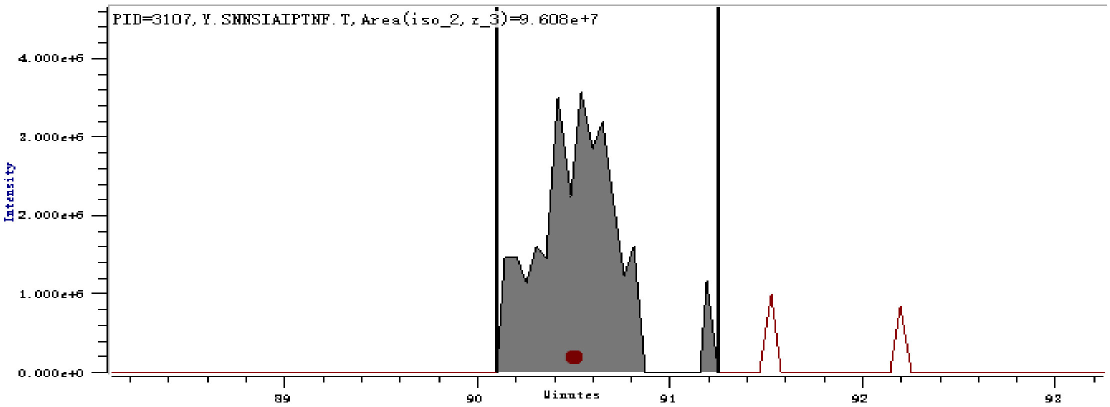

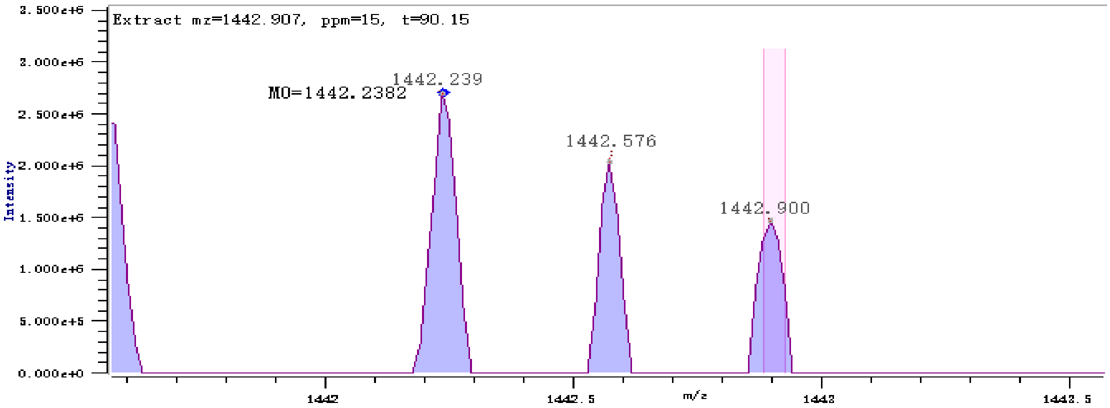

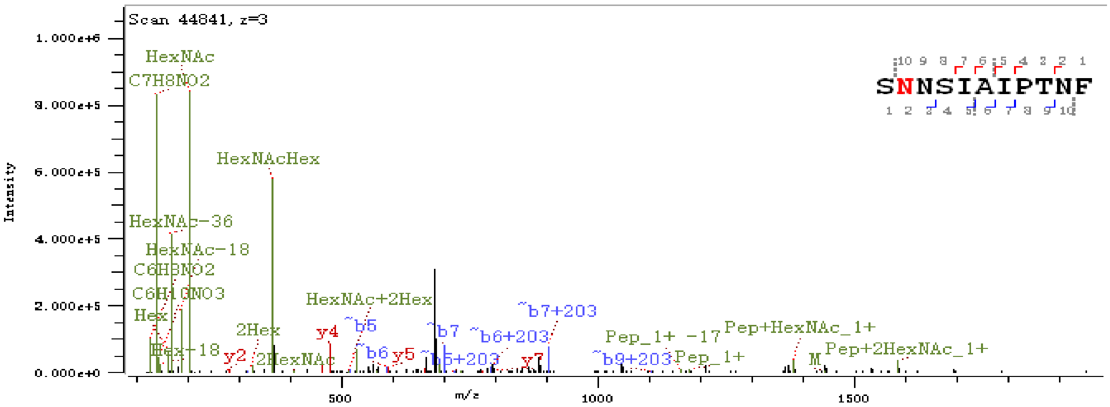

N717

Sample = SARS-COV-2 Virus S _ Lytic_ full_309N 2021-4-11

Sequence = NFTISV; Mods = NGlycan/1540.5285/HexNAc(2)Hex(7)

z=2

Error (ppm) = 0.62

Retention Time (min) = 77.54

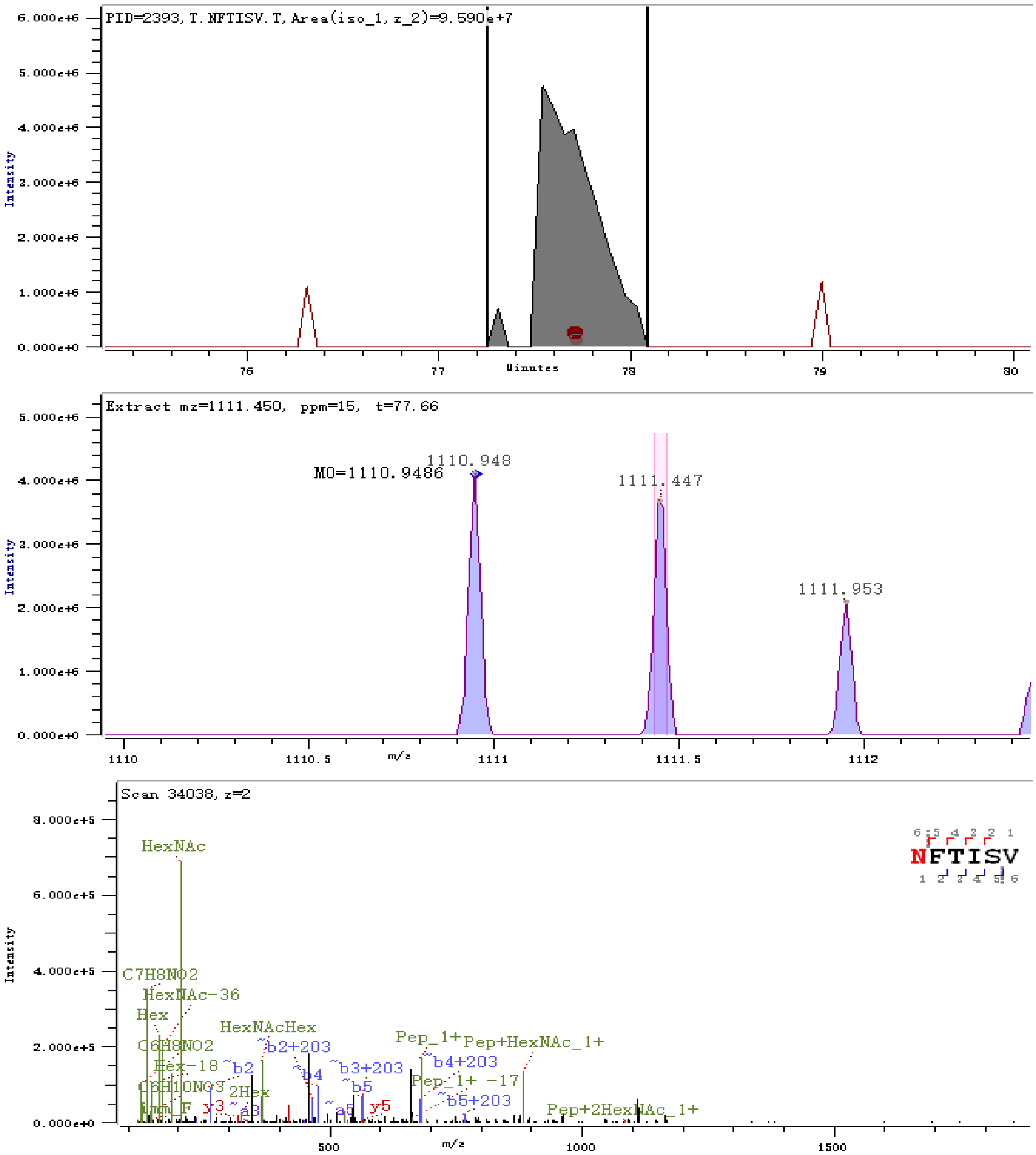

N801

Sample = SARS-COV-2 Virus S _ Lytic_ full_309N 2021-4-11

Sequence = KQIYKTPPIKDFGGFNFSQILPDPSKPS; Mods = NGlycan/1702.5813/HexNAc(2)Hex(8)

z=5

Error (ppm) = 0.28

Retention Time (min) = 119.73

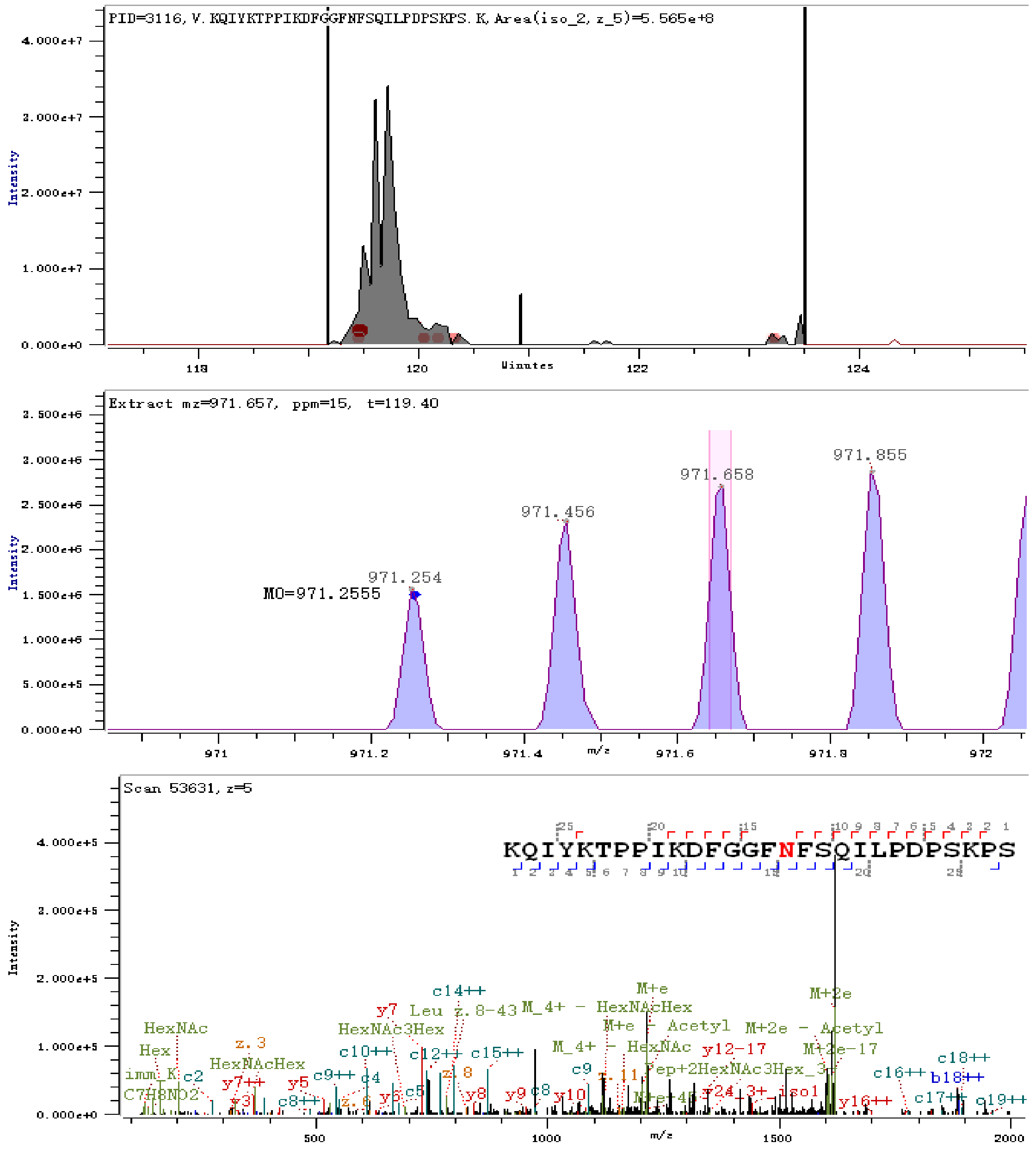

N1074

Sample = SARS-COV-2 Virus S _trypsin_ full_309N 2021-4-11

Sequence = NFTTAPAICHDGK; Mods = NGlycan/1540.5285/HexNAc(2)Hex(7)

z=3

Error (ppm) = 0.71

Retention Time (min) = 20.33

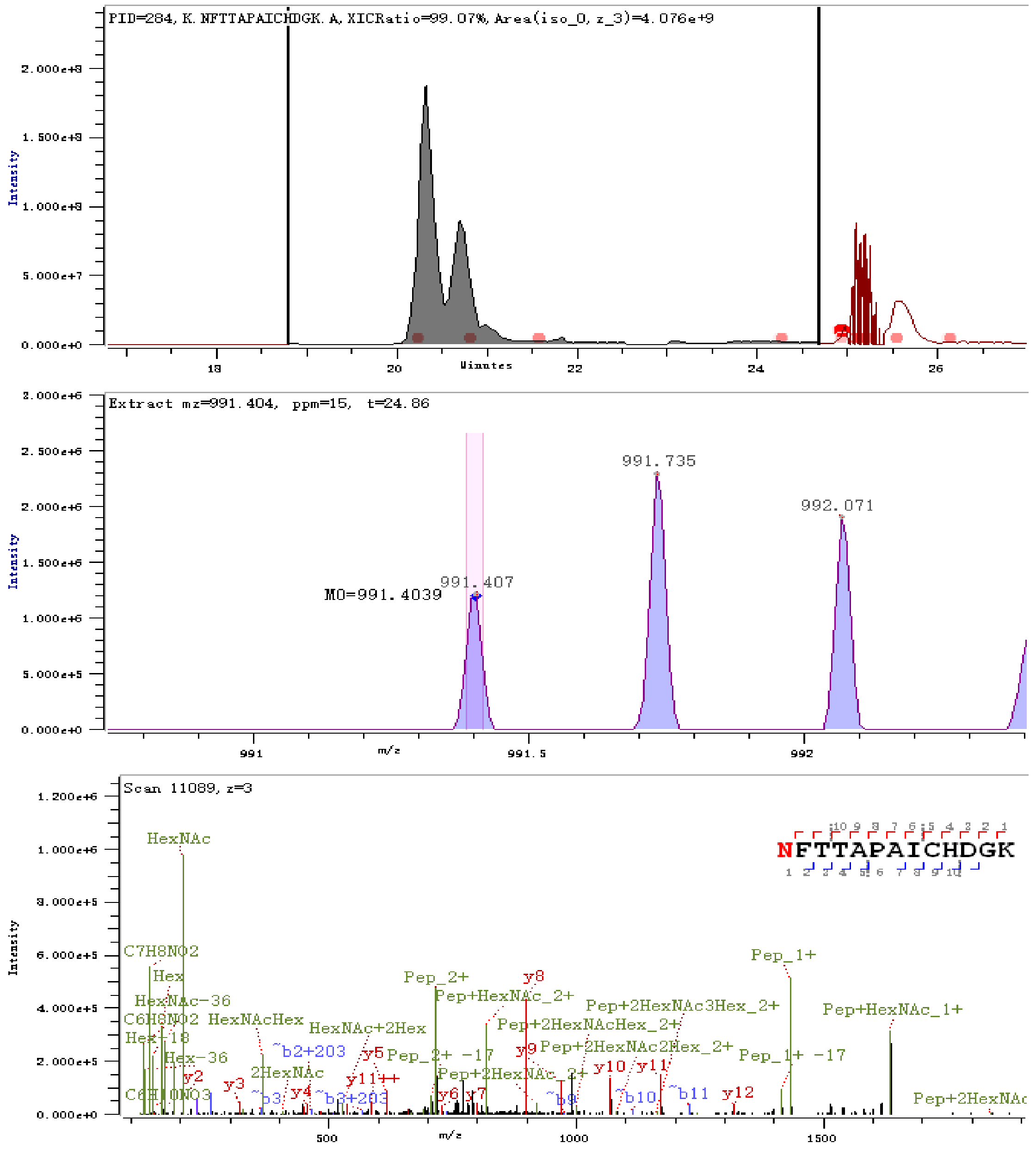

N1098

Sample = SARS-COV-2 Virus S _trypsin_ full_309N 2021-4-11

Sequence = EGVFVSNGTHWFVTQR; Mods = NGlycan/2190.7932/HexNAc(6)Hex(6)

z=3

Error (ppm) = 2.49

Retention Time (min) = 82.28

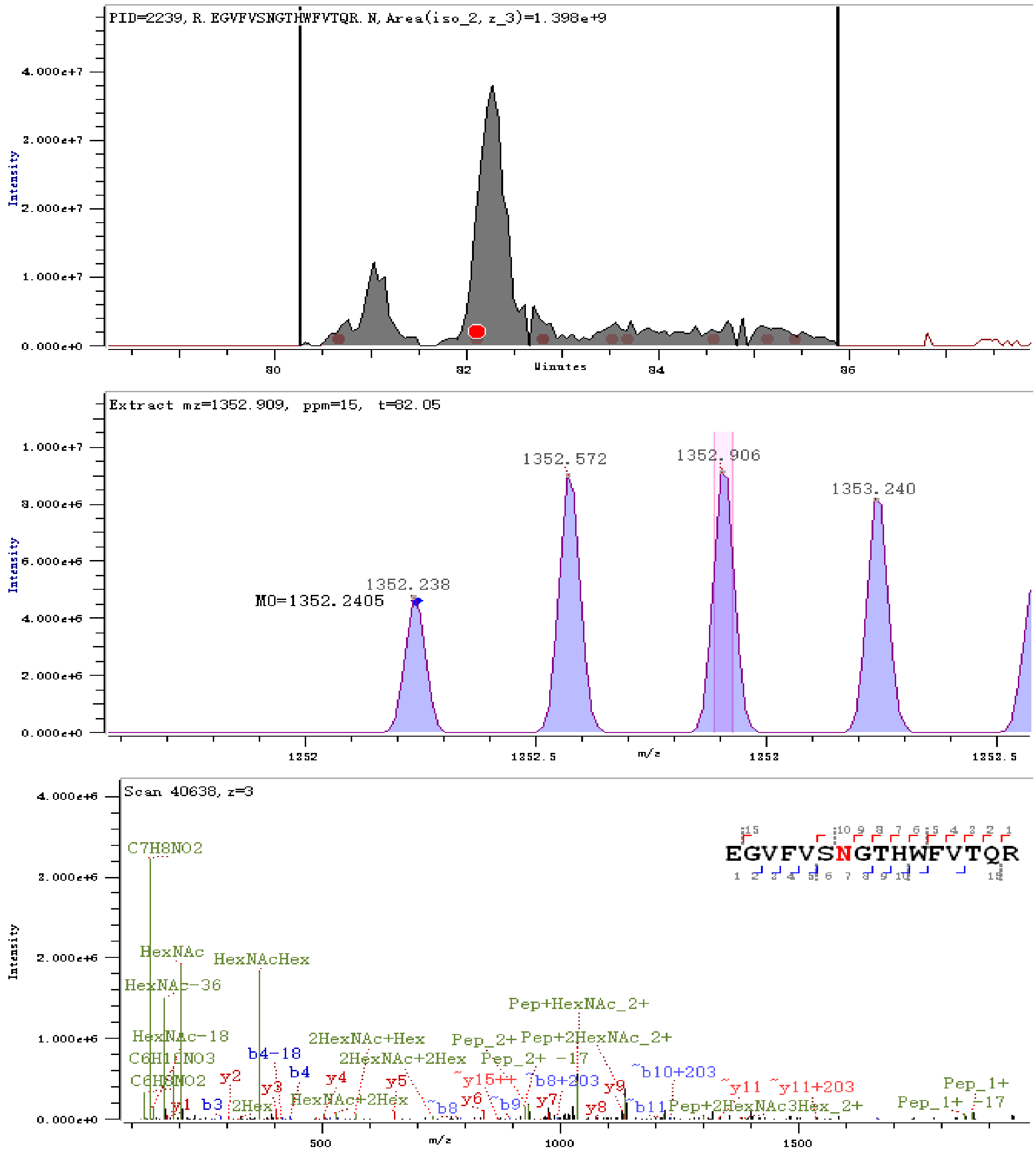

N1134

Sample = SARS-COV-2 Virus S _ Lytic_ full_309N 2021-4-11

Sequence = IGIVNNT; Mods = NGlycan/2424.8671/HexNAc(5)Hex(6)Fuc(1)NeuAc(1)

z=3

Error (ppm) = -0.37

Retention Time (min) = 69.27

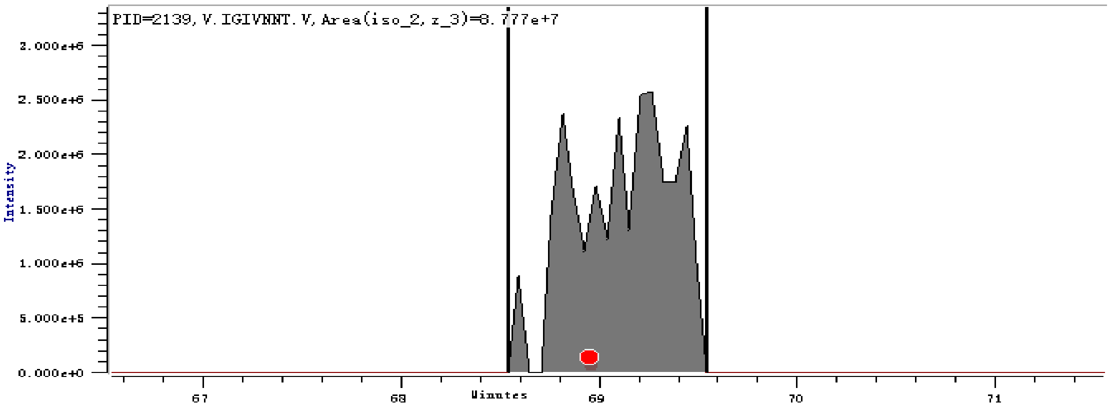

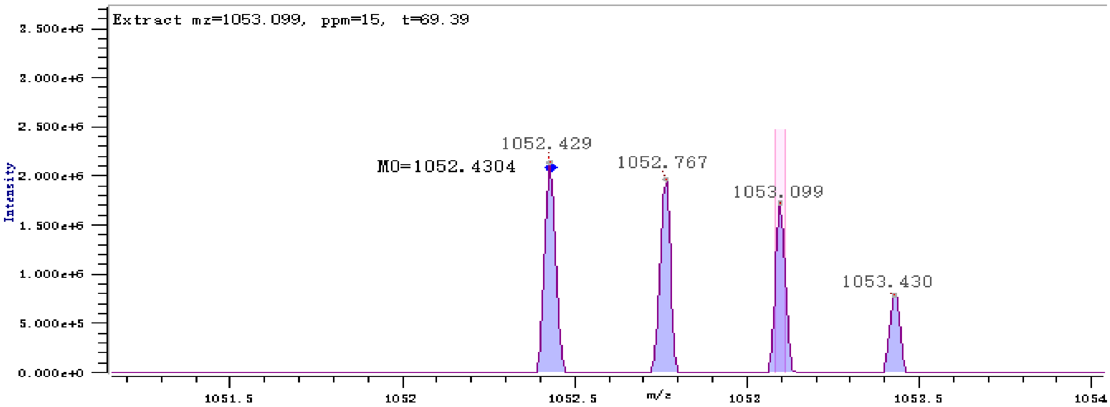

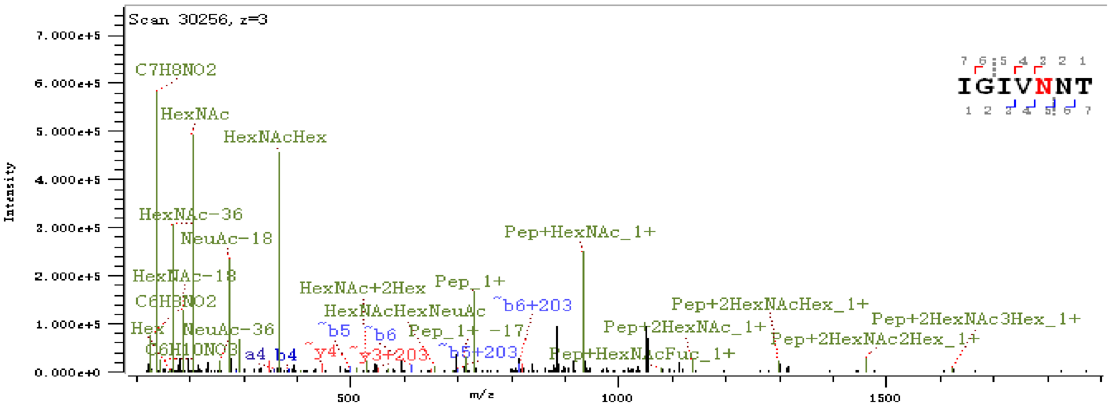

N1158

Sample = SARS-COV-2 Virus S _deglycan_ O18_ chymotrypsin_ semi_78O 2021-4-11

Sequence = FKNHTSPDVDLGD; Mods = Deamidated/2.9883/

z=2

Error (ppm) = -0.80

Retention Time (min) = 39.86

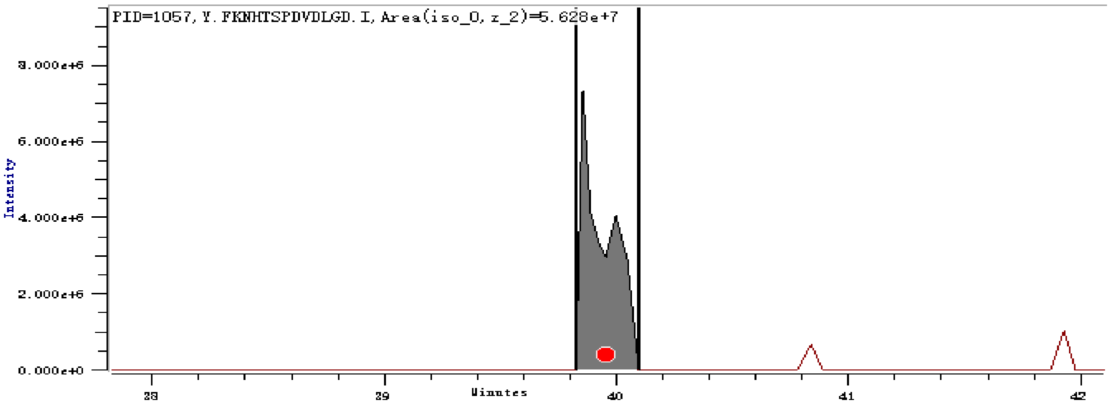

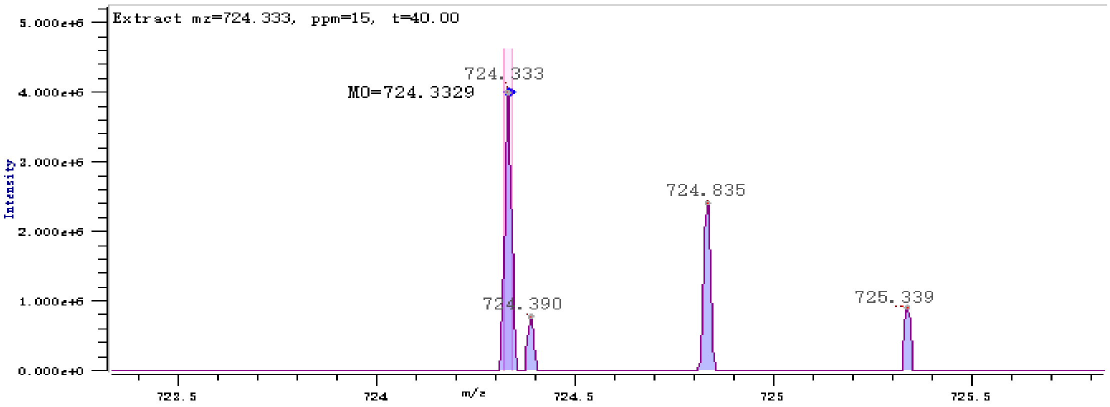

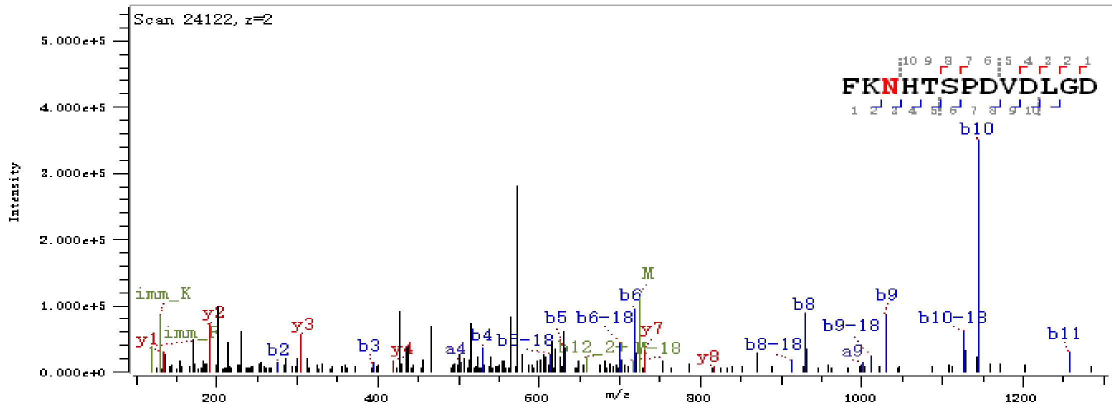

N1173

Sample = SARS-COV-2 Virus S _deglycan_ O18_ Lytic_semi_78O 2021-4-11

Sequence = DISGINAS; Mods = Deamidated/2.9883/

z=1

Error (ppm) = -0.91

Retention Time (min) = 63.40

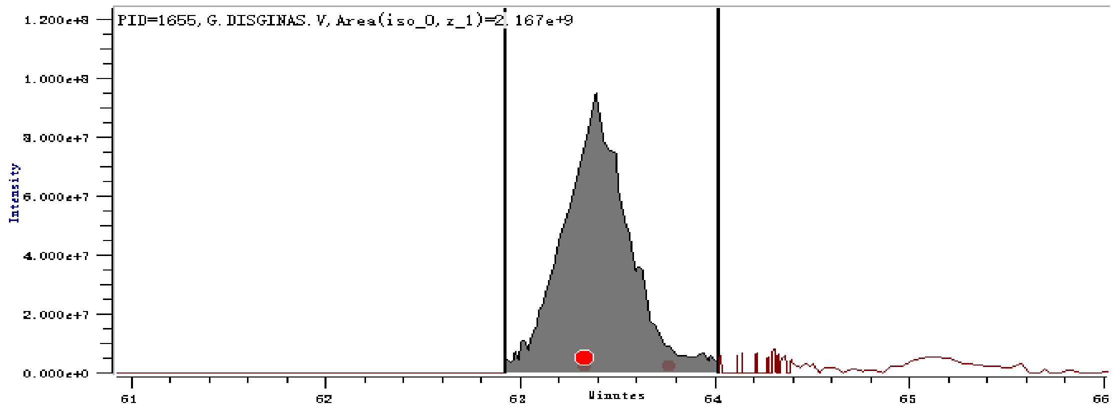

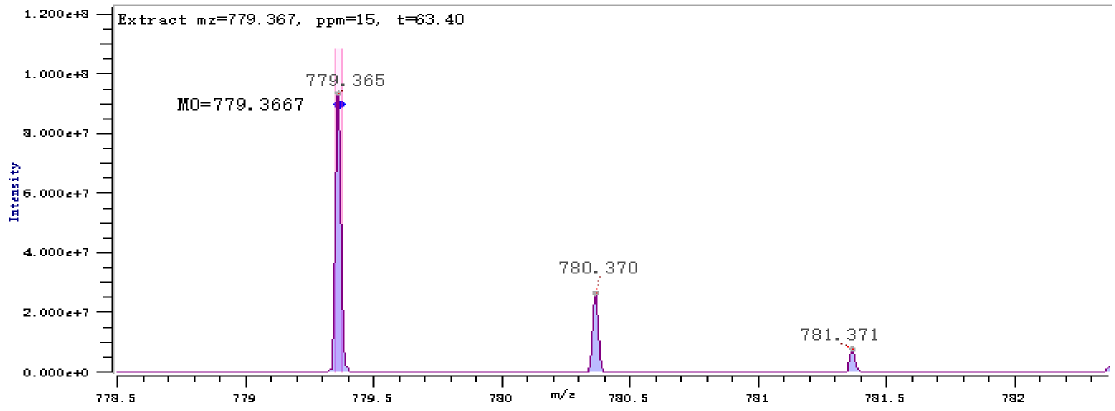

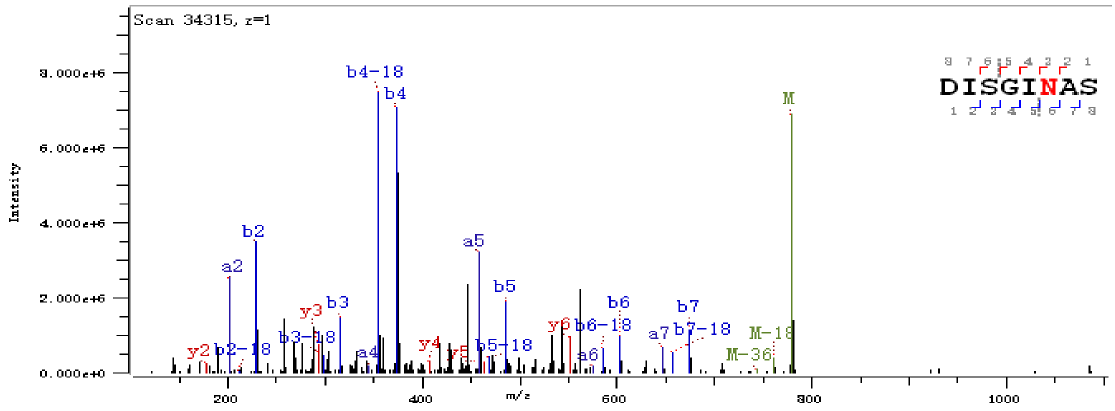

N1194

Sample = SARS-COV-2 Virus S _trypsin_ full_309N 2021-4-11

Sequence = NLNESLIDLQELGK; Mods = NGlycan/1768.6395/HexNAc(4)Hex(5)Fuc(1)

z=3

Error (ppm) = 1.10

Retention Time (min) = 113.16

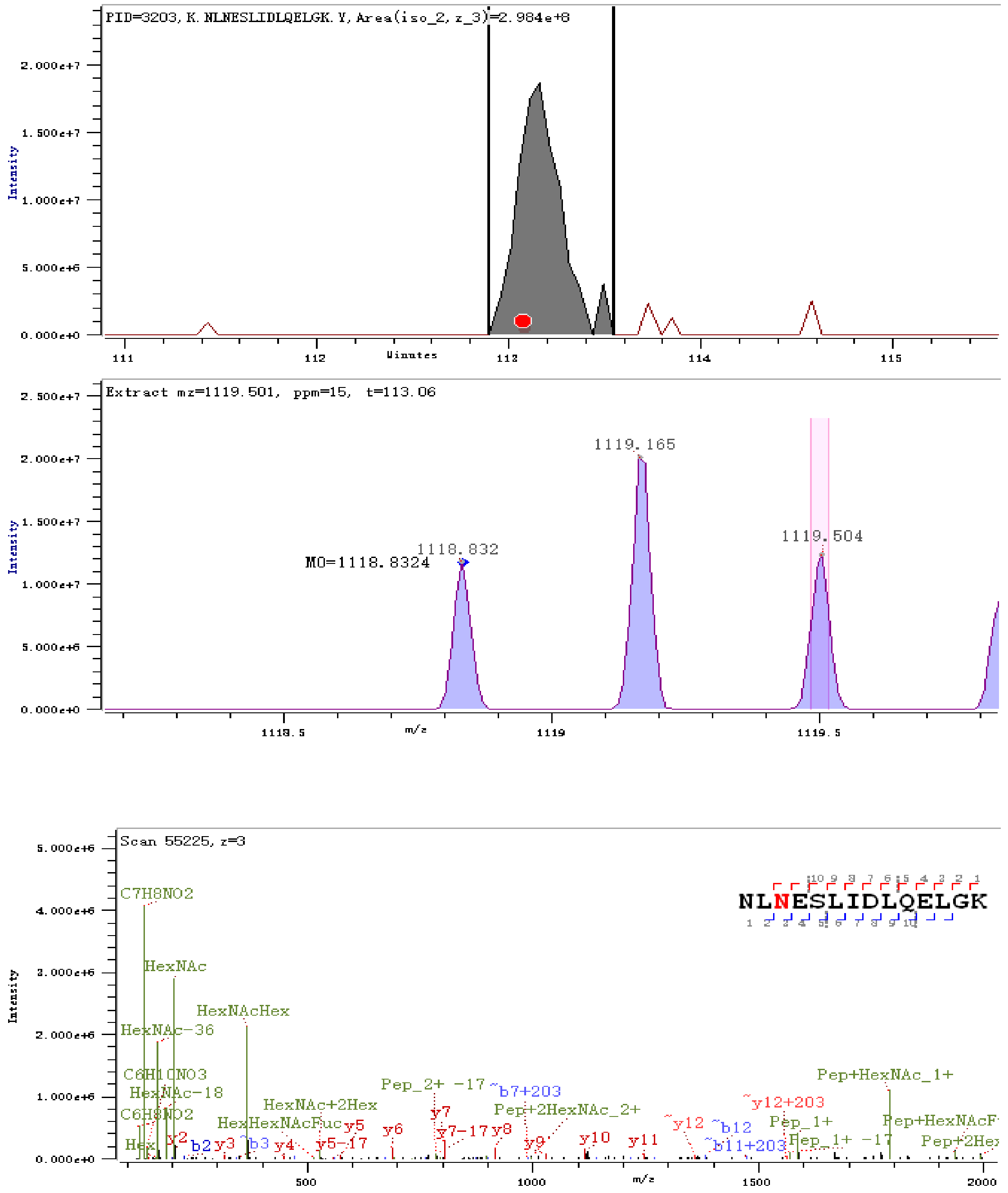

Fig. S2. The identification of 22 *N*-glycosites of the S protein extracted from the SARS-CoV-2 virions

For each peptide/glycopeptide detected the corresponding extracted ion chromatogram (XIC) (upper panel), precursor isotope distribution (middle panel), MS/MS spectrum (lower panel) are shown. The monoisotopic peak is marked as “MO=” with a blue dot (middle panel). The fragment b- and

y-ions are in blue and red color, respectively; the c- and z-ions are marked in cyan and orange, respectively. The oxonium ions are in green color.

T22

Sample = 4th virus chymotrypsin HCD20 N+O using 6 most O 2021-6-29

Sequence = RTQLPPAYTNSFTRGVY; Mods = OGlycan/203.0794/HexNAc(1)

z=3

Error (ppm) = 1.51

Retention Time (min) = 61.99

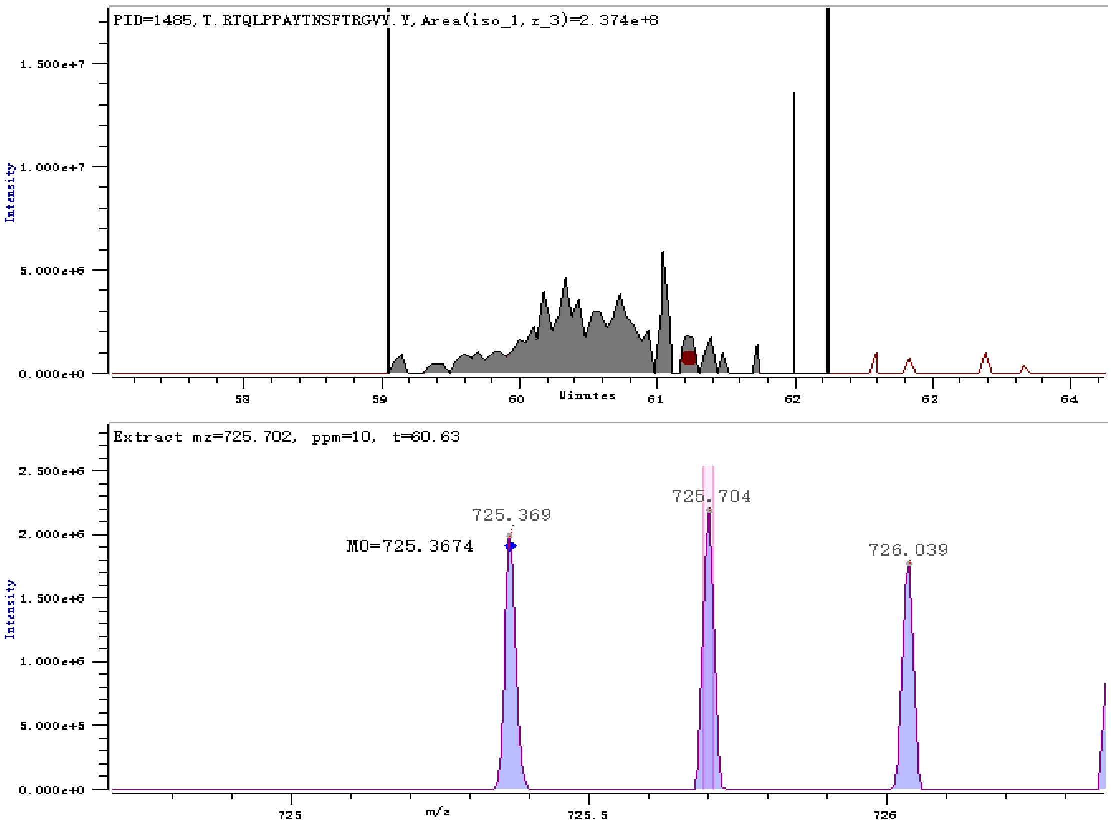

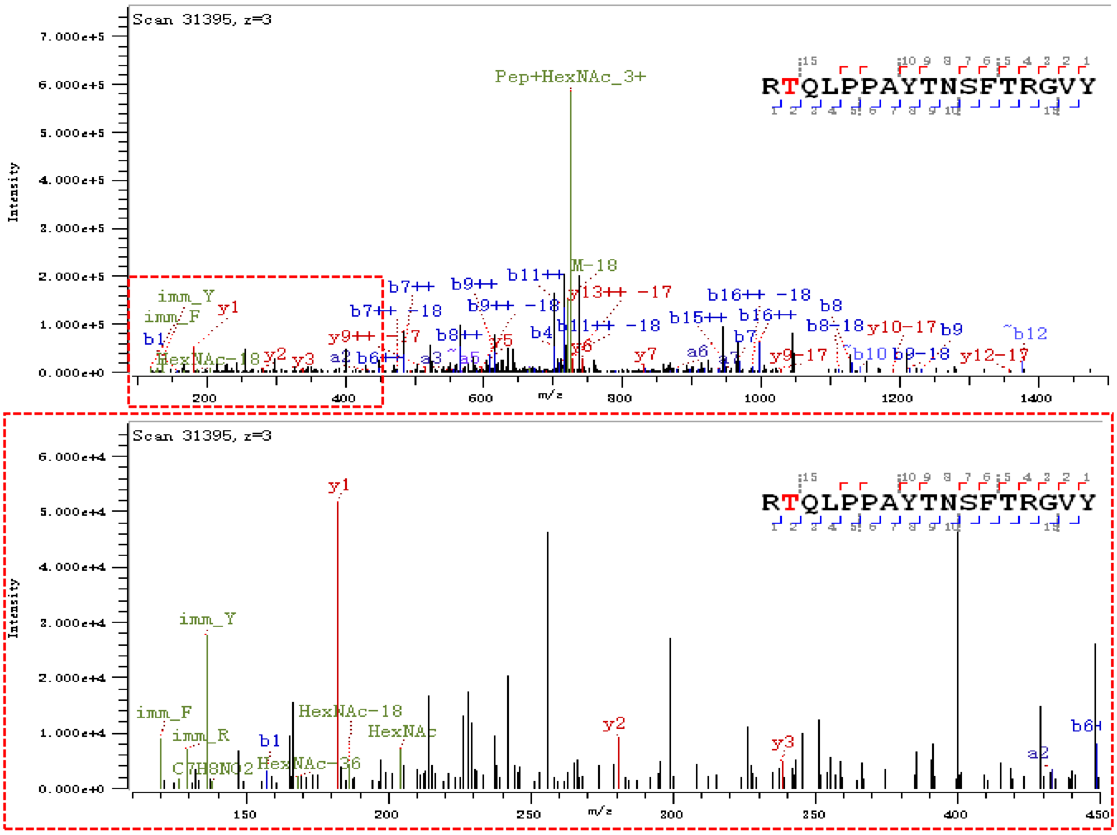

Diagnostic ions	observed m/z	B ions	observed m/z	Y ions	observed m/z
b1	157.097	C6H8NO2	126.056	M-36	713.363
a2	433.215	C7H8NO2	138.053	M-18	719.368
		HexNAc-36	168.066	Pep+HexNAc_3+	725.386
		HexNAc-18	186.077		
		HexNAc	204.087		

S60

Sample = 4th virus chymotrypsin HCD30 N+O using 6 most O 2021-6-29

Sequence = FSNVT;

Mods = NGlycan/1378.4757; OGlycan/365.1322/HexNAc(2)Hex(6),HexNAc(1)Hex(1)

z=2

Error (ppm) = 5.69

Retention Time (min) = 85.20

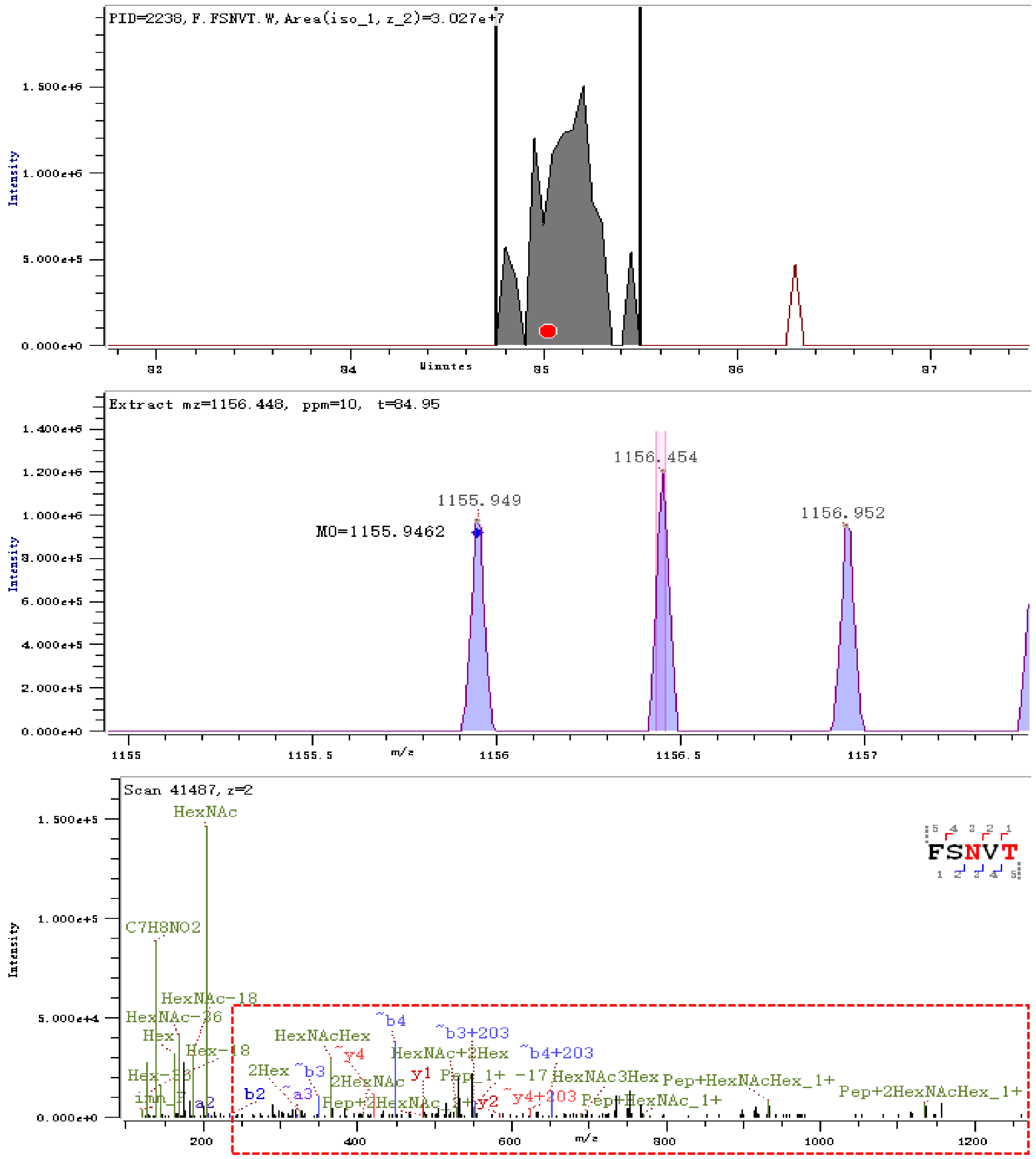

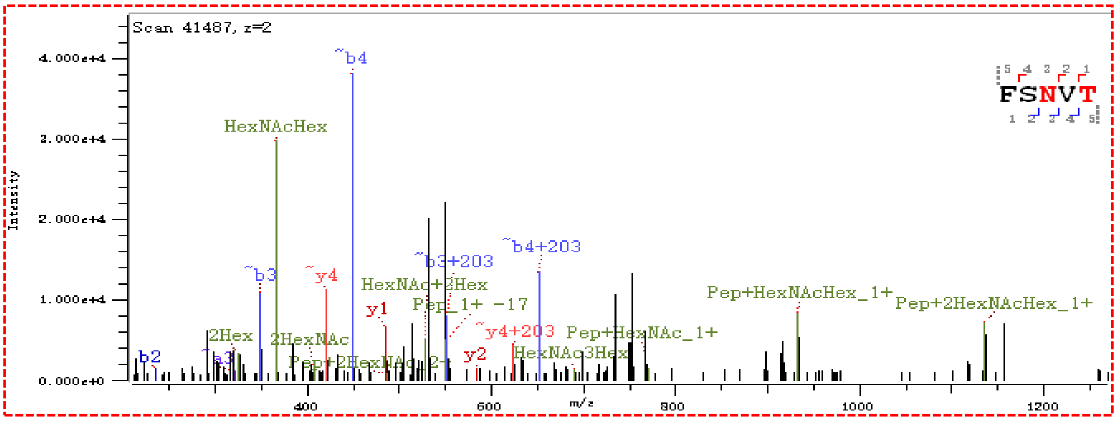

Diagnostic ions	observed m/z	B ions	observed m/z	Y ions	observed m/z
~b3	349.152	C6H8NO2	126.055	Pep_1+ -17	550.270
~b3+203	552.23	C7H8NO2	138.055	Pep+HexNAc_1+	700.367
~b4	448.220	C6H10NO3	144.066	Pep+2HexNAc_2+	487.220
~b4+203	651.300	Hex-36	127.039	Pep+HexNAcHex_1+	932.415
y1	485.204	HexNAc-36	168.066	Pep+2HexNAcHex_1+	1135.494
y2	584.271	HexNAc-18	186.076		
		HexNAc	204.087		
		HexNAcHex	366.139		

T124

Sample = 4th virus chymotrypsin HCD15 N+O using 6 most O 2021-6-29

Sequence = IVNNATNVVIKVCEF;

Mods = NGlycan/1736.6497; OGlycan/203.0794/HexNAc(4)Hex(3)Fuc(3),HexNAc(1)

z=3

Error (ppm) = 8.96

Retention Time (min) = 96.70

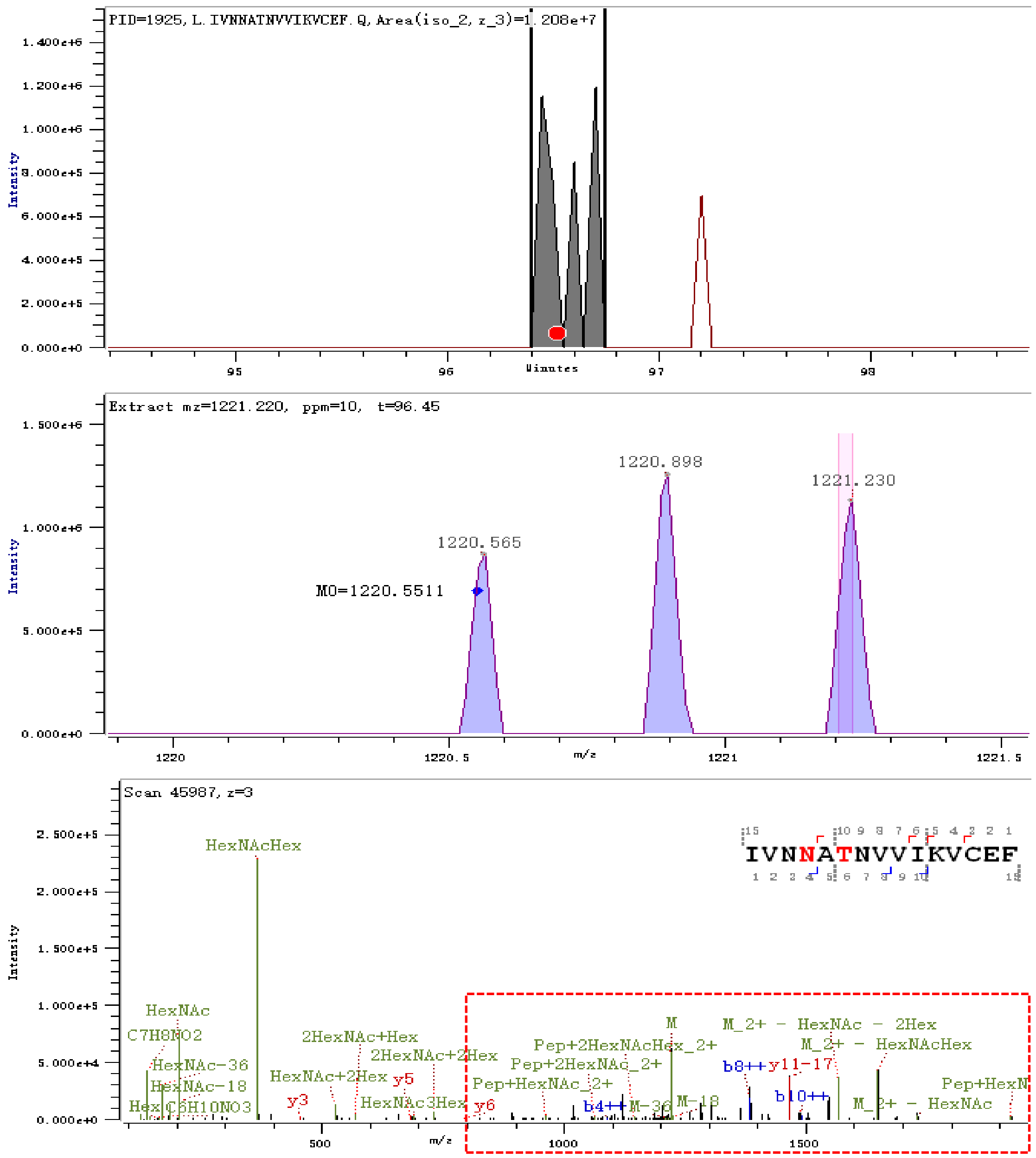

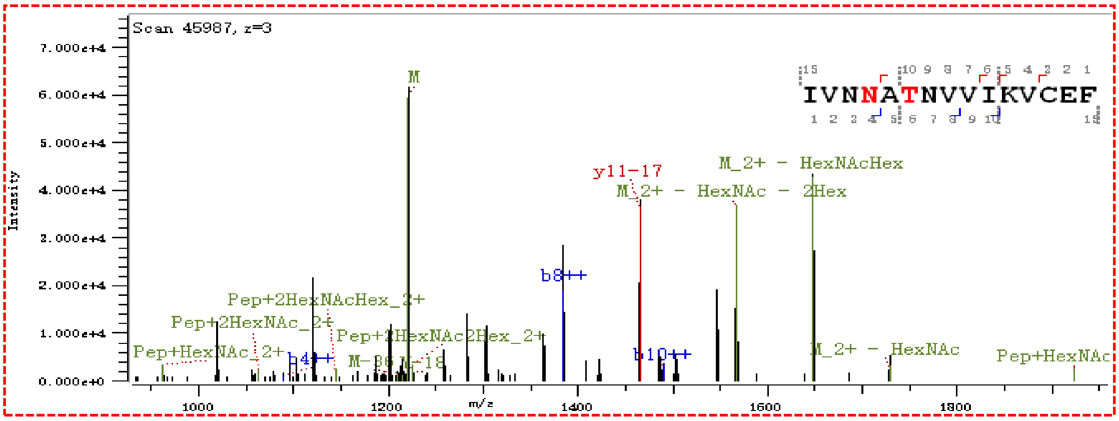

Diagnostic ions	observed m/z	B ions	observed m/z	Y ions	observed m/z
b4++	1089.523	C6H8NO2	126.055	M-36	1208.587
y11-17	1465.701	C7H8NO2	138.055	M-18	1214.562
		C6H10NO3	144.066	M	1220.594
		Hex	163.060	M_2+ - HexNAc2Hex	1566.740
		HexNAc-36	168.066	M_2+ - HexNAcHex	1647.766
		HexNAc-18	186.076	M_2+ - HexNAc	1728.789
		HexNAc	204.087	Pep+HexNAc_1+	1922.987
		HexNAcHex	366.140	Pep+HexNAc_2+	961.996
		HexNAc2Hex	528.194	Pep+2HexNAc_2+	1063.538
		HexNAc3Hex	690.246	Pep+2HexNAcHex_2+	1144.560
		2HexNAcHex	569.220	Pep+2HexNAc2Hex_2+	1225.583
		2HexNAc2Hex	731.272		

Sample = 2nd virus chymotrypsin N+O search

Sequence = YHKNNKSW;

Mods = NGlycan/2272.8463; OGlycan/365.1322/HexNAc(8)Hex(4), HexNAc(1)Hex(1)

z=4

Error (ppm) = -7.56

Retention Time (min) = 13.98

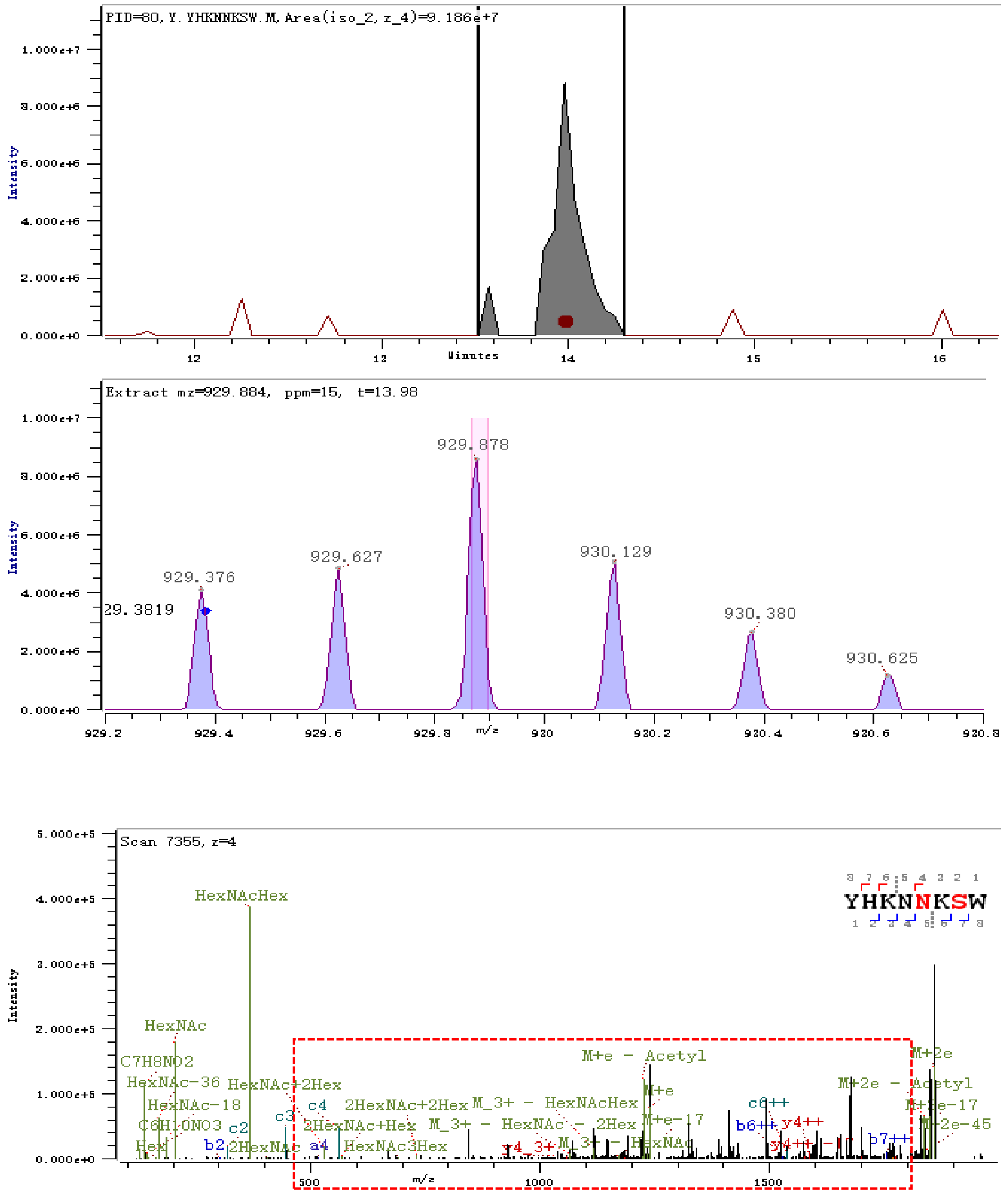

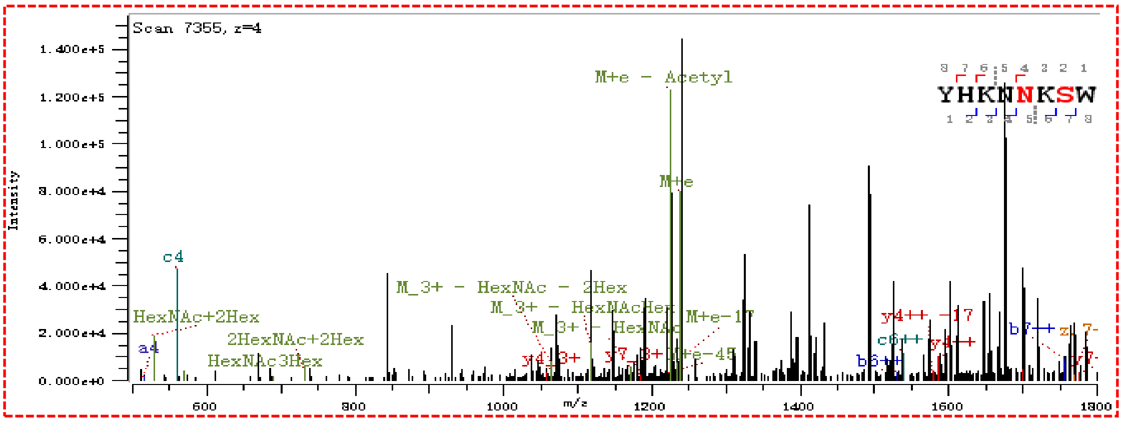

Diagnostic ions	observed m/z	B ions	observed m/z	Y ions	observed m/z
b6++	1529.627	C6H8NO2	126.055	M	924.414
b7++	1755.707	C7H8NO2	138.055	M_3+ - HexNAc2Hex	1063.102
c6++	1538.134	C6H10NO3	144.066	M_3+ - HexNAcHex	1117.121
c7++	1764.216	Hex-36	127.039	M_3+ - HexNAc	1171.146
		Hex-18	145.050	Pep+HexNAc_3+	427.184
		Hex	163.060	Pep+2HexNAcHex_1+	1644.668
		HexNAc-36	168.066	Pep+2HexNAc2Hex_1+	1806.739
		HexNAc-18	186.076		
		HexNAc	204.087		
		2HexNAc	407.168		
		HexNAcHex	366.140		
		HexNAc2Hex	528.193		
		HexNAc3Hex	690.249		

		2HexNAcHex	569.220		
		2HexNAc2Hex	731.273		

T236

Sample = 2nd virus chymotrypsin N+O   using 6 most O search

Sequence = EPLVDLPIGINITRF;

Mods = NGlycan/1054.3700; OGlycan/947.3230/HexNAc(2)Hex(4),HexNAc(1)Hex(1)NeuAc(2)

z=2

Error (ppm) = 9.95

Retention Time (min) = 142.08

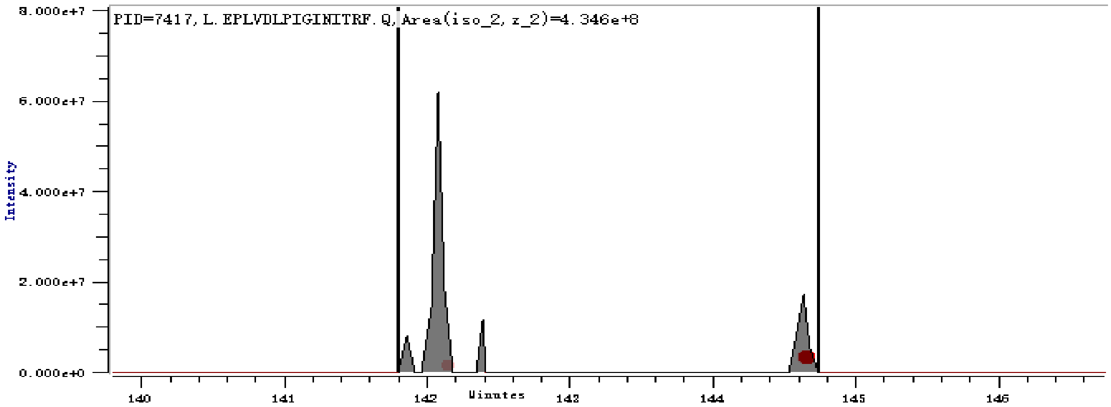

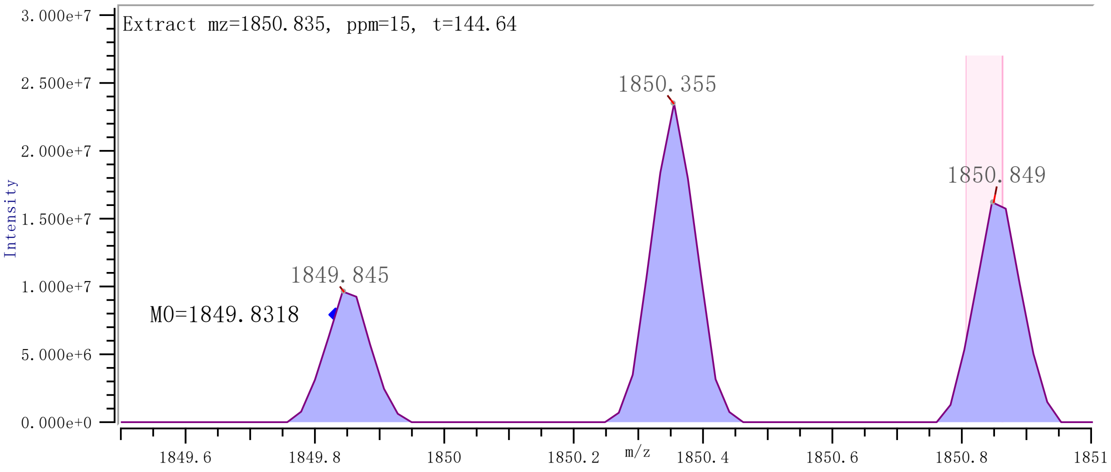

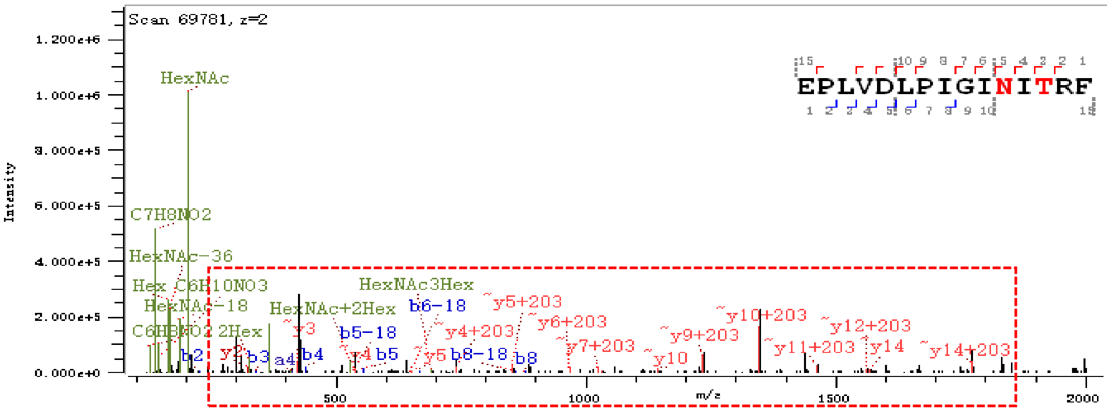

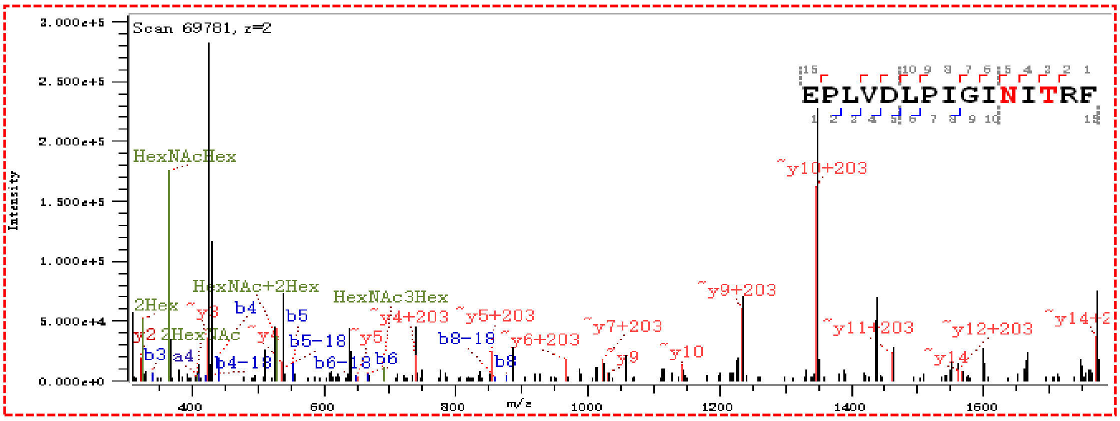

Diagnostic ions	observed m/z	B ions	observed m/z	Y ions	observed m/z
~ y4+203	739.406	C6H8NO2	126.055		
		C7H8NO2	138.055		
		C6H10NO3	144.065		
		Hex-36	127.039		
		Hex-18	145.049		
		Hex	163.060		
		2Hex	325.113		
		HexNAc-18	186.076		

		HexNAc-36	168.066		
		HexNAc	204.087		
		2HexNAc	407.166		
		HexNAcHex	366.140		
		HexNAc2Hex	528.193		
		HexNAc3Hex	690.246		

S305 & T307

Sample = SARS-COV-2 Virus S _deglycan_ O18_ Lytic_semi_78O 2021-4-11

Sequence = KCTLKSFT;

Mods = OGlycan/1238.4184; OGlycan/365.1322/HexNAc(1)Hex(1),HexNAc(1)Hex(1)NeuAc(3)

z=3

Error (ppm) = 1.15

Retention Time (min) = 20.43

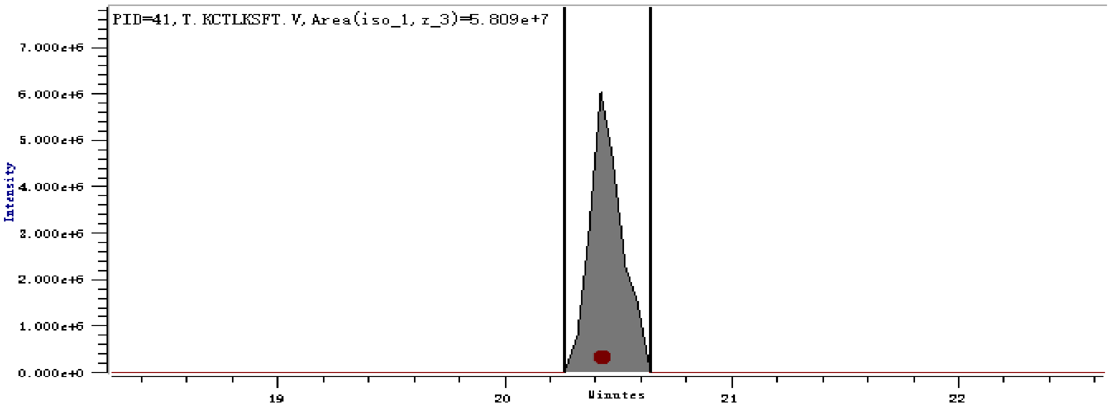

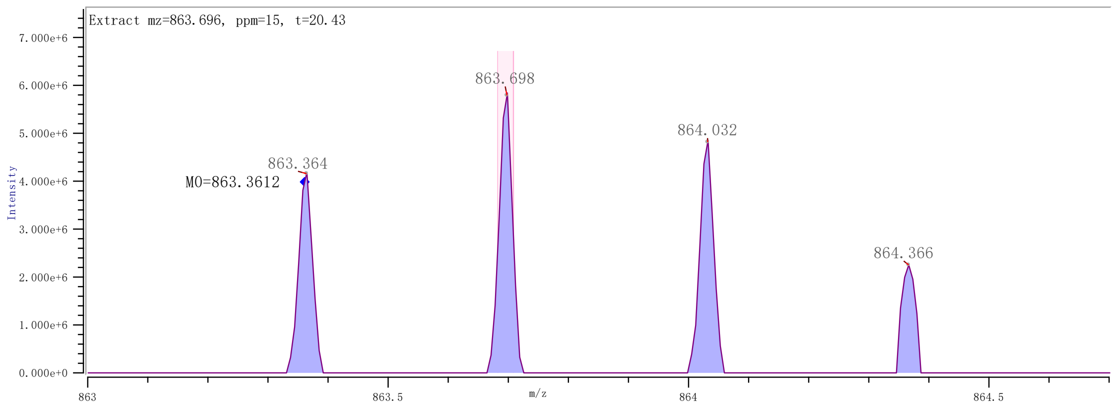

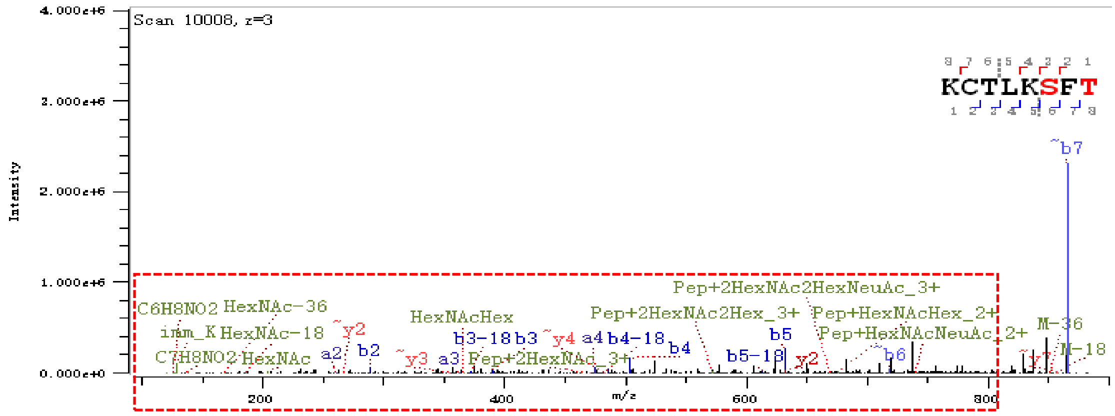

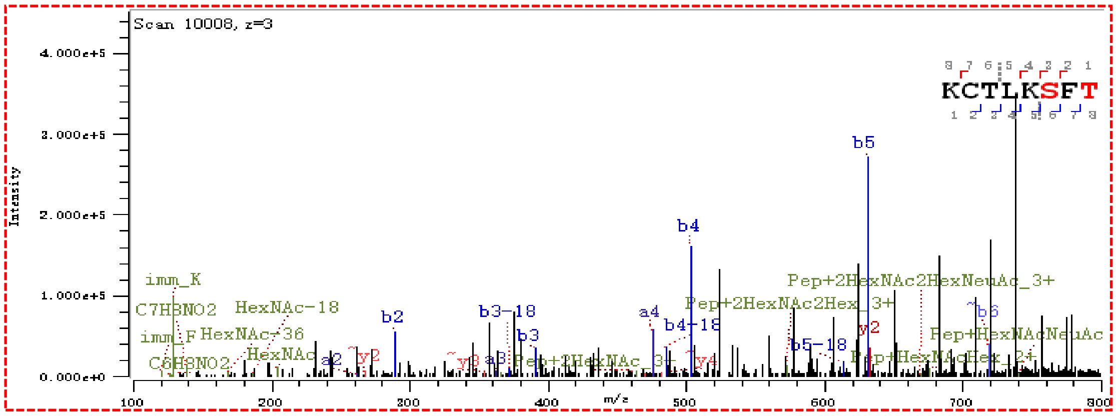

Diagnostic<br><br>ions	observed<br><br>m/z	B ions	observed<br><br>m/z	Y ions	observed<br><br>m/z
~y2+203	470.187	C6H8NO2	126.055	M-36	851.403
		C7H8NO2	138.055	M-18	857.380

		HexNAc-36	168.066	Pep+2HexNAc_3+	464.251
		HexNAc-18	186.076	Pep+HexNAcHex_2+	675.328
		HexNAc	204.087	Pep+2HexNAc2Hex_3+	572.287
		HexNAcHex	366.141	Pep+HexNAcNeuAc_2+	739.923
				Pep+2HexNAc2HexNeuAc_3+	669.340

T323

Sample = 4th virus trypsin HCD20 N+O using 6 most O 2021-6-29

Sequence = VQPTESIVR; Mods = OGlycan/203.0794/HexNAc(1)

z=2

Error (ppm) = 2.70

Retention Time (min) = 12.50

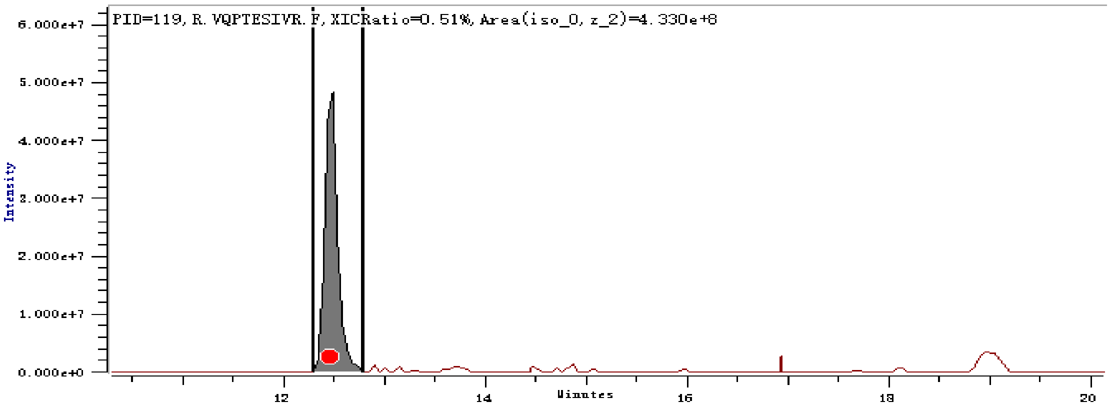

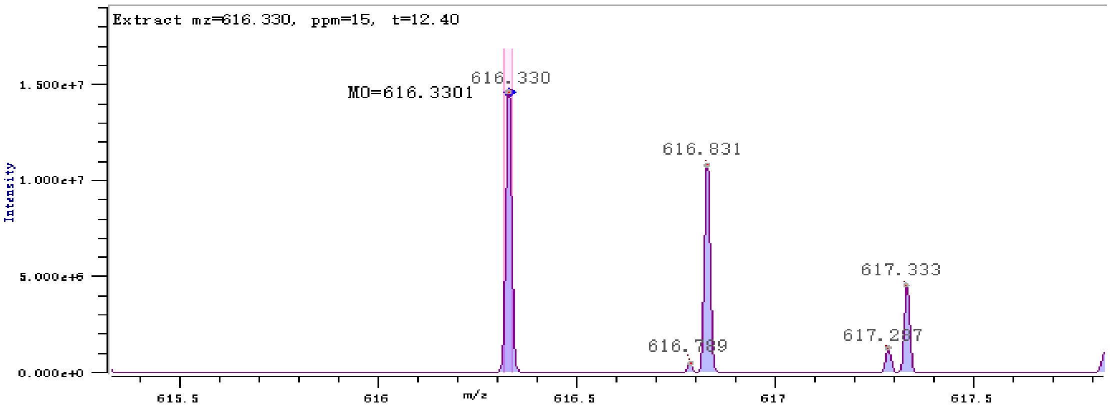

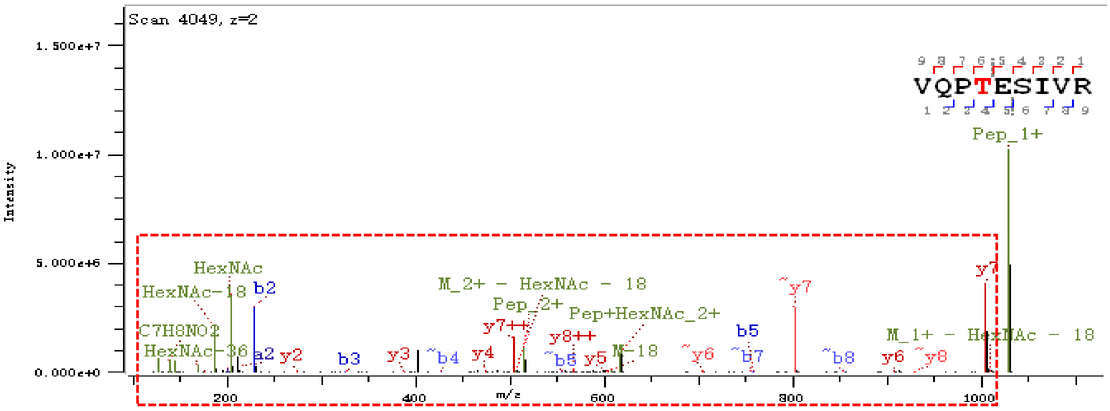

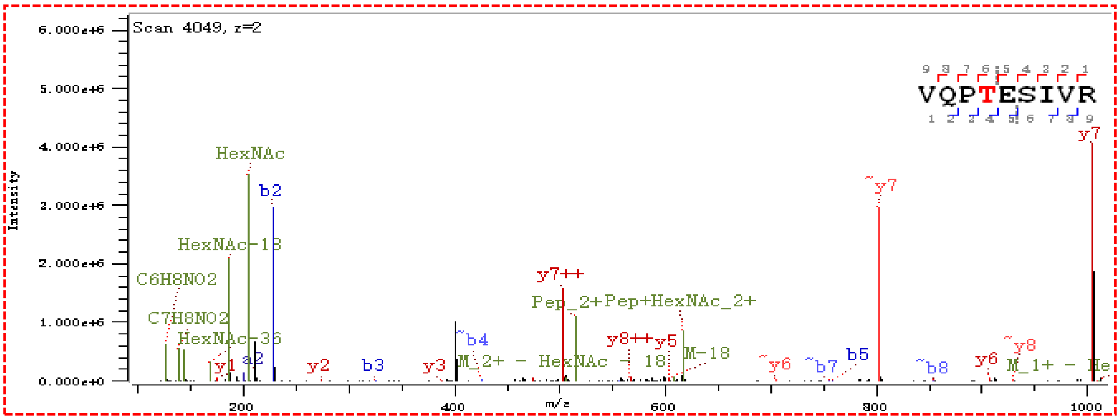

Diagnostic ions	observed m/z	B ions	observed m/z	Y ions	observed m/z
b3	325.188	C6H8NO2	126.055	M-18	607.331
b5	758.354	C7H8NO2	138.055	M_1+ - HexNAc-18	505.788
y5	603.349	C6H10NO3	144.065	M_2+ - HexNAc-18	1010.564
y7	1004.530	HexNAc-36	168.066	Pep _1+	1028.578
y7++	502.769	HexNAc-18	186.076	Pep _2+	514.793
		HexNAc	204.087	Pep+HexNAc_2+	616.332

T604 / S605

Sample = 4th virus chymotrypsin HCD15 N+O using 6 most O 2021-6-29

Sequence = GGVSVITPGTNTSNQVAVLY;

Mods = NGlycan/1378.4757; OGlycan/365.1322/HexNAc(2)Hex(6),HexNAc(1)Hex(1)

z=3

Error (ppm) = 6.99

Retention Time (min) = 100.76

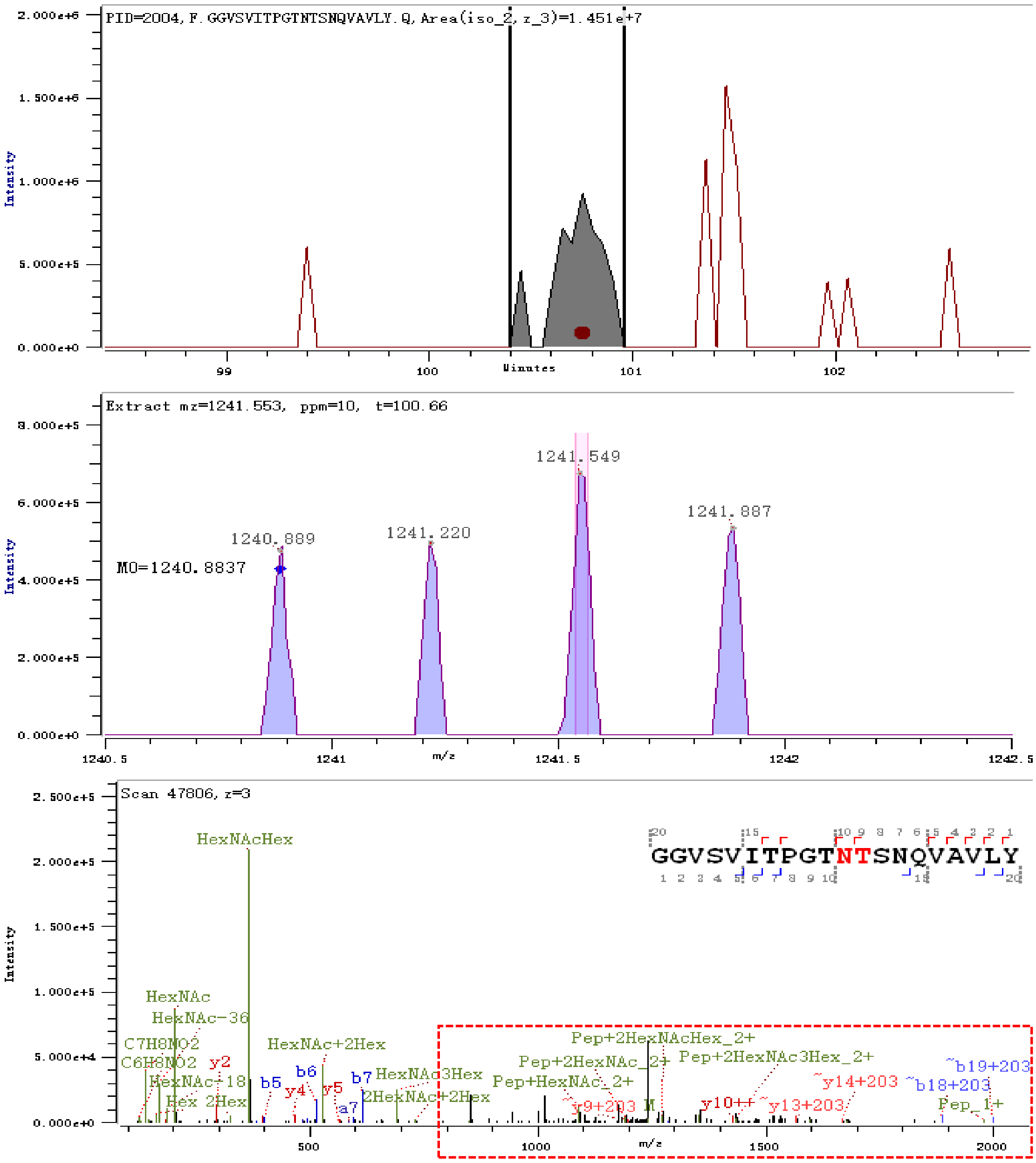

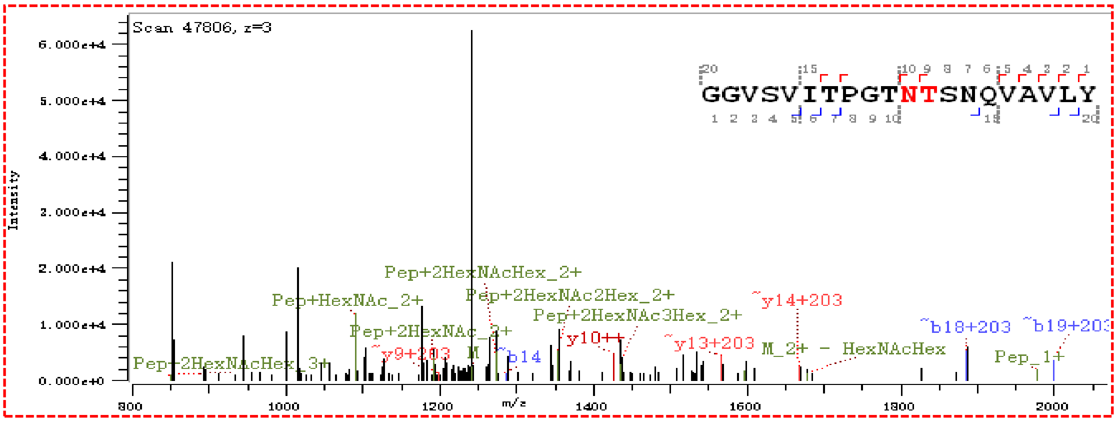

Diagnostic ions	observed m/z	B ions	observed m/z	Y ions	observed m/z
~y9+203	1197.545	C6H8NO2	126.055	M	1240.946
y10++	1426.664	C7H8NO2	138.055	M_2+ - HexNAcHex	1678.252
		C6H10NO3	144.066	Pep_1+	1977.023
		HexNAc-18	186.077	Pep+HexNAc_2+	1090.557
		Hex-36	127.038	Pep+2HexNAcHex_2+	1273.122
		Hex-18	145.050	Pep+2HexNAcHex_3+	849.089
		Hex	163.061	Pep+2HexNAc2Hex_2+	1354.148
		2Hex	325.114	Pep+2HexNAc3Hex_2+	1435.177
		HexNAc-36	168.066	Pep+2HexNAc5Hex_2+	1597.214
		HexNAc	204.087		
		HexNAcHex	366.140		
		HexNAc2Hex	528.194		
		HexNAc3Hex	690.247		
		2HexNAc2Hex	731.273		

T618

Sample = SARS-COV-2 Virus S _deglycan_ O18_ Lytic_semi_78O 2021-4-11

Sequence = LYQDVNCTEVPV;

Mods = Deamidated/2.9883; OGlycan/203.0794/HexNAc(1)

z=2

Error (ppm) = 0.47

Retention Time (min) = 101.36

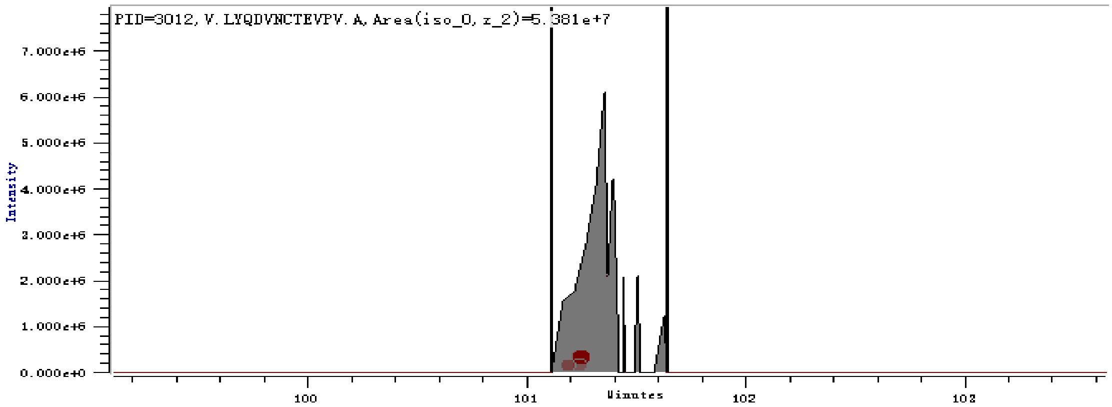

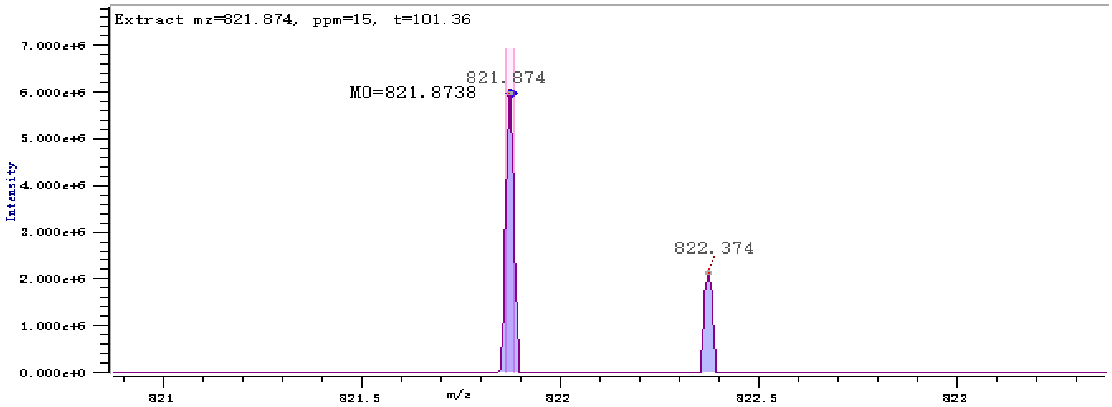

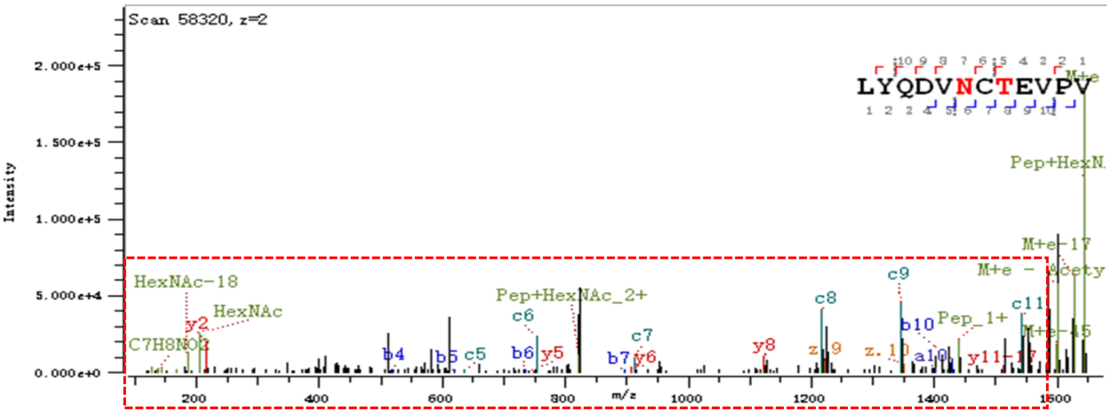

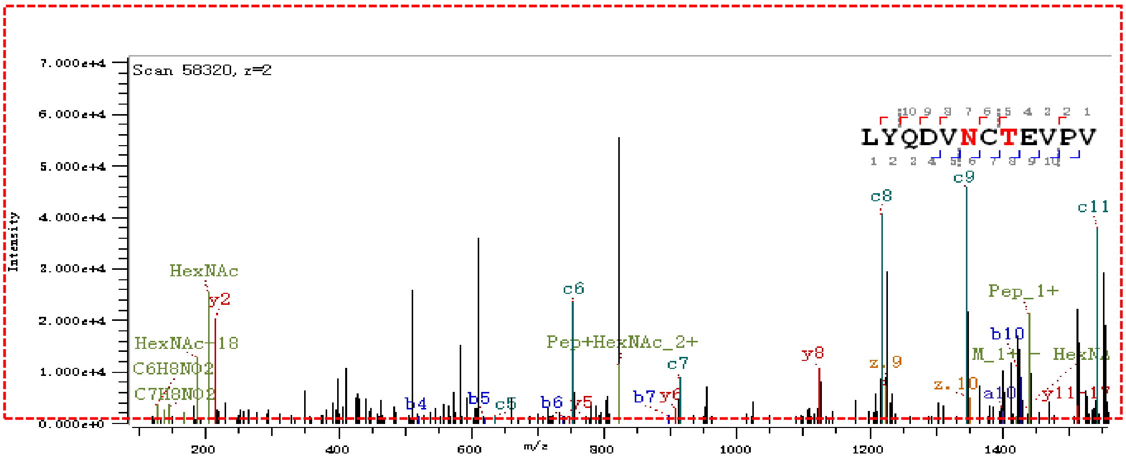

Diagnostic ions	observed m/z	B ions	observed m/z	Y ions	observed m/z
b6	736.343	C6H8NO2	126.055	M_1+ - HexNAc-18	1421.651
b7	896.369	C7H8NO2	138.055	Pep_1+	1439.661
y5	747.381	C6H10NO3	144.066	Pep+HexNAc_1+	1642.751
y6	907.403	HexNAc-36	168.065		
c5	636.332	HexNAc-18	186.075		
c6	753.366	HexNAc	204.086		
c7	913.396				
c8	1217.522				

S659

Sample = SARS-COV-2 Virus S _deglycan_ O18_ trypsin_ semi_78O 2021-4-11

Sequence = AGCLIGAEHVNNSYECDIPIGAGICASYQTQTNSPR;

Mods = Deamidated/2.9883; OGlycan/656.2276/HexNAc(1)Hex(1)NeuAc(1)

z=4

Error (ppm) = -0.81

Retention Time (min) = 130.90

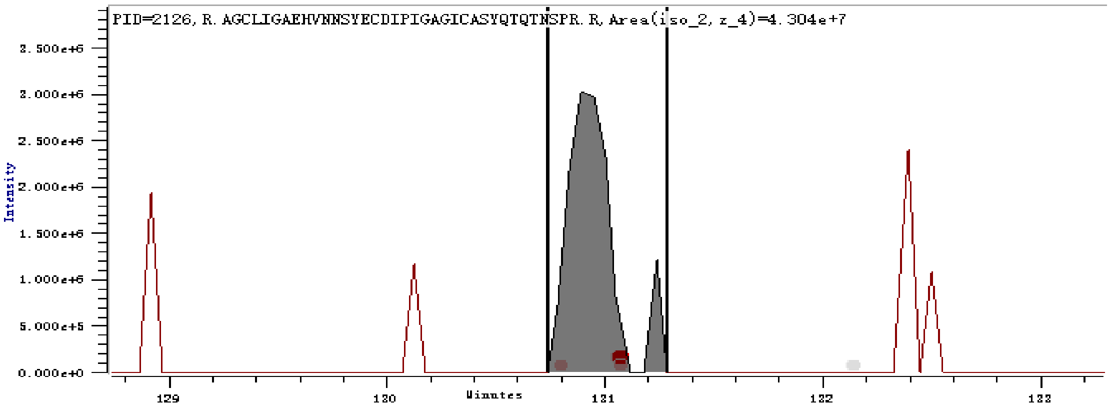

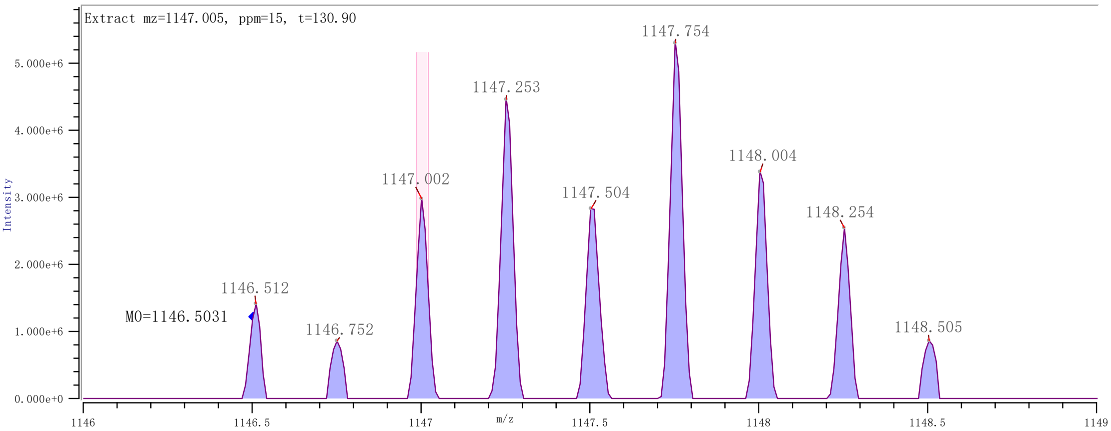

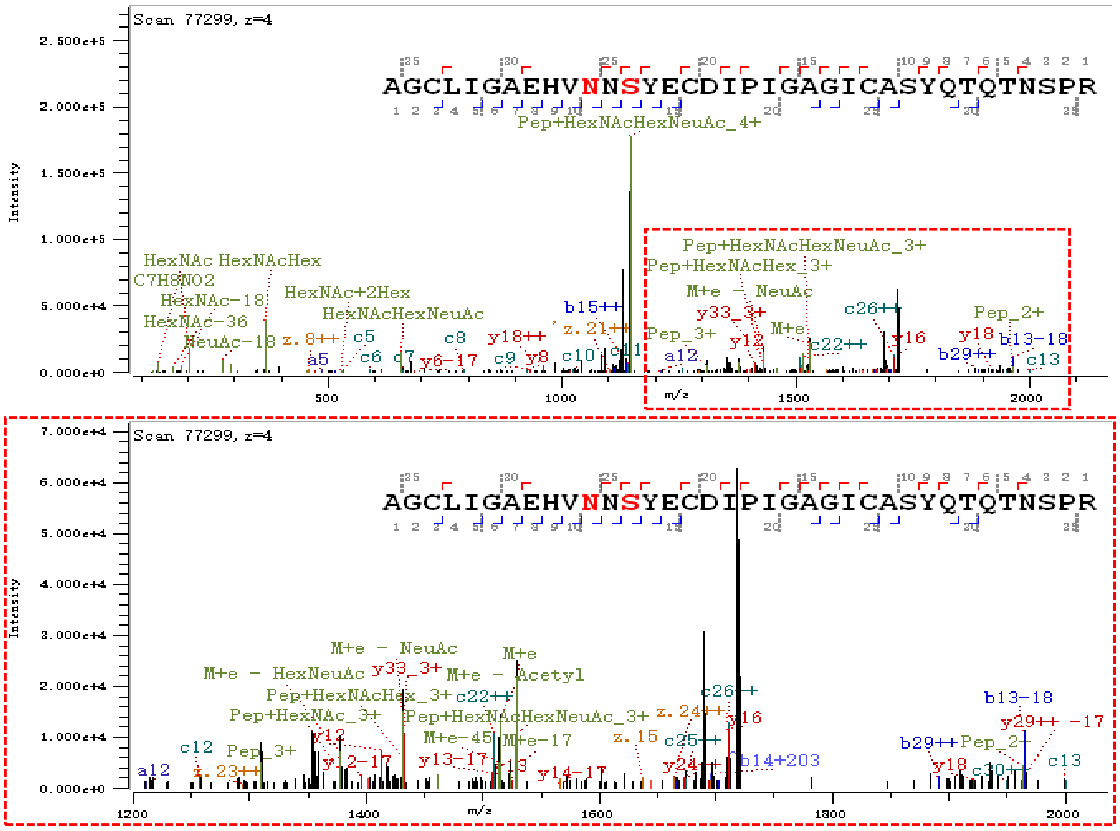

Diagnostic ions	observed m/z	B ions	observed<br><br>m/z	Y ions	observed<br><br>m/z
c10	1025.518	C6H8NO2	126.055	Pep_2+	1963.879
c11	1142.545	C7H8NO2	138.055	Pep_3+	1309.595
c12	1256.602	HexNAc-36	168.065	Pep+HexNAc_3+	1377.291
c13	1999.874	HexNAc-18	186.075	Pep+HexNAcHex_3+	1431.305
y24++	1672.756	HexNAc	204.086	Pep+ HexNAcHexNeuAc<br><br>_3+	1528.355
z23++	1293.069	HexNAcHex	366.139	Pep+ HexNAcHexNeuAc<br><br>_4+	1146.531
z24++	1664.761	HexNAcHexNeu	657.236		

		Ac			
		HexNeuAc	454.157		
		NeuAc-18	274.092		
		NeuAc	292.103		

T696

Sample = SARS-COV-2 Virus S _deglycan_ O18_ chymotrypsin_ semi_78O 2021-4-11

Sequence = TMSLGAENSVAY; Mods = OGlycan/203.0794/HexNAc(1)

z=2

Error (ppm) = 0.73

Retention Time (min) = 82.16

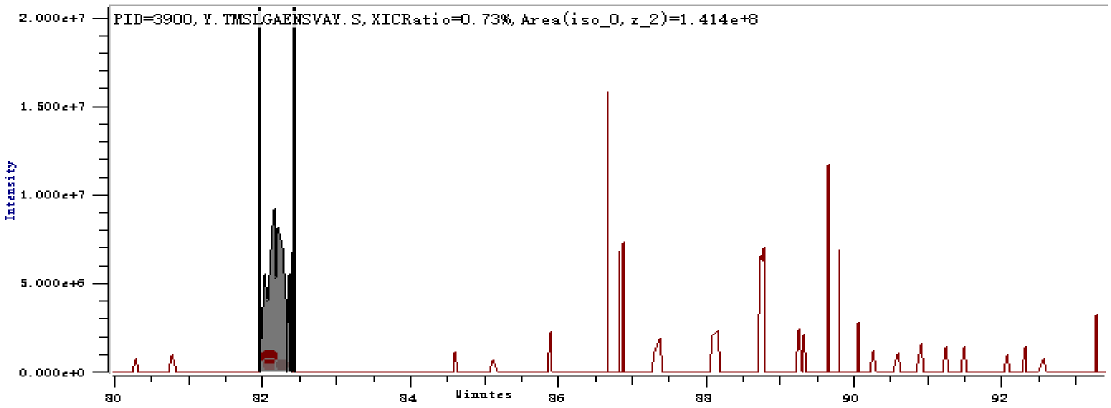

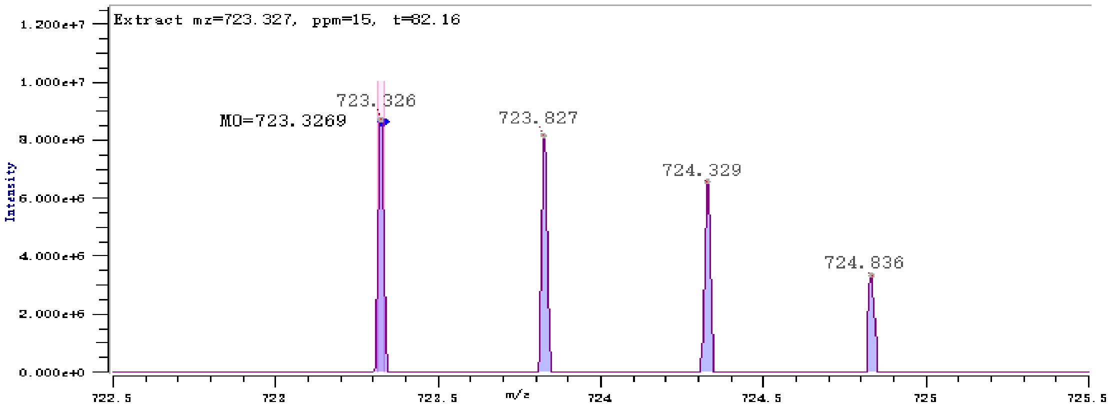

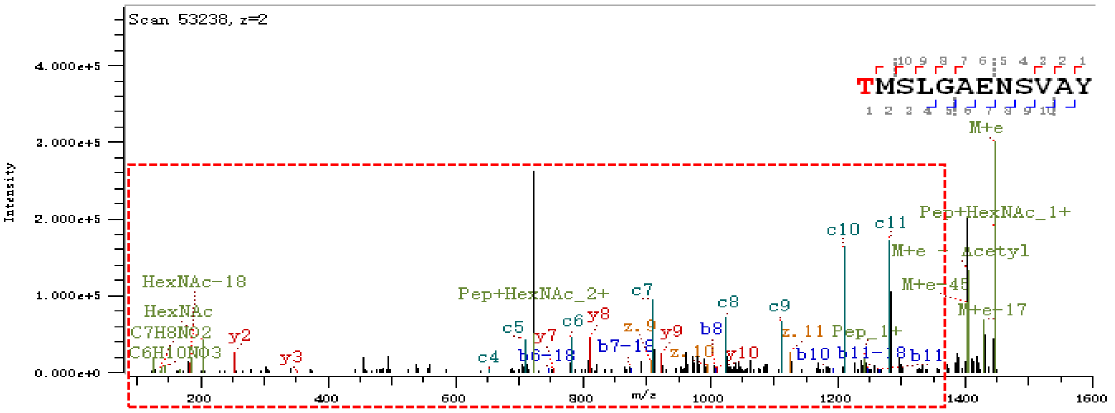

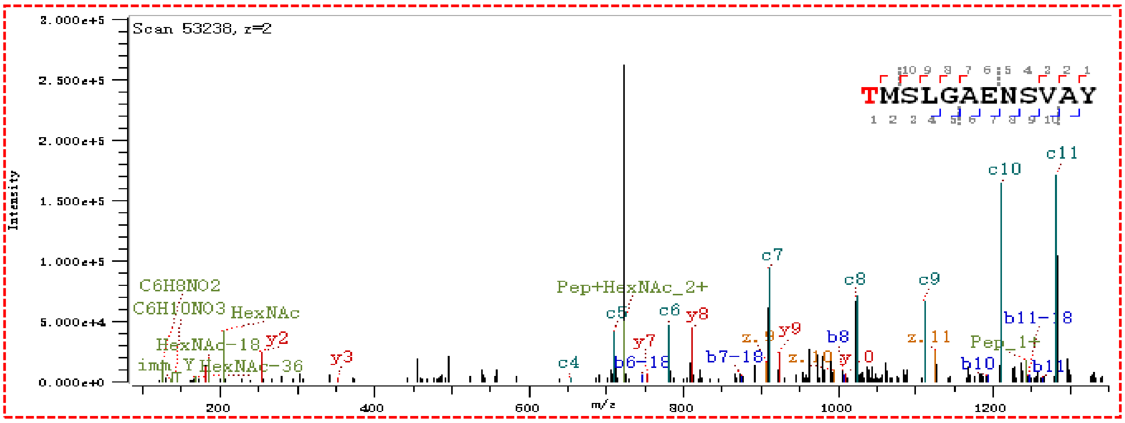

Diagnostic ions	observed m/z	B ions	observed m/z	Y ions	observed m/z
y10	1010.473	C6H8NO2	126.055	Pep_1+	1242.564
z10	994.459	C7H8NO2	138.055	Pep+HexNAc_1+	1445.666
z11	1125.501	C6H10NO3	144.065	Pep+HexNAc_2+	723.388
		HexNAc-36	168.065		
		HexNAc-18	186.075		
		HexNAc	204.086		

T724

Sample = 4th virus Lytic HCD20   N+O   using 6 most O 2021-7-3

Sequence = TEILPV; Mods = OGlycan/203.0794/HexNAc(1)

z=1

Error (ppm) = 2.11

Retention Time (min) = 62.68

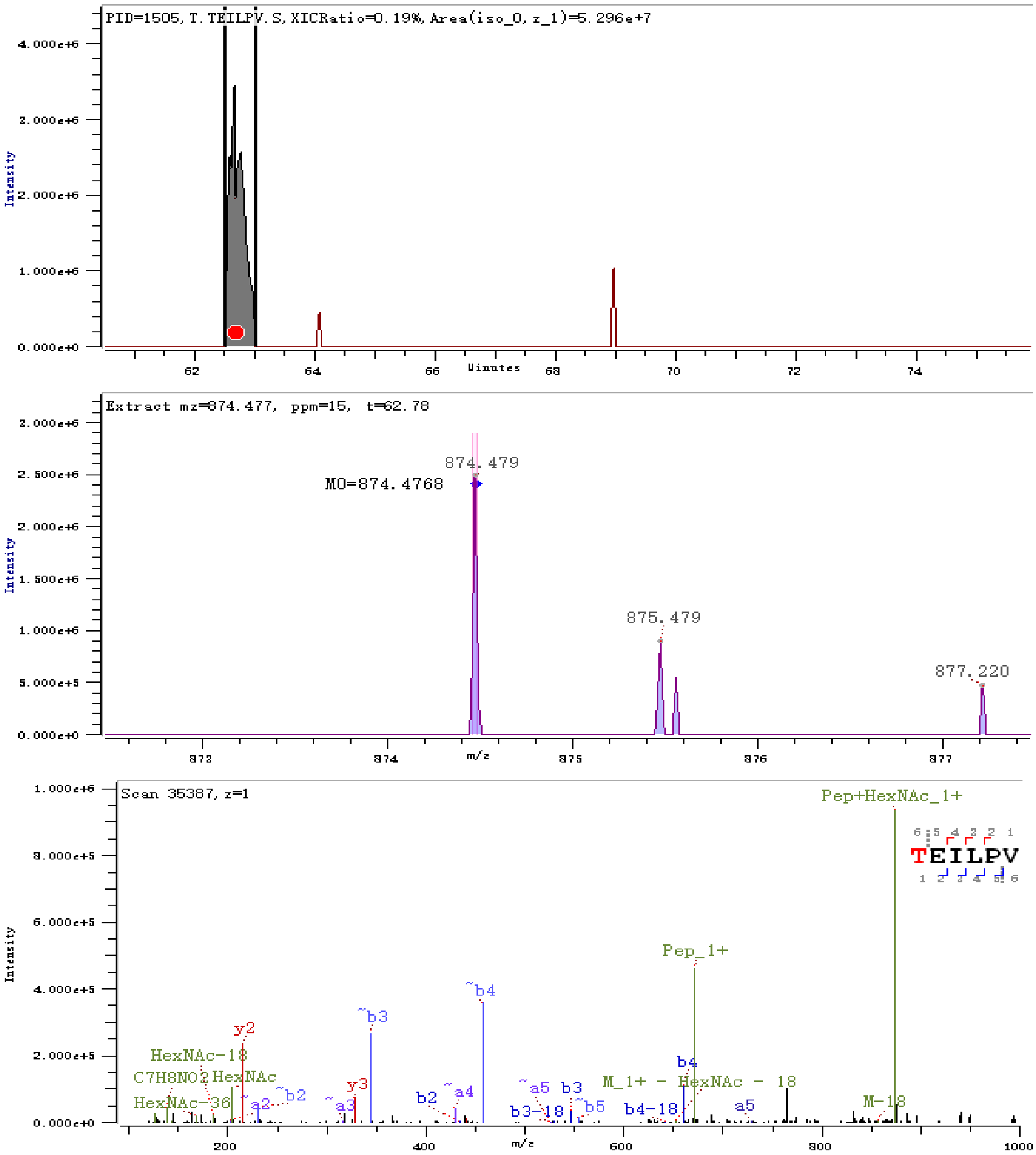

Diagnostic	observed	B ions	observed m/z	Y ions	observed m/z

ions	m/z				
~b2	231.098	C6H8NO2	126.055	M_1+ - HexNAc-18	653.380
~a2	203.103	C7H8NO2	138.055	Pep+HexNAc_1+	874.480
a2	406.171	C6H10NO3	144.065	Pep_1+	671.397
		HexNAc-36	168.065		
		HexNAc-18	186.075		
		HexNAc	204.086		

T1076

Sample = 4th virus trypsin stepHCD20 N+O using 6 most O 2021-6-29

Sequence = NFTTAPAICHDGK;

Mods=NGlycan/1200.4279; OGlycan/656.2276/HexNAc(2)Hex(4)Fuc(1),HexNAc(1)Hex(1)NeuAc(1)

z=3

Error (ppm) = 2.58

Retention Time (min) = 20.21

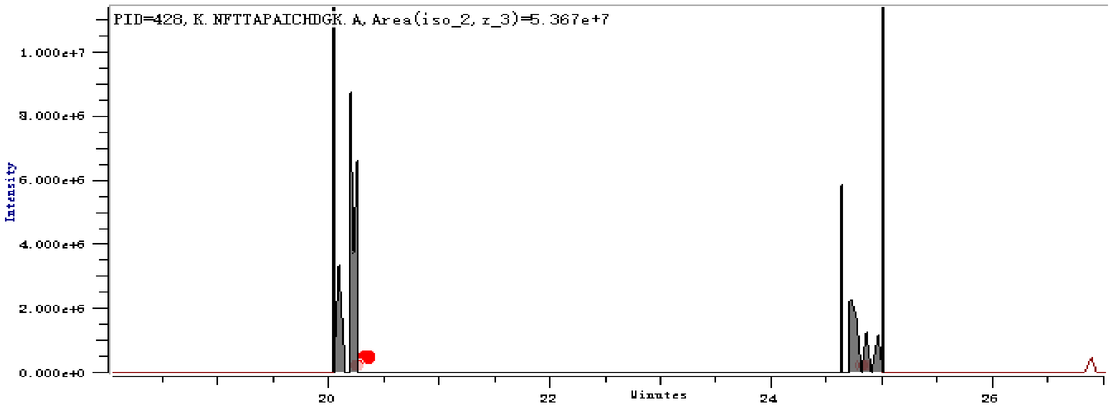

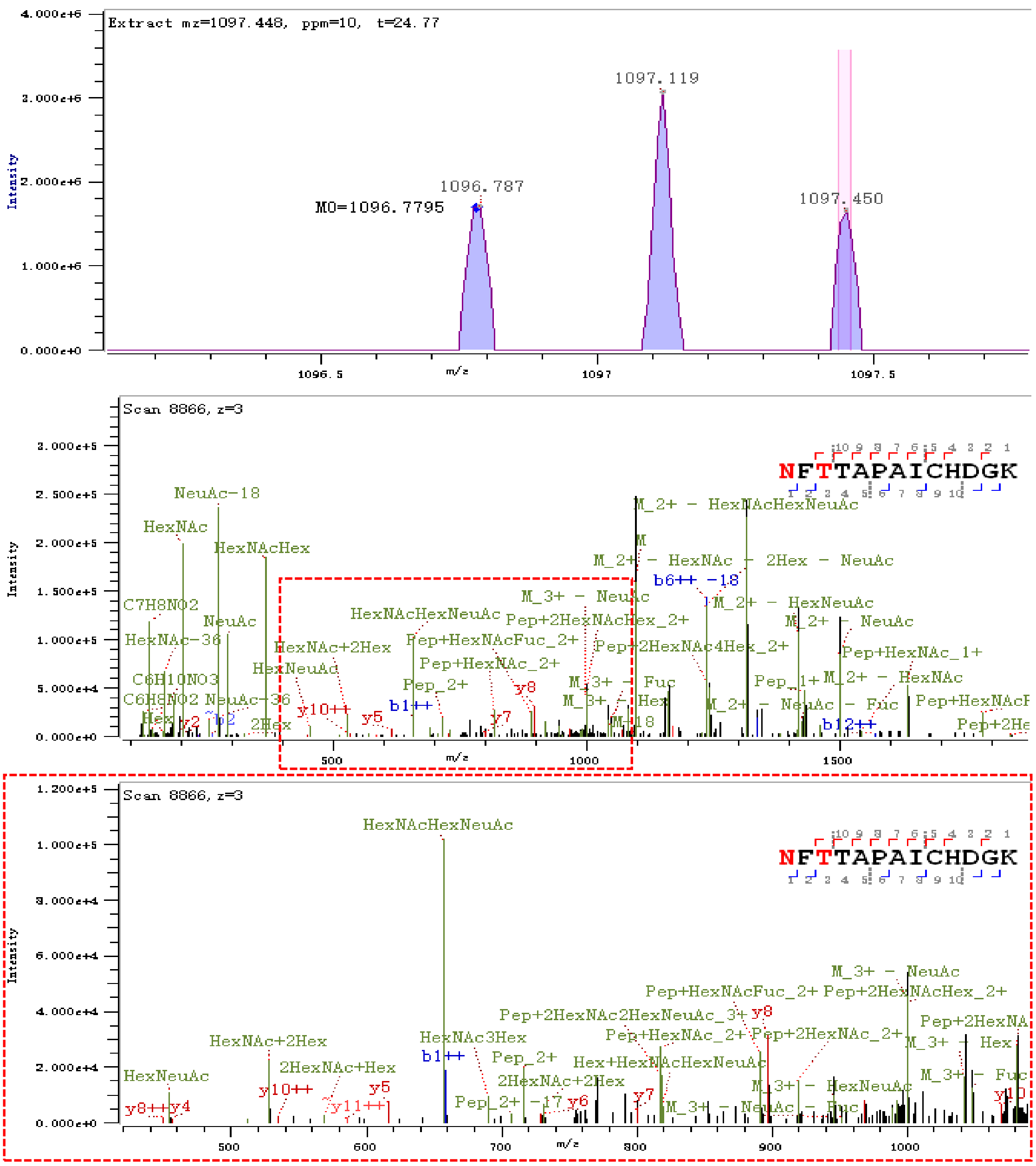

Diagnostic ions	observed m/z	B ions	observed m/z	Y ions	observed m/z
b1++	658.241	C6H8NO2	126.055	M - 18	1090.767
b2	1462.618	C7H8NO2	138.055	M	1096.783
y10++	535.256	C6H10NO3	144.065	M_2+ - HexNAcHex	1462.105

y10	1069.511	Hex-36	127.038	M_2+ - HexNAcHexNeuAc	1316.555
		Hex-18	145.050	M_2+ - HexNAc2Hex NeuAc	1235.529
		Hex	163.061	M_2+ - HexNeuAc	1418.096
		2Hex	325.114	M_3+ - HexNeuAc	945.733
		HexNAc-36	168.065	M_2+ - NeuAcFuc	1426.093
		HexNAc-18	186.075	M_3+ - NeuAcFuc	951.064
		HexNAc	204.086	M_2+ - NeuAc	1499.123
		HexNAcHex	366.140	M_3+ - NeuAc	999.751
		HexNAc2Hex	528.194	M_3+ - 2Hex	988.745
		HexNAc3Hex	690.248	M_3+ - Hex	1042.765
		2HexNAcHex	569.222	M_3+ - Fuc	1048.096
		2HexNAc2Hex	731.275	Pep_2+ - 17	707.831
		HexNeuAc	454.158	Pep_2+	716.340
		HexNAcHexNeuAc	657.237	Pep_1+ - 17	1414.648
		HexHexNAcFuc	512.199	Pep_1+	1431.671
		HexHexNAcHexNeuAc	819.290	Pep+HexNAc_1+	1634.752
		NeuAc-36	256.082	Pep+HexNAc_2+	817.880
		NeuAc-18	274.093	Pep+2HexNAc_1+	1837.821
		NeuAc	292.104	Pep+2HexNAc_2+	919.419
				Pep+2HexNAcHex_2+	1000.424
				Pep+2HexNAc2Hex_2+	1081.472
				Pep+2HexNAc3Hex_2+	1162.499

				Pep+2HexNAc4Hex_2+	1243.527
				Pep+HexNAcFuc_2+	890.909
				Pep+HexNAcFuc_1+	1780.812
				Pep+2HexNAcFuc_2+	992.446
				Pep+2HexNAc2HexNeuAc_2+	1227.025
				Pep+2HexNAc2HexNeuAc_3+	818.381

T1077

Sample = 2nd virus trypsin N+O search

Sequence = NFTTAPAICHDGK;

Mods=NGlycan/1200.4279; OGlycan/656.2276/HexNAc(2)Hex(4)Fuc(1),HexNAc(1)Hex(1)NeuAc(1)

z=3

Error (ppm) = 0.02

Retention Time (min) = 41.31

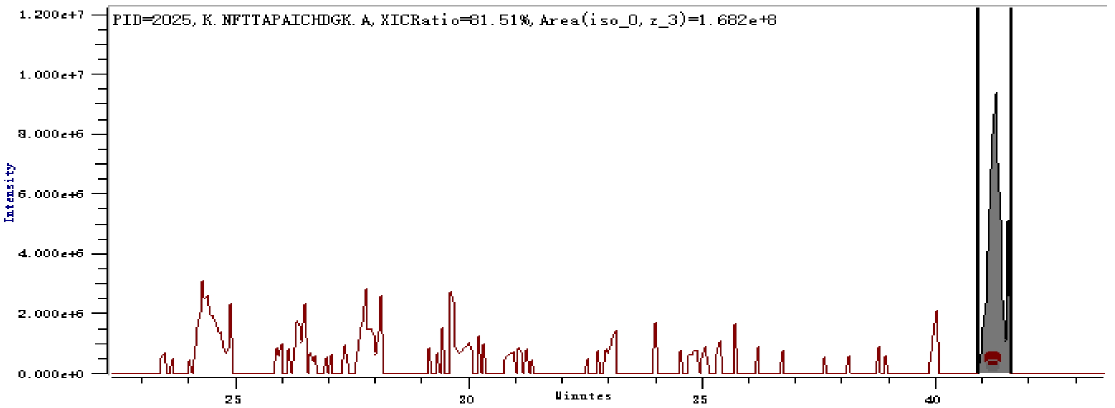

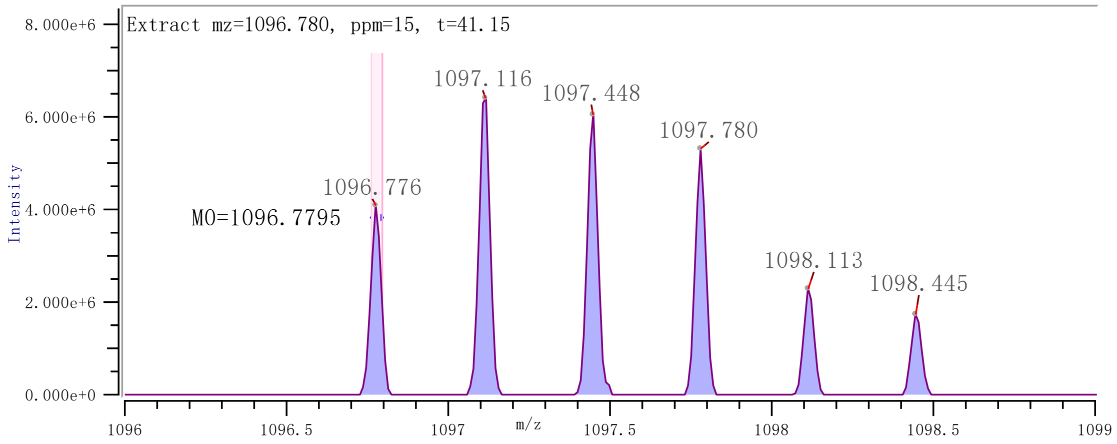

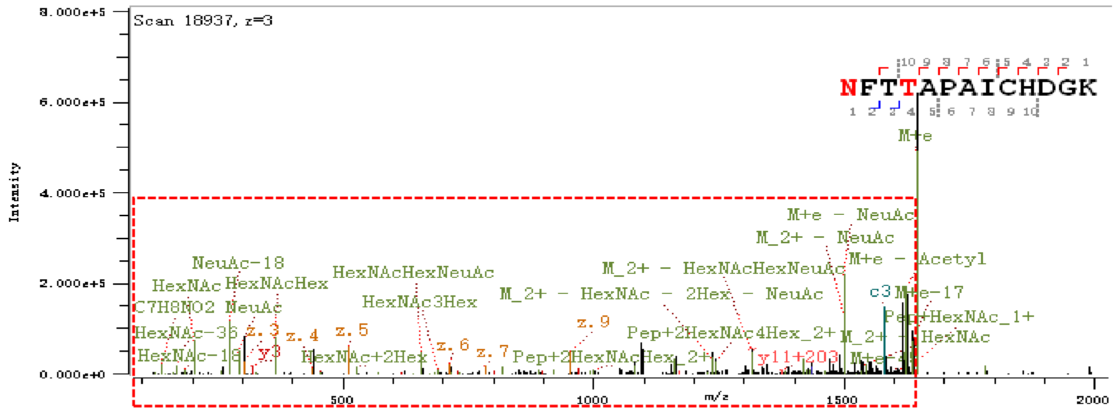

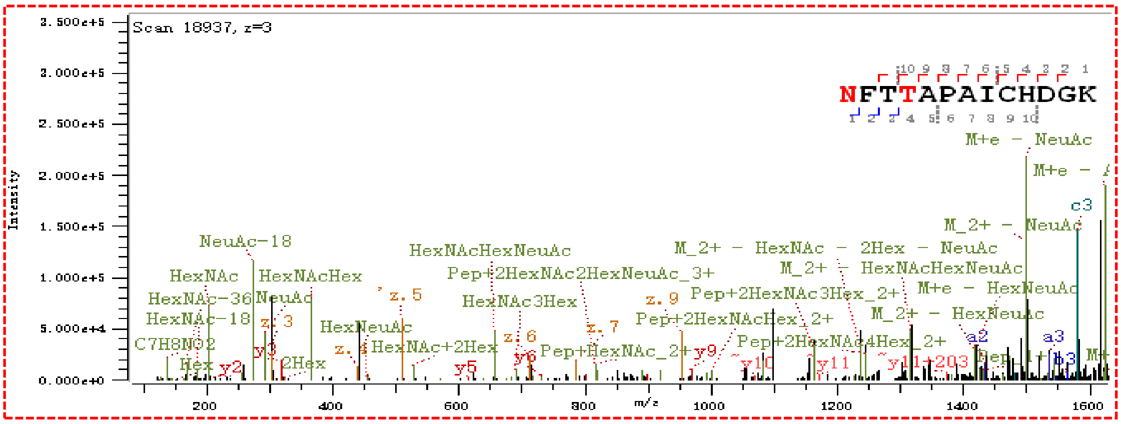

Diagnostic ions	observed<br><br>m/z	B ions	observed<br><br>m/z	Y ions	observed<br><br>m/z
c3	1580.626	C6H8NO2	126.055	M_2+ - HexNAc2HexNeuAc	1235.524

a3	1535.616	C7H8NO2	138.055	M_2+ - HexNAcHexNeuAc	1316.549
~y11+203	1373.632	C6H10NO3	144.065	M_2+ - HexNAc	1543.117
		Hex-18	145.049	M_2+ - HexNAc -18	1534.117
		Hex	163.061	M_2+ - HexNeuAc	1418.090
		2Hex	325.112	M_2+ - NeuAcFuc	1426.087
		HexNAc-36	168.065	M_2+ - NeuAc	1499.116
		HexNAc-18	186.076	Pep_1+	1431.663
		HexNAc	204.086	Pep_2+	716.336
		HexNAcHex	366.139	Pep+HexNAc_1+	1634.747
		HexNAc2Hex	528.192	Pep+HexNAc_2+	817.876
		HexNAc3Hex	690.244	Pep+2HexNAcHex_2+	1000.442
		2HexNAcHex	569.216	Pep+2HexNAc2Hex_2+	1081.468
		2HexNAc2Hex	731.270	Pep+2HexNAc3Hex_2+	1162.495
		HexNAcHexNeuAc-18	639.224	Pep+2HexNAc4Hex_2+	1243.522
		HexNAcHexNeuAc	657.234	Pep+HexNAcFuc_1+	1780.802
		HexHexNAcHex NeuAc	819.287	Pep+HexNAcFuc_2+	890.904
		HexNeuAc	454.155	Pep+2HexNAcFuc_2+	992.444
		HexNAc2HexFuc	674.246	Pep+2HexNAc2HexNeuAc_2+	1227.021
		NeuAc-36	256.081		
		NeuAc-18	274.092		

		NeuAc	292.102		

S1097

Sample = 4th virus chymotrypsin stepHCD25 N+O using 6 most O 2021-6-29

Sequence=VSNGTHWF;

Mods=OGlycan/365.1322;NGlycan/1378.4757/HexNAc(2)Hex(6),HexNAc(1)Hex(1)

z=2

Error (ppm) = 2.88

Retention Time (min) = 18.86

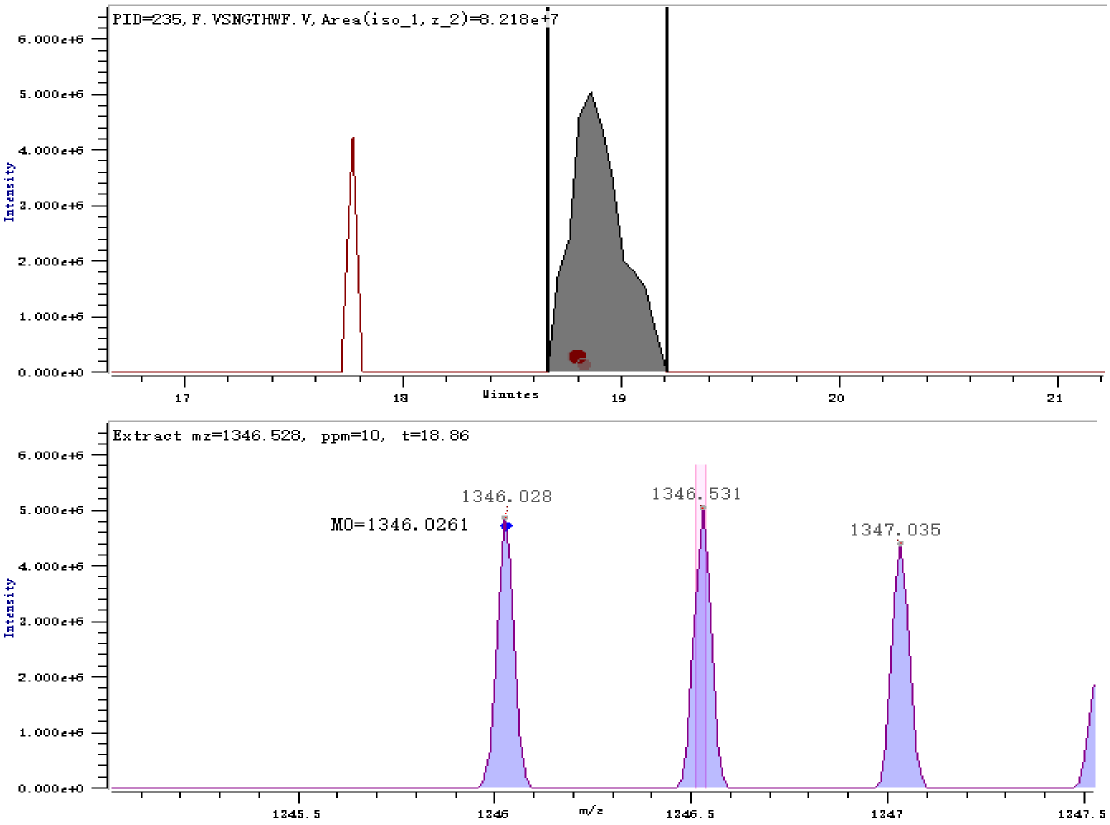

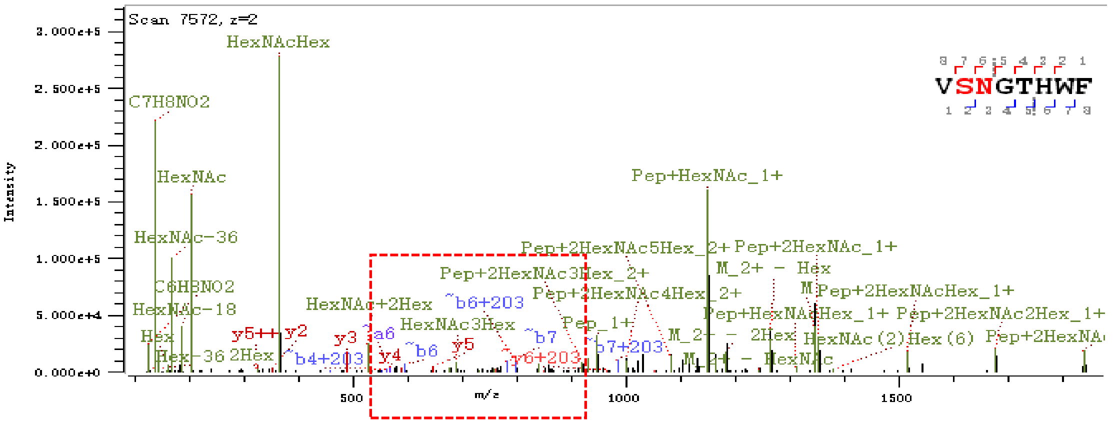

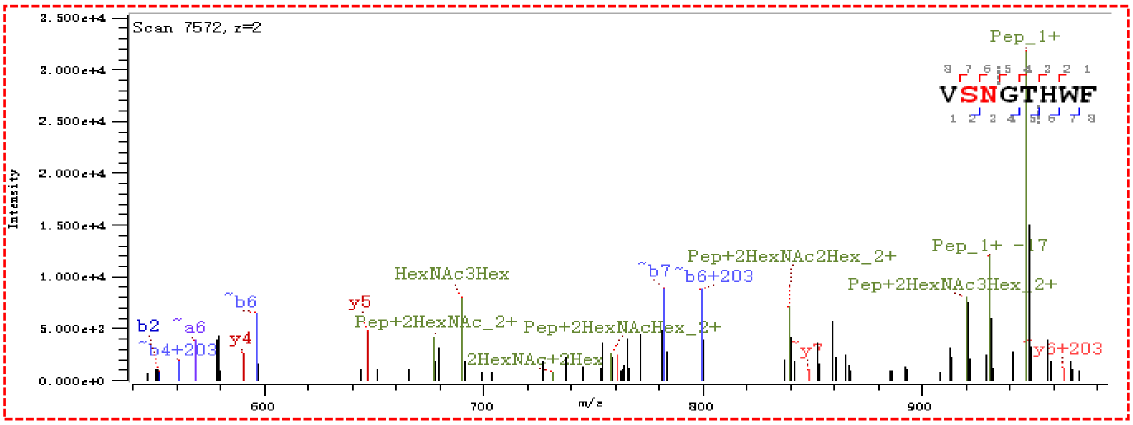

Diagnostic ions	observed m/z	B ions	observed m/z	Y ions	observed m/z
b2	552.254	C6H8NO2	126.055	M	1346.028
y4	590.274	C7H8NO2	138.055	M_2+ - HexNAcHex	1163.461
y5	647.295	C6H10NO3	144.066	M_2+ - HexNAc	1244.477
~y6+203	964.419	Hex-36	127.039	M_2+ - 2Hex	1183.974
		Hex-18	145.050	M_2+ - Hex	1265.010
		Hex	163.066	Pep_1+ - 17	930.413
		2Hex	325.114	Pep_1+	947.438
		HexNAc-36	168.061	Pep+HexNAc_1+	1150.518

		HexNAc-18	186.077	Pep+HexNAcHex_1+	1312.573
		HexNAc	204.087	Pep+2HexNAc_1+	
		HexNAcHex	366.141	Pep+2HexNAc_2+	
		HexNAc+2Hex	528.194	Pep+2HexNAcHex_1+	1515.649
		HexNAc3Hex	690.247	Pep+2HexNAcHex_2+	758.330
		2HexNAc+2Hex	731.276	Pep+2HexNAc2Hex_1+	1677.701
		2HexNAc6Hex	1379.485	Pep+2HexNAc2Hex_2+	839.356
			1353.597	Pep+2HexNAc3Hex_1+	1839.755
			677.303	Pep+2HexNAc3Hex_2+	920.382
				Pep+2HexNAc4Hex_2+	1001.409
				Pep+2HexNAc5Hex_2+	1082.435

T1100

Sample = 4th virus chymotrypsin HCDpdEThcD27 N+O using 6 most O 2021-6-29

Sequence = VSNGTHWF;

Mods = NGlycan/1378.4757; OGlycan/656.2276/HexNAc(2)Hex(6),HexNAc(1)Hex(1)NeuAc(1)

z=3

Error (ppm) = 2.77

Retention Time (min) = 36.85

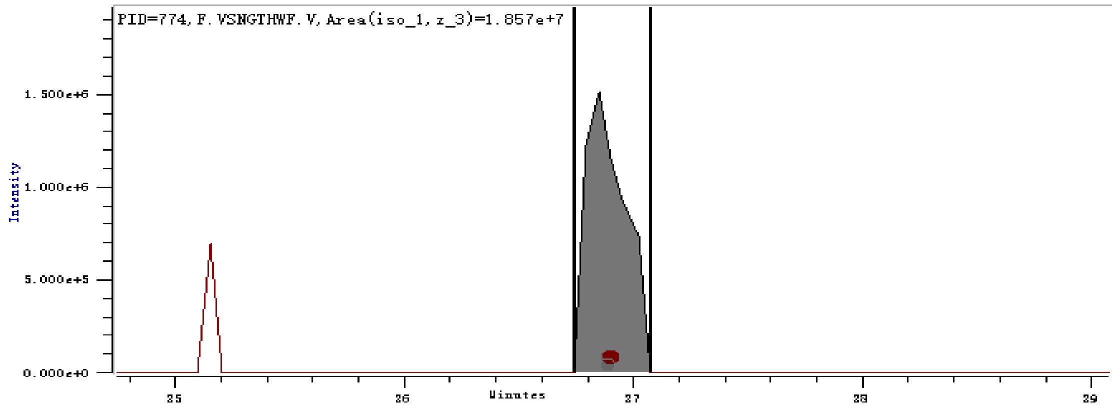
PID=774, F.VSNGTHWF.V, Area(iso_1, z_3)=1.857e+7
Intensity
Minutes

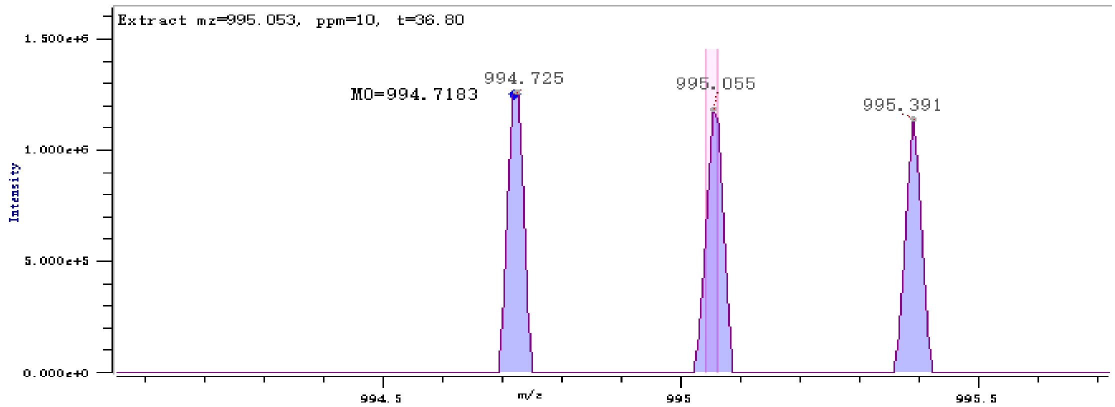
Extract mz=995.053, ppm=10, t=36.80
M0=994.7183
994.725
995.055
995.391
Intensity
m/z

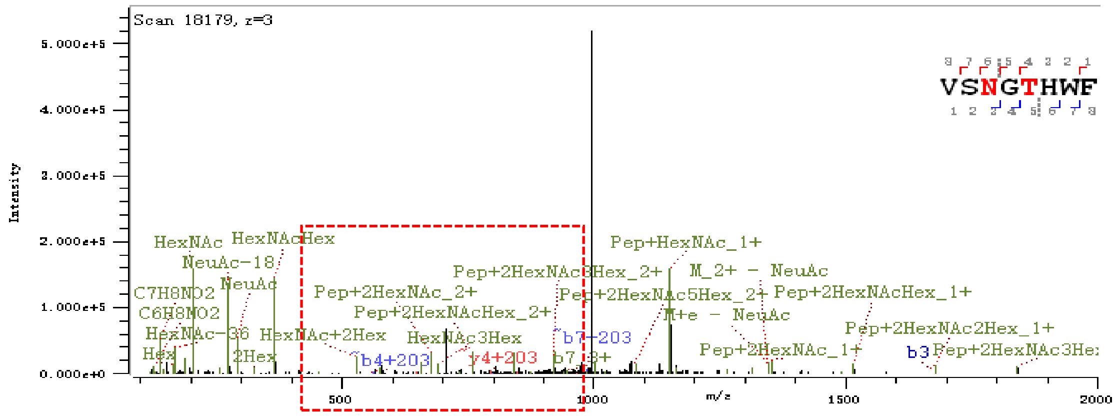
Scan 18179, z=3
VSNGTHWF
Intensity
m/z

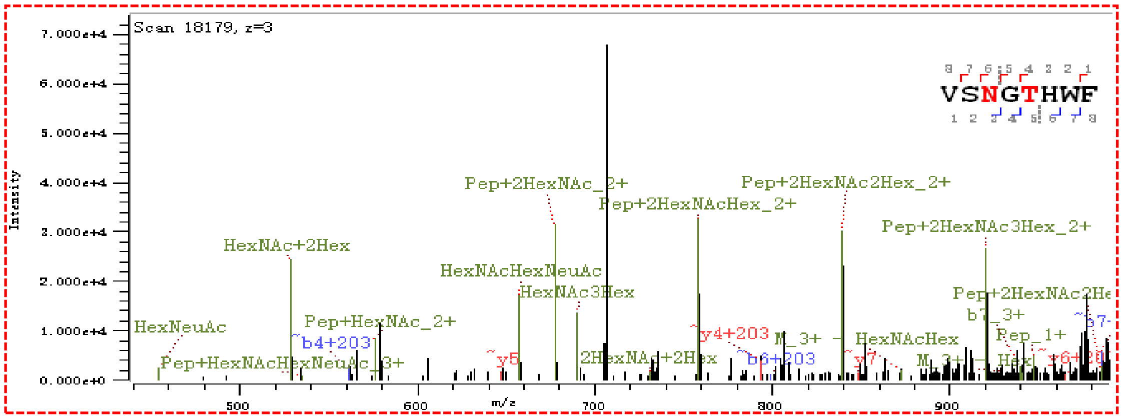
Scan 18179, z=3
VSNGTHWF
Intensity
m/z

Diagnostic ions	observed m/z	B ions	observed m/z	Y ions	observed m/z
b3	1679.706	C6H8NO2	126.055	M_3+ - Hex	940.789
~y4+203	793.388	C7H8NO2	138.055	M_3+ - HexNAcHex	872.930
		C6H10NO3	144.066	M_2+ - HexNAcHexNeuAc	1163.459
		Hex-36	127.038	M_2+ - HexNeuAc	1284.996
		Hex	163.061	M_2+ - NeuAc	1346.023
		2Hex	325.114	Pep_1+	947.437
		HexNAc-36	168.060	Pep+HexNAc_1+	1150.515
		HexNAc-18	186.076	Pep+HexNAc_2+	575.763
		HexNAc	204.087	Pep+2HexNAc_1+	1353.594
		HexNeuAc	454.157	Pep+2HexNAc_2+	677.362
		HexNAcHex	366.140	Pep+HexNAcHex_1+	1312.566
		HexNAc2Hex	528.193	Pep+HexNAcHexNeuAc_3+	535.225
		HexNAc3Hex	690.246	Pep+2HexNAcHex_1+	1515.645
		2HexNAc2Hex	731.271	Pep+2HexNAcHex_2+	758.328
		2HexNAc2HexNeuAc	1022.422	Pep+2HexNAc2Hex_1+	1677.698
		HexNAcHexNeuAc	657.236	Pep+2HexNAc2Hex_2+	839.354
		NeuAc-36	256.082	Pep+2HexNAc3Hex_1+	1839.074
		NeuAc-18	274.093	Pep+2HexNAc3Hex_2+	920.381

		NeuAc	292.103	Pep+2HexNAc4Hex_2+	1001.407
				Pep+2HexNAc5Hex_2+	1082.433
				Pep+2HexNAc2HexNeuAc_2+	984.956

Fig. S3. The identification of 18 *O-* glycosites of the S protein extracted from SARS-CoV-2 virions.

For each peptide/glycopeptide detected, the corresponding extracted ion chromatogram (XIC) (upper panel), precursor isotope distribution (middle panel), MS/MS spectrum with the enlarged view (lower panels) and Diagnostic ions, B, Y ions together with observed m/z (table) are shown. The monoisotopic peak is marked as "MO=" with a blue dot (middle panel). The fragment b- and y-ions are in blue and red color, respectively; the c- and z-ions are in cyan and orange color, respectively. The oxonium ions are in green color. The spectrum outlined by the red dotted line is the enlarged view of the highlighted region in the MS/MS spectrum above. The *O*-glycosite of T236 determined only by a series of B ions and one diagnostic peak. The *O*-glycosites of S305 and T307 lack of fragment ions of NeuAc. These are weak candidates of OFN.

T618

Sample = S-WT-TM-gluC-deglycan....byspec2_20200930_Byonic

Sequence = VNCTEVPVAIHAD; Mods = Deamidated/0.9840; OGlycan/609.2381/HexNAc(3)

z=2

Error (ppm) = -4.60

Retention Time (min) = 60.26

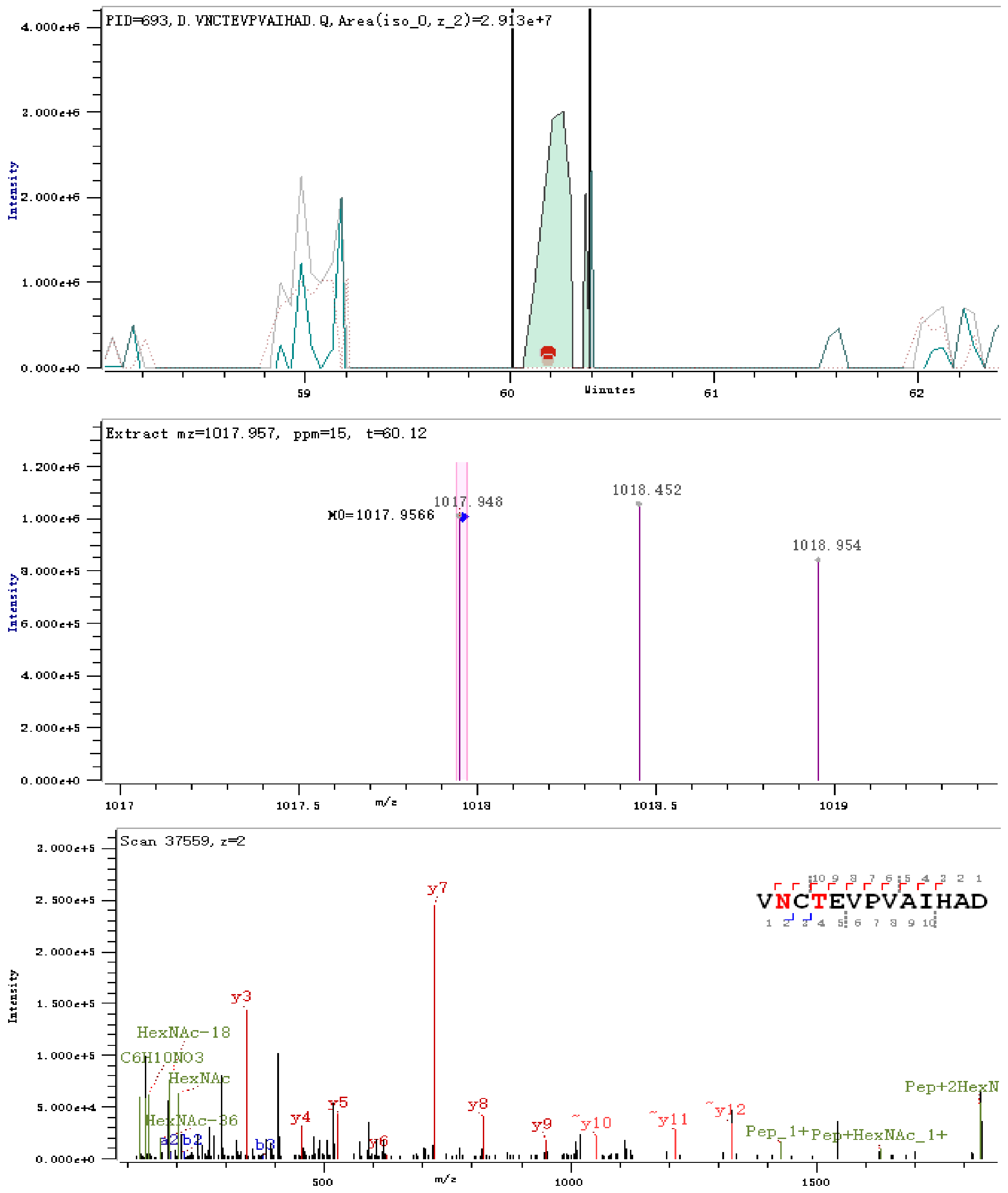

Fig. S4. The identification of *O*- glycosites at T618 of the WT full length S protein purified from 293T cell.

The corresponding extracted ion chromatogram (XIC) (upper panel), isotope distribution (middle panel), and MS/MS spectrum with the enlarged view (lower panels) are shown. The monoisotopic peak is marked as a blue dot (middle panel). The fragment b- and y-ions are in blue and red color, respectively; the c- and z-ions are in cyan and orange color, respectively. The oxonium ions are in green color.

Table S1. Overview of the identified *O*-glycosylation of the S protein extracted from SARS-CoV-2 virions.

Identified *O*-glycosylation	Near *N*-sequon position (OFN)	N ,O glycosylation search together[1]	Asn deamidation (+2.98Da)[1]	Glycan or oxonium ion[2]	Y ions[2]	Precursor isotopic pattern[2]	Correct retention time[2]	Diagnostic ions for glycosites determination[3]	Mutagenesis for OFN validation
T22		√		√	√	√	√	√	
S305		√		√	√	√	√		
T307		√		√	√	√	√		
T323		√		√	√	√	√	√	
T696		√		√	√	√	√	√	
T724		√		√	√	√	√	√	
S60	√	√		√	√	√	√	√	
T124	√	√		√	√	√	√	√	

**S151**	√	√		√	√	√	√	√	
**T236**	√	√		√		√	√	√	
**T604/ S605**	√	√		√	√	√	√		
**T618**	√		√	√	√	√	√	√	√
**S659**	√		√	√	√	√	√	√	
**T1076**	√	√		√	√	√	√	√	
**T1077**	√	√		√	√	√	√	√	
**S1097**	√	√		√	√	√	√	√	
**S1100**	√	√		√	√	√	√	√	

[1] Methods used for identification of *O*-glycosylation.

[2] Criteria used for characterization of *O*-glycopeptides.

[3] **T**he requisite criterion for determination of *O*-glycosites.

Methods

Expression constructs

The codon-optimized cDNA for the full-length wild type spike (S) protein (Uniprot: P0DTC2-1) was synthesized by the Genewiz Company (China, Beijing). S with a C-terminal Twin-Strep-Tag II (WSHPQFEKGGG SGGGSGGSAWSHPQFEK) was cloned into a mammalian cell expression vector, pCAGGS. All point mutations were introduced using mutagenic primers. The sequences of all the constructs were verified prior to cell transfection and protein purification.

Expression and purification of the recombinant proteins

The HEK 293T cells were cultured in Dulbecco's modified eagle medium (DMEM) with 10% fetal bovine serum (FBS) (Thermo Fisher Scientific) in 150 mm×25 mm dishes at 37°C with 5% CO_2. When the cells reached approximately 70% confluence, they were transfected with plasmids encoding the S protein. For one dish of HEK293T cells, 60 μg of plasmids were premixed with 60 μg of polyethylenimines (PEIs) in 2 ml of fresh medium for 15 to 30 min. The mixture was then added into the cell culture followed by 4~6 h of incubation. The cells were washed with PBS two times and cultured with fresh DMEM medium. For the purification of the full-length S protein, cells were harvested 4~5 days after transfection and resuspended in a lysis buffer containing 20 mM Tris, pH 8.0, 200 mM NaCl, 10% glycerol, and 1 mM phenylmethylsulfonyl fluoride (PMSF). The suspension was homogenized with an ATS homogenizer. The lysate was centrifuged at 200000 g for 80 min at 4°C. The membrane

fraction was resuspended in a buffer containing 20 mM Tris, pH 8.0, 200 mM NaCl, 10% glycerol, 0.2 mM PMSF, and 1% Triton X-100. After incubation at 4°C for 1 h, the suspension was clarified using an ultracentrifuge for 30 min. The supernatant was collected and loaded onto a Strep-Tactin column. After washing with a buffer containing 20 mM Tris, pH 8.0, 200 mM NaCl, 10% glycerol, and 0.05% Triton X-100, the proteins were eluted with a wash buffer plus 5 mM of D-Desthiobiotin. The eluent was concentrated using a 50-kDa cut-off Centricon and further purified using a Superose-6 Increase column (GE Healthcare) in a buffer containing 20 mM Tris, pH 8.0, 50 mM NaCl, and 0.01% Triton X-100. The fractions were analyzed by SDS-PAGE and verified using western blot. The peak fractions corresponding to the S protein were pooled and concentrated to approximately 0.5 mg/ml.

Recombinant purified S protein preparation for mass spectrometry analysis

50 μg of purified SARS-CoV-2 S protein was denatured for 1h in 50 mM Tris/HCl, pH 8.0 containing 6 M of urea and 5 mM dithiothreitol (DTT). Next, the S protein was reduced and alkylated by adding 20 mM iodoacetamide (IAA) and incubated for 1h in the dark, followed by a 1h incubation with 20 mM DTT to eliminate residual IAA. The alkylated S protein was buffer-exchanged into 50 mM Tris/HCl, pH 8.0 and digested separately overnight using lysC+Trypsin, chymotrypsin, GluC, elastase and a-lytic (Mass spectrum grade, Promega) at a ratio of 1:30 (w/w). Trypsin wasn't added until the pH was adjusted to 8.5 followed digested with LysC for 3 hours at pH 9.5, 37°C. The next day, the peptides were desalted using Monospin C18 column (GL Science,

Tokyo, Japan). The peptides were dried again, re-suspended in 0.1% formic acid water for *N*-linked glycan analysis. For *O*-linked glycan analysis, the five proteases-generated glycopeptides were treated with PNGase F prior to analysis to remove *N*-linked glycans.

SARS-CoV-2 virions preparation and Sample preparation for mass spectrometry analysis

SARS-CoV-2 is isolated from a SARS-CoV-2-infected patient for candidate vaccine preparation was provided by the Chinese Center for Disease Control and Prevention. SARS-CoV-2 virions were propagated in Vero cells (ATCC CCL-81)[1]. Viruses were cultured in a 10 L basket bioreactor at the temperature of 36 ± 1°C. The virus solution was harvested 48~72 h after inoculation and then was inactivated with β-propiolactone at a ratio of 1:4000 at 2~8°C for 20~24 h, followed by chromatography purification. All experiments involving infectious virus were conducted in approved biosafety level (BSL)-3 laboratory. Purification, concentration, biochemical analysis and sample preparation for electron microscopy of inactivated virions were carried out in a BSL-2 lab. To de-glycosylate S protein of virions, 20 μg purified virions sample was treated with 5 units PNGase F (New England Biolabs) at 37°C for 1 hour. 20 μg of treated and untreated samples were kept at 100°C for 15 min. De-glycosylation of S protein was characterized by 4 to 12% NuPAGE Bis-Tris gel (Invitrogen). The protein band was stained by silver stain (Thermo Fisher Scientific).

In-Gel Digestion

Bands of interest were excised, cut into smaller pieces (1 mm x 1 mm), rinsed with water and 2 x 30 min washed in 50 mM ammonium bicarbonate at 37°C. The gel pieces were then shrunk in 100 % acetonitrile (ACN) and shaken for 5 min. Solvent was removed, and gel pieces rehydrated in 10 mM DTT in 50 mM AmBic, followed by 40 min incubation at 60 °C. The gel pieces were again shrunk in 100 % ACN and shaken for 30 min. Solvent was removed, and gel pieces rehydrated in 55 mM iodoacetamide in 50 mM AmBic, followed by 40 min incubation at room temperature. The gel pieces were again shrunk in 100 % ACN and shaken for 15 min. The solvent was removed, gel pieces briefly rinsed with 50 mM AmBic and rehydrated in a small volume (10 µL) of 50 mM AmBic supplemented with LysC-trypsin, chymotrypsin and α-lytic at 37 °C for 18 hrs separately follow by PNGase F treatment at 37 °C for 4 hrs in O^{18} water. Peptides were stage-tip purified as previously described, dried, and reconstituted in 10 µL of 0.1 % formic acid prior to analysis.

LC-MS analysis

The peptides were first separated with a Thermo Scientific Ultimate 3000 RSLC nano LC system using trap-elute mode and then emitted into a Thermo Scientific Orbitrap Eclipse mass spectrometer (Thermo Fisher, San Jose).

Solvent A was 0.1% formic acid in water, while solvent B was 0.1% formic acid in 80% acetonitrile. After loading for 3 min with a flow rate of 10 µL/min in the trap column (Thermo Scientific™ Acclaim™ PepMap™ 100 C18, 75µm*2cm, 3µm, 100Å), all of the peptides were further eluted in the analytical column (Thermo Scientific™

Acclaim™ PepMap™ 100 C18, 75µm*25cm, 2µm, 100Å) with a flow rate of 250 nL/min using a gradient of 8% to 30% B for 127 min and 30% to 90% B for 16 min. This was followed by a 14 min washing step and a 10 min column equilibration step. To achieve "on the fly" glycopeptide identification, the stepped collision energy (SCE) HCD and HCDpdEThcD fragmentation method was employed for the data acquisition. The full scan mass spectra were recorded in positive ion mode over a scan range from 350 to 2000 m/z with a 60000 resolution (at 200 m/z). The AGC target and maximum injection time was set to 4e5 and 50 ms. A top 3 s setting was used for the dd-MS2 scan with a 30 s dynamic exclusion. The product dependent EThcD was triggered on the same precursor ion of HCD fragmentation with a 1.6 Da isolation window if one of the specific masses (204.0867, 138.0545, 366.1396, 243.026, 126.0551, 144.0655, 168.0655, 186.0761) was hit in the HCD spectra (30% NCE, 30000 resolution). Calibrated charge-dependent ETD parameters and 27% NCE HCD supplemental activation was used to obtain the EThcD spectra with resolution set to 30000 (at 200 m/z), AGC target set to 4e5, and maximum injection time set to 200 ms. The HCD collisional energy was applied with 10%, 15%, 20%, 25%, 30% and 35%. The stepped collisional energy (SCE) was set at 20%, 25% and 30% (+/- 10%).

Data analysis

The glycopeptide fragmentation data were extracted from the raw file using Byonic™ (Version 3.8.13) and Byologic® software (Version 3.8-11-x64; Protein Metrics Inc.) with the mass tolerance for the precursors and fragment ions set at ±15 and ±20 ppm,

respectively. Two missed cleavage sites were subjected to all enzyme digestion. The fixed modification was carbamidomethyl (C), the variable modifications included oxidation (M), and additional deamidation (+0.984016N) for O^{16} H_2O and deamidation (+2.988261N) for O^{18} H_2O, was added for the O glycosite search. In addition, the Protein Metrics 78 *O*-linked glycan library and 309 *N*-linked glycan library were specified as *O*-glycan modifications for the intact *O*-glycopeptides and the *N*-glycan modifications for the intact *N*-glycopeptides. A 1% false discovery rate (FDR) was applied. For the *N*-glycosylation identification, in addition to the NxS/T motif, the variable modification at any N was set in the modification panel and the 309 *N*-linked glycan library was used.

Four criteria were used for the characterization of *O*-glycopeptides: 1) the MS/MS spectra contains glycan or oxonium ion (i.e. feature B ions); 2) the MS/MS spectra contains feature Y ions; 3) isotope distribution of precursor is reasonable; and 4) the retention time of glycopeptides and un-glycopeptides is comparable. Peptide score was set at 150 and 200 for filtering *O*- and *N*-glycopeptides, respectively. The relative amounts of each *O*-glycosites were calculated by comparing the extracted chromatographic areas of glycosylated peptides to the total peptides containing the modified glycosites. All charge states for a single glycopeptide were summed. Furthermore, all of the glycopeptide-spectrum matches (GPSMs) must be manually examined in order to select the valid *O*-glycopeptides for the glycosites analysis. Diagnostic ions were used as the requisite criterion for the *O*-glycosites determination.

1. Wang, H., et al., *Development of an Inactivated Vaccine Candidate, BBIBP-CorV, with Potent Protection against SARS-CoV-2.* Cell, 2020. **182**(3): p. 713-721 e9.
